# Passion Fruit Seed Oil Modulates the Hepatic Metalloproteomic Profile of Selenium and Zinc in Laying Hens Under Heat Stress

**DOI:** 10.3390/ijms27041646

**Published:** 2026-02-08

**Authors:** Luane B. G. Andrade, Joyce A. Silva, Paola A. D. Rodrigues, Lais Garcia Cordeiro, Eduardo R. Silva, José C. S. Vieira, Marília A. R. Buzalaf, Sacaia Alvim Santos Romani, Alessandra Sussulini, Jiri Adamec, José R. Sartori, Pedro M. Padilha

**Affiliations:** 1 School of Veterinary Medicine and Animal Science, São Paulo State University (UNESP), Botucatu 18618-681, SP, Brazil; luane.andrade@unesp.br (L.B.G.A.); paola.damazio@unesp.br (P.A.D.R.); lais.cordeiro@unesp.br (L.G.C.); eduardo.rodrigues-silva@unesp.br (E.R.S.); cavalcante.vieira@unesp.br (J.C.S.V.); jose.sartori@unesp.br (J.R.S.); 2 Institute of Biosciences, São Paulo State University (UNESP), Botucatu 18618-693, SP, Brazil; joyce.silva@unesp.br; 3 Department of Biochemistry, Bauru School of Dentistry of Dentistry, University of São Paulo (USP), Bauru 17012-901, SP, Brazil; mbuzalaf@fob.usp.br; 4 Laboratory of Bioanalytics and Integrated Omics (LaBIOmics), Department of Analytical Chemistry, Institute of Chemistry, Universidade Estadual de Campinas (UNICAMP), P.O. Box 6154, Campinas 13083-970, SP, Brazil; l239805@unicamp.br (S.A.S.R.); sussulini@unicamp.br (A.S.); 5 School of Medicine, Louisiana State University Health Sciences Center (LSUHSC), New Orleans, LA 70112, USA; jadame@lsuhsc.edu

**Keywords:** phytogenic, metal binding proteins, chaperones, homeostasis, energy metabolism

## Abstract

Due to its antioxidant and immunomodulatory properties, using passion fruit seed oil (PFSO) is a promising strategy to mitigate the effects of heat stress in laying hens, potentially optimizing the absorption of essential minerals such as selenium (Se) and zinc (Zn). Therefore, this study investigated the profile of selenium- and zinc-binding proteins (Se/Zn-BPs) in the hepatic proteome of Lohmann White laying hens (26 weeks old, n = 96) subjected to heat stress and whose diet was supplemented with 0.9% PFSO, using a metalloproteomic approach that combined two-dimensional electrophoresis (2D PAGE), graphite furnace atomic absorption spectrometry (GFAAS), and liquid chromatography–tandem mass spectrometry (LC-MS/MS). The experimental design was a 2 × 2 factorial (temperature: thermoneutral/stress × diet: control/PFSO) design. After 84 days, liver samples were collected and subjected to metalloproteomic analyses. GFAAS analysis showed higher concentrations of Zn and Se in the protein pellets and in 11 specific protein spots of the supplemented groups (thermoneutral/PFSO and stress/PFSO). LC-MS/MS analysis identified 56 Se/Zn-BPs, with a predominance of heat shock chaperones (HSPs) and proteins involved in energy metabolism. In conclusion, PFSO supplementation modulates Se and Zn absorption, promoting a mineral balance that optimizes immune and antioxidant defense processes. This mechanism can lead to a positive impact on the health and productive performance of laying hens under heat stress.

## 1. Introduction

Brazilian fruit farming has stood out in agribusiness due to the wide variety of species cultivated under diverse climatic conditions [1]. As an example, yellow passion fruit, of the genus *Passiflora*, has more than 400 species, of which 120 are native to Brazil [2]. However, the industrial processing of passion fruit for juice production generates a significant volume of co-products/by-products. It is estimated that passion fruit residues (peel and seeds) constitute 90% of the by-products, representing approximately 40% of the total mass of the processed fruit [3]. This fruit has relevant nutritional characteristics, with a high content of vitamins, phenolic compounds, and carotenoids (responsible for its yellow color). Seeds are an alternative source of raw material, providing lipids and essential fatty acids (e.g., linoleic, oleic, and palmitic acids) [4]. Passion fruit seed residues demonstrate positive nutritional potential for poultry production, such as laying hens. Its composition, rich in bioactive compounds, can improve the health and performance of birds by reducing oxidative stress, stimulating the antioxidant defense system, and enhancing egg quality [5].

The worsening of climate change imposes heat stress as one of the most emblematic challenges for the poultry industry, especially in tropical and subtropical regions. This phenomenon has caused significant economic losses in several countries [6,7]. Production poultry are particularly vulnerable to heat stress due to physiological characteristics that limit their ability to dissipate heat [8]. Heat stress triggers a series of physiological disorders that adversely affect animal health and well-being. Intervention through nutritional strategies is an effective way to mitigate the effects of high temperatures.

For a more comprehensive understanding of the effects of heat stress on poultry health, it is crucial to apply molecular tools. These tools provide new insights into the animal biological system [9]. In this sense, proteomics is a technique that enables the optimized analysis and interpretation of results. When combined with analytical techniques, such as atomic spectrometry, proteomics enables a better understanding of the interactions between essential minerals and crucial proteins and/or enzymes in the antioxidant system of poultry. Supplementation with phytogenic compounds in the diet can increase the bioavailability of essential minerals for cellular defense against oxidative stress, such as Se and Zn. This, in turn, can induce increased expression of antioxidant system proteins and, consequently, mitigate the deleterious effects of heat stress, a major challenge in poultry farming [10].

Thus, supplementation with passion fruit seed oil (PFSO) can modulate the levels of Se and Zn in the liver tissue of laying hens, contributing to a reduction in or inhibition of oxidative stress induced by heat stress. In this context, metalloproteomics—which investigates how metals influence the structure, function, and homeostasis of proteins in the organism [11]—offers a robust approach to further elucidating these mechanisms. Based on the above context, this study aimed to investigate, through a metalloproteomic approach, the presence and profile of *Se/Zn-binding proteins* (*Se/Zn-BPs*) in the hepatic proteome of laying hens subjected to heat stress and whose diet was supplemented with PFSO.

## 2. Results

### 2.1. Se and Zn Determination in Protein Pellets from Liver Tissue

Table 1 presents the mean concentrations of Se and Zn in protein pellet samples, determined by GFAAS and FAAS, respectively, for SC (stress control without PFSO supplementation in the birds’ diet), TC (thermoneutral control without PFSO supplementation in the birds’ diet), SPFSO (heat stress with PFSO supplementation in the birds’ diet), and TPFSO (thermoneutral with PFSO supplementation in the birds’ diet) treatments. It was observed that Se and Zn concentrations in the protein pellets were significantly higher in groups whose diets were supplemented with PFSO (SPFSO and TPFSO groups) compared to SC and TC groups (*p* < 0.05). Furthermore, the TC group, which was not subjected to heat stress, showed higher concentrations of Se and Zn than the SC group, which was exposed to heat stress (*p* < 0.05). However, there was no statistical difference in Se and Zn concentrations between SPFSO and TPFSO groups (*p* > 0.05). The results of Se and Zn determinations in the liver tissue samples, detailed in Tables S2–S5 for birds in the SC, TC, PFS (passion fruit stress), and PFT (passion fruit thermoneutral) treatments, mirrored the pattern observed in the protein pellets.

### 2.2. Image Analysis of 2D PAGE Gel and Se and Zn Mapping in the Protein Spots

**Figure 1:**
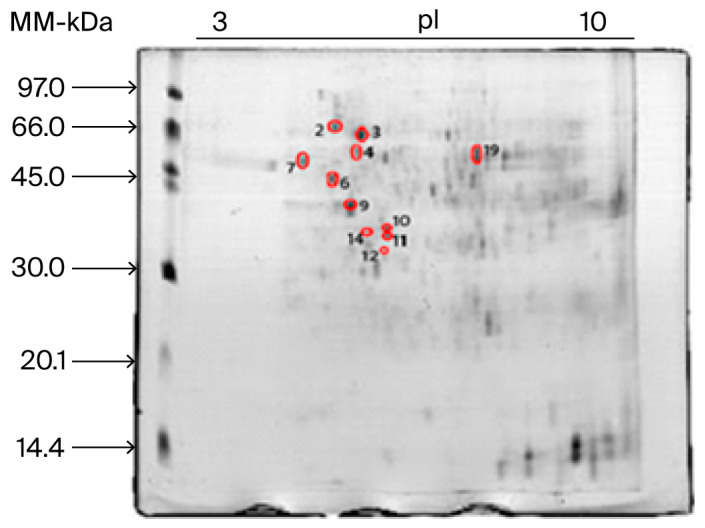
Representative 2D-PAGE gel of the hepatic proteome (TM group). Sample: liver tissue from laying hens fed a diet supplemented with OSM. Spots highlighted with a red circle contain Se and Zn, as determined by GFAAS. Raw replica figures of 2D PAGE gels of SC, SPFSO, TC, and TPFSO treatments are shown in Figures S1–S4 (Supplementary Materials).

A representative image of one of the 2D PAGE gels from the TM group triplicates is shown in Figure 1. In this gel image, out of a total of 246 spots, 11 spots (highlighted with a red circle) were found to contain Se and Zn, as determined by GFAAS.

Table 2 summarizes the Se and Zn concentrations, as determined via GFAAS, in the 11 spots highlighted with a red circle in Figure 1.

### 2.3. Se and Zn Determination in Passion Fruit Seed Oil and Identification of Volatile Compounds Using GC-MS

The determination of Se and Zn concentrations in PFSO was performed in triplicate using GFAAS and FAAS, respectively. The values obtained in the replicates, as well as the average concentrations of the two minerals, are summarized in Table 3.

HS-SPME-LC-MS analysis of PFSO revealed the presence of several saturated fatty acids, specifically decanoic (capric), pentadecanoic, and hexadecanoic (palmitic) acids. These compounds are known in the literature for their therapeutic and antioxidant properties [12]. In addition to these fatty acids, the analysis also identified various volatile compounds, including terpene limonene (1-methyl-4-(prop-1-en-2-yl)cyclohex-1-ene); diol 2,2,4-trimethyl-1,3-pentanediol; methyl salicylate ester; phenylethyl alcohol; terpenic alcohol linalool (7-methyl-3-methyleneocta-4,6-dien-2-ol); and bicyclic monoterpene 3-carene (3,7,7-trimethylbicyclo[4.1.0]hept-3-ene). Collectively, these identified compounds exhibit therapeutic and antioxidant properties [12,13].

It is important to highlight that the results of PFSO analysis using HS-SPME-LC-MS did not indicate the presence of the polyunsaturated fatty acids: oleic acid (*cis*-9-octadecenoic acid, omega-9) and linoleic acid (9,12-octadecadienoic acid, omega-6). The absence of these compounds can be attributed to their thermal degradation during HS-SPME-LC-MS analysis, where the sample is subjected to a gradually increasing temperature gradient. Due to their unsaturations, these fatty acids are highly susceptible to oxidation as the temperature increases [14]. These fatty acids are reported in the literature to offer significant nutritional benefits and represent approximately 22% (omega-9) and 61% (omega-6) of the PFSO composition, respectively [13]. This composition was confirmed by GC-MS analyses using a continuous low-temperature phase-transition extraction strategy, which further supports its thermal lability [14]. The raw data from the composition analysis of PFSO volatile compounds using HS-SPME-LC-MS are summarized in the Supplementary Materials (raw data—HS-SPMELC-MS).

### 2.4. Characterization of Protein Spots Associated with Se and Zn via LC-MS/MS and Functional Analysis of Se/Zn-Binding Proteins Using String Software

**Figure 2:**
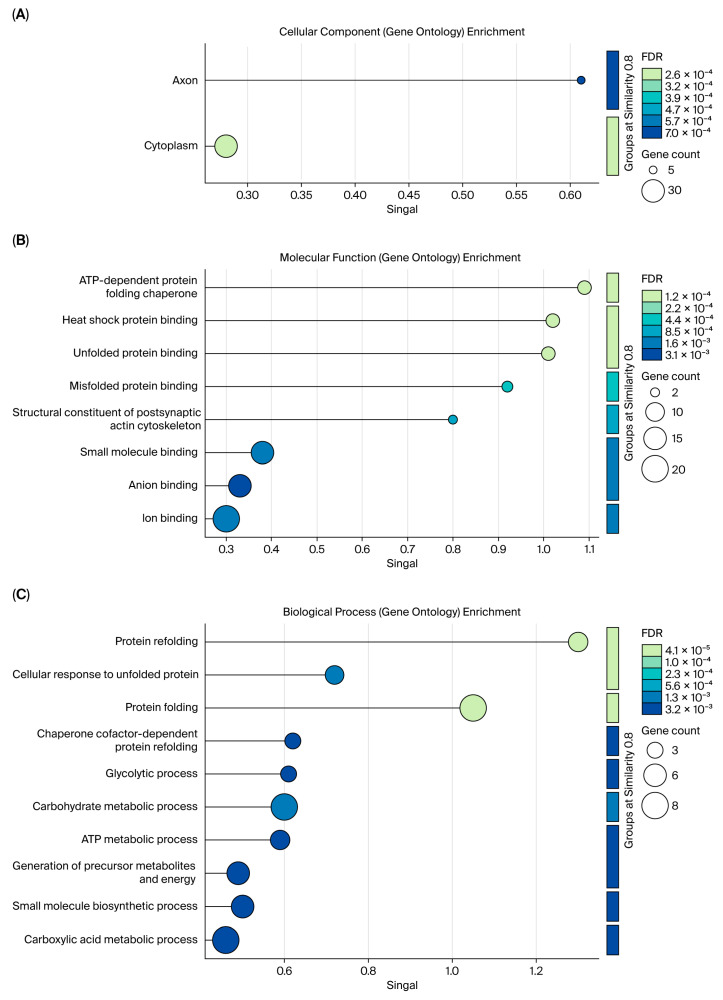
Results of the functional analysis of Se/Zn-metal binding proteins obtained via STRING software. The results are categorized into (**A**) cellular components; (**B**) molecular functions; and (**C**) biological processes.

**Figure 3:**
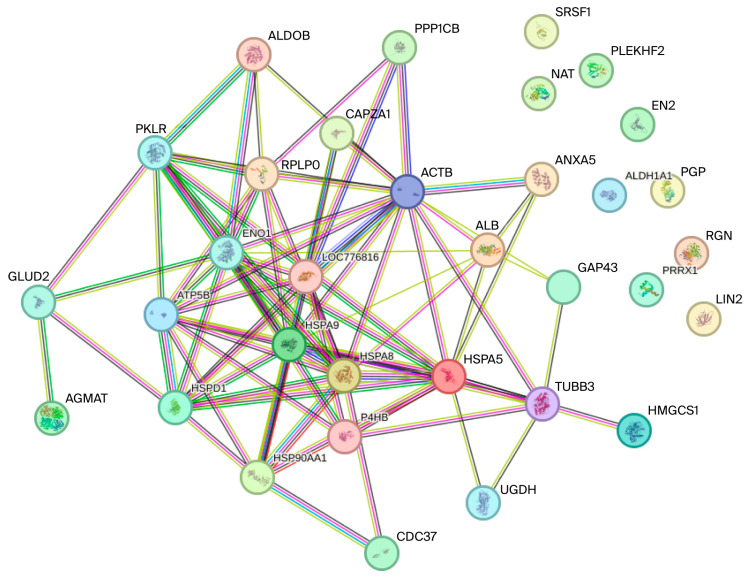
Protein–protein interaction network of Se/Zn-BPs constructed using STRING software.

The characterization of protein spots associated with Se and Zn was performed using LC-MS/MS, which enabled the identification of Se/Zn-binding proteins (Se/Zn-BPs) listed in Table 4. The raw characterization data for the protein spots are shown in Table S6 (Supplementary Materials). Subsequently, functional analysis was conducted on these *Se/Zn-BPs* using their FASTA sequences via the STRING software (version 12.5, 2025). This analysis enabled the categorization of the proteins based on their biological process, cellular component, and molecular function, as well as the construction of a protein–protein interaction network. The results of the functional analysis are presented in Figure 2 and Figure 3.

## 3. Discussion

### 3.1. Determination of Se and Zn and Volatile Compounds in PFSO

The concentrations of Se and Zn in protein pellets were significantly higher in the TPFSO and SPFSO groups compared to the TC and SC groups (Table 1, *p* < 0.05). The literature suggests that passion fruit seed oil (PFSO) is not a primary source of minerals. Microminerals are predominantly found in the solid components of the seed meal, not in the extracted oil. In particular, although there are no direct reports of Zn content in PFSO, concentrations ranging from 4.6 to 56 mg kg^−1^ have been reported in *Passiflora edulis f. edulis* seeds [15]. The average Zn concentration determined in the *Passiflora edulis f. flavicarpa* PFSO in this study was 17.12 ± 0.19 mg kg^−1^. Assuming that the entirety of the seed’s Zn content was extracted into the oil, this result is consistent with the levels reported for the whole seed in the literature [15,16]. Studies on Se content in passion fruit and its by-products are scarce, mainly limited to data on passion fruit juice, which indicate concentrations between 6.0 and 10.0 µg kg^−1^ for *P. edulis f. edulis* and *P. edulis f. flavicarpa* [15,17]. The average Se concentration determined in the PFSO in this work was 113.1 ± 0.35 µg kg^−1^. As the existing literature focuses on fruit juice and lacks specific data on Se content in PFSO, a direct comparison is not feasible. Consequently, the data reported in Table 3 constitute a novel finding regarding the mineral profile of passion fruit seed oil.

The TPFSO and SPFSO treatment groups, which included OSM in the birds’ diet, showed Se and Zn concentrations in protein pellets that were 20% to 45% and 45% to 75% higher, respectively, compared to the control groups (TC and CE) (*p* < 0.05) (Table 1). The increase in Se and Zn concentration in the TPFSO and SPFSO treatment groups cannot be attributed to the inherent concentrations of these minerals in PFSO, as these are insignificant when compared to the concentrations in the experimental diets (Se—mg kg^−1^; Zn—2000 mg kg^−1^; Table S1).

The PFSO used in this study, derived from *Passiflora* species cultivated in Brazil, has high concentrations of vitamin E [18]. The literature reports that, in animal cells, vitamin E can increase the transport and uptake of Se by intestinal cells. This positive interaction is synergistic: while vitamin E neutralizes lipid peroxides in cell membranes, Se-activated enzymes, such as glutathione peroxidase (GPx), convert hydroperoxides into inert alcohols in the cytoplasm. Thus, the combined action of both prevents lipid peroxidation more effectively. Furthermore, by combating free radicals in cell membranes, vitamin E can decrease the demand for and activity of Se and its enzymes in combating oxidative stress [19,20]. Therefore, supplementation with PFSO, which has a high concentration of vitamin E (approximately 5 g kg^−1^ [18]), in the diet of the TPFSO and SPFSO groups may have contributed to the modulation of the Se concentration (6 mg kg^−1^) in the experimental diet, promoting an ideal balance between the two nutrients and, consequently, favoring a more effective antioxidant response.

Regarding Zn, two pioneering studies [21,22] report that vitamin E deficiency can reduce plasma Zn levels, suggesting that Zn may be redistributed to other tissues to compensate for vitamin E’s antioxidant deficiency. In this context, the two nutrients act synergistically as antioxidants, protecting cells against oxidative stress. Another pioneering study [23] conducted in rats demonstrated that the concentration of vitamin E absorbed into mesenteric lymph is mediated by Zn concentration, and that Zn deficiency reduces the lymphatic absorption of alpha-tocopherol (vitamin E). Thus, oxidative alterations in Zn-deficient organisms may be related to the availability of vitamin E in the diet. The TPFSO and SPFSO treatments showed the same behavior as Se in relation to Zn; that is, the inclusion of PFSO in the birds’ diet modulated the absorption balance of the high concentration of Zn present in the experimental diets (2000 mg kg^−1^, Table S1). This modulation likely occurred to maintain the necessary balance between Zn and the vitamin E present in PFSO, enabling their joint action in the birds’ antioxidant defense systems and thereby protecting cells from oxidative stress.

It should also be noted that the TPFSO and SPFSO treatments, through the inclusion of PFSO in the diet, provide fatty acids with immunological and antioxidant properties [12,13,24]. These include polyunsaturated fatty acids (such as oleic and linoleic acids) and saturated fatty acids (such as pentadecanoic and hexadecanoic acids). These fatty acids can affect the birds’ immune system, modulating the production of antibodies such as IgM and IgG. Furthermore, they can contribute to the indirect neutralization of oxidants by modulating the expression of antioxidant enzymes, such as superoxide dismutase and catalase [25,26,27,28].

Particularly in laying hens (the subject of this study), a study by Gao et al. (2022) [29] demonstrated that the inclusion of 1.5% (m/m) oils with different levels of fatty acids in the diet had a positive effect on the birds’ antioxidant response, which also contributed to egg quality. Thus, the association of the immunological and antioxidant properties of the compounds present in the composition of PFSO, by modulating the absorption of Se and Zn, establishes a balance in the concentrations of these two minerals. This balance, in turn, favors the action of Se and Zn in the immunological and antioxidant defense processes of laying hens, which is expected to have a favorable impact on egg production.

### 3.2. Characterization and Identification of Se/Zn-Associated Protein Spots via GFAAS and LC-MS/MS

GFAAS analysis indicated the presence of Se and Zn in 11 protein spots (spots 02, 03, 04, 06, 07, 09, 10, 11, 12, 14 and 19; Table 2). The concentrations of the two minerals followed the same pattern as the results obtained for the protein pellets, namely, higher concentrations of Se and Zn in the TPFSO and SPFSO groups. In these two groups specifically, the 11 spots showed Se and Zn average concentrations between 112.7 ± 6.183 to 132.6 ± 1.36 mg kg^−1^ and 104.3 ± 1.14 to 125.4 ± 1.31 mg kg^−1^, respectively. Within these 11 protein spots, 52 *Se/Zn-binding proteins* (Se/Zn-*BPs*) were subsequently identified via LC-MS/MS (Table 4).

Among the identified Se/Zn-*BPs*, heat shock chaperones (HSPs) (spots 2, 3, 4, 6 and 14) stand out. These proteins are directly involved in heat stress and are therefore of fundamental importance to this study, which involves experiments with laying hens subjected to heat stress [30]. In addition to HSPs, albumin—a protein synthesized in the liver and released into the bloodstream to perform its functions [31]—was also identified in spots 2, 3 and 4.

Also noteworthy are the following enzymes, which were identified in spots 4, 6, 7, 10, 14 and 19: hydroxymethylglutaryl-CoA synthase cytoplasmic (HMGCS1), ATP synthase F(1) complex catalytic subunit beta mitochondrial (ATP5F1B), protein disulfide isomerase (P4HB), fructose-bisphosphate aldolase B (ALDOB), large ribosomal subunit protein uL10 (RPLP0), glycerol-3-phosphate phosphatase (PGP), glutamate dehydrogenase 1 mitochondrial (GLUD1), alpha-enolase (ENO1), beta-enolase (ENO3), pyruvate kinase (PKM), UDP-glucose 6-dehydrogenase (UGDH), and aldehyde dehydrogenase 1A1 (ALDH1A1). These enzymes, while not all acting in the same pathway, participate in metabolic processes that form a network of interdependent cellular activities. These activities involve energy pathways (such as glycolysis and oxidative phosphorylation), lipid metabolism, and regulation of the cellular stress response. Such processes are essential for the cellular response to heat stress and subsequent oxidative stress [32,33,34].

Key cytoskeletal proteins were identified at spots 6, 9, 11, 12, and 14, including actin cytoplasmic 1 (ACTB), actin cytoplasmic 2 (ACTG1), tubulin beta-4 chain, and F-actin-capping protein subunit alpha-1 (CAPZA1). As the core components of the cellular structural and functional network, these proteins are critical to the cell’s response to heat stress in laying hens [35,36]. Heat stress leads to oxidative stress, an imbalance between free radical production and antioxidant capacity, ultimately causing cellular damage. A consequence of this damage is the denaturation and/or aggregation of cytoskeletal proteins like actins and tubulins [37,38].

Thus, key cytoskeletal proteins (actin cytoplasmic 1, actin cytoplasmic 2, tubulin beta-4 chain, and F-actin-capping protein subunit alpha-1) were identified as *Se/Zn*-*binding proteins* (Se/Zn-BPs) along with heat shock chaperones (HSPs), albumin, and several essential metabolic enzymes (such as HMGCS1, ATP5F1B, and enolases). This broad identification suggests a crucial interconnection between diet and cellular capacity to resist stress. The inclusion of PFSO in the diet (TPFSO and SPFSO treatments), which is rich in vitamin E and fatty acids, modulates the balance of Se and Zn concentrations, thereby ensuring an adequate supply of these minerals to these key proteins. By optimizing the absorption of these minerals and the functionality of these proteins—which are vital for energy metabolism (enzymes), nutrient transport (albumin), protein stability under stress (HSPs), and cellular structure/integrity (cytoskeletal proteins)—the PFSO-based diet supports the antioxidant defense network of laying hens. This integrated mechanism of action enables birds to better manage heat stress and mitigate oxidative damage, which, in turn, could positively affect the immune response and productive performance of laying hens.

The main objective of this study is to investigate how the phytogenic properties of PFSO modulate Se and Zn concentrations in laying hens, aiming for a balance that optimizes immunological and antioxidant defenses and enhances egg production. In this context, the following discussion examines the results of LC-MS/MS metalloproteomic characterization (Table 4), focusing specifically on HSPs (heat shock proteins) identified as *Se/Zn-binding proteins* (*Se/Zn-HSPs*).

The liver is the main metabolic organ, essential for energy and lipid metabolism [39], and heat stress can directly impair its basic functions. In the fractionation of the laying hen liver proteome by 2D PAGE, the chaperones (HSPs) HSPA5, HSPA8, HSP70, HSP90AA1, HSPA9, HSPD1, and the co-chaperone CDC37 were identified in spots 2, 3, 4, 6, and 14. The main function of these chaperones is to protect proteins and enzymes under stress. HSPs are upregulated in response to a wide variety of stresses—both environmental (such as heat, cold, water loss, oxygen restriction, toxic metals, and chemical pollutants) and internal (such as infection and inflammation)—that disrupt the normal conformation or function of cellular proteins [39,40,41]. The molecular chaperone BiP (HSPA5), also known as GRP78, was identified in protein spots 2 and 3. This protein primarily resides in the endoplasmic reticulum (ER), where it is crucial for cellular homeostasis, maturation, and correct protein folding [42,43]. The presence of BiP in the liver of laying hens indicates cellular stress, as its expression is induced by ER stress, typically triggered by the accumulation of misfolded proteins [44]. This aligns with previous findings where exposure of birds to high temperatures caused hepatic degeneration and increased GRP78/HSPA5 expression [45]. Furthermore, a recent proteomic study [46] reinforced HSPA5’s potential as a molecular indicator of heat stress, identifying it with a positive fold change in stressed laying hens’ liver tissue. Complementing the role of BiP (HSPA5) as a cellular stress indicator, spots 2 and 3 (containing HSPA5) showed Zn and Se concentrations approximately three times higher in the SPFSO group compared to the SC group (GFAAS determinations; Table 2). Although the literature does not document the co-occurrence of Zn and Se in *metal-binding proteins*, the identification of HSPA5 associated with these minerals (termed *Se/Zn-HSPA5*) and the increase in their concentrations under stress suggest that *Se/Zn*-*HSPA5* may constitute a novel molecular marker of heat stress.

The cytosolic chaperones HSPA8, HSP70, HSP90AA1, HSPA9, and HSPD1 and the co-chaperone CDC37 were identified in spots 2, 3, 4, 6, and 14. Primarily induced by heat, they are also triggered by various other types of stress [47]. HSPs are classified by molecular mass (10 to 150 kDa) [48,49]. Their essential function is cell protection: they mediate protein folding and unfolding, prevent aggregation, and repair unstable proteins, serving as the first line of defense against stress [50,51]. Following the HSP response, the antioxidant system—the second line of defense—is activated. In this system, Se and Zn act as cofactors for crucial enzymes (glutathione peroxidase and superoxide dismutase, respectively) [52,53,54,55,56]. Spots 2, 3, 4, 6 and 14, where HSPs were identified, also showed Se and Zn concentrations three times higher in the SPFSO group compared to the SC group. This finding is partially supported by literature reporting Zn associated with HSP90 and Hsc70 in tilapia [57]. Although direct Se coordination at HSP binding sites is thermodynamically unfavorable [58], HSPs can interact non-covalently with selenoproteins (that can act as their substrates) during folding or under stress [59].

A hypothesis rooted in coordination chemistry suggests that the formation of a binary Se-Zn complex is thermodynamically possible [60]. This complex would bind to the sulfhydryl groups (R-S-H) and/or thioether groups (R-S-CH_3_) present in the FASTA sequences of HSPs [60,61,62]. Selenium, found in amino acids like selenocysteine (NO_2_H_6_C_3_-Se-H) and/or selenomethionine (C_4_H_8_NO_2_-Se-CH_3_), possesses available electron pairs that can coordinate Zn^2+^ ions, forming complexes such as selenocysteine-Zn or selenomethionine-Zn. In these resulting complexes, Zn^2+^ would coordinate the R-S-H and/or R-S-CH_3_ groups of the HSPs, forming complexes of the type P-S-Zn-Se-R (where P is the HSP, and R is the carbon chain of the selenium-containing amino acid). This unique linkage would thus form *metal-binding proteins* (Se/Zn-*HSPs*).

In general, this study confirms that PFSO dietary supplementation in laying hens confers protection against heat stress, mediated by the activation of molecular mechanisms and the PFSO’s antioxidant effect. This action is reflected in alterations within the hepatic proteome—the main metabolic center of birds [63,64,65,66]. Mitigation of oxidative stress leads to the modulation of Se and Zn absorption and metabolism, thereby balancing their concentrations and incorporation. This modulation is evidenced in the hepatic proteome, where the supplemented groups (TPFSO and SPFSO) showed Se and Zn concentrations approximately threefold higher than those of the control groups (TC and SC). This enhanced micromineral metabolism led to a novel discovery: the formation of *metal-binding HSPs* (Se/Zn-*HSPs*), as indicated by metalloproteomic data. The formation of *Se/Zn*-*HSPs* suggests a significant increase in the resilience of the birds to heat stress, consequently inducing notable improvements in overall health and productive performance.

### 3.3. Functional Analysis of Se/Zn-Binding Protein

In the analyzed interaction network (Figure 3), four proteins (HSPD1, SPA9, HSPA8, and HSPA5) belonging to the heat shock protein (HSP) chaperone family are observed. HSPs play an essential role in cellular homeostasis and protein folding [67,68]. These chaperones are diversely located, encompassing the cytosol, mitochondria, cytoplasm/nucleus, and endoplasmic reticulum, as detailed in Graph A [69]. Regarding biological processes (Graph C), many proteins require molecular chaperones to ensure proper folding within the required biological timeframe [70]. Among the expressed proteins, HSPA8 (a 71 kDa protein) stands out, exhibiting diverse cellular functions, including protecting the proteome against damage from stressors, assisting in the transport of newly synthesized polypeptides, participating in the formation and separation of protein complexes, and ensuring correct protein folding. Collectively, these proteins perform differentiated and coordinated functions, thereby ensuring organismal homeostasis [71].

When analyzing the efficiency of homeostatic mechanisms, the participation of minerals such as Se and Zn is prominent. Se plays a crucial role in the activity of glutathione peroxidase (GPx), a selenoenzyme fundamental to the first line of antioxidant defense and essential for scavenging reactive oxygen species (ROS). Consequently, GPx protects the hepatic proteome and cell membranes against oxidative damage, a response that is complemented by HSPs. Zn, conversely, acts as a cofactor for important enzymes involved in the response to oxidative stress, such as superoxide dismutase (SOD). SOD further complements the protection provided by HSPs against cellular damage [72,73,74].

The proteins PKM, ALDOB, EN01, and ATP5F1B, also identified in Figure 3, are directly related to energy metabolism. PKM (pyruvate kinase) is the main enzyme in glycolysis, catalyzing the conversion of phosphoenolpyruvate (PEP) and ADP into pyruvate, which results in ATP production [75]. Its role in the final stage of glycolysis is critical for energy production in tissues with high metabolic activity, such as the liver [76]. ALDOB (fructose-bisphosphate Aldolase B) is a fundamental enzyme for glucose homeostasis in the liver. It plays a crucial role in fructose metabolism, cleaving the metabolic intermediate fructose 1-phosphate (Fru-1-P) into dihydroxyacetone phosphate (DHAP) and glyceraldehyde [77]. EN01 (Alpha-enolase or Enolase 1) is a protein with multiple activities; its primary function is the catalysis of glycolysis. Beyond its basic function, EN01 participates in several other intracellular and extracellular activities, including serving as a plasminogen receptor on the cell surface, assisting in microtubule organization, and maintaining mitochondrial membrane stability in the cytoplasm [78]. Considered a key protein in energy metabolism, ATP5F1B (ATP synthase subunit beta, mitochondrial) is an essential component of the mitochondrial ATP synthase complex. The purpose of ATP5F1B is to catalyze the phosphorylation of adenosine diphosphate (ADP), thereby generating ATP. In this way, it provides the energy necessary to maintain cell function and survival [79,80].

Se and Zn are vital minerals for the efficiency and regulation of energy metabolism. Selenium is incorporated into selenoproteins, such as glutathione peroxidases (GPx), which protect the mitochondrial apparatus (where ATP5F1B acts) from oxidative stress generated during energy production, thereby maintaining cellular integrity. Zinc, in turn, acts as a cofactor and modulator for numerous enzymes, including those involved in glycolysis (PKM, ALDOB, EN01), and is fundamental for maintaining the structure and catalytic activity of these metabolic pathways. Therefore, a deficiency in Zn or Se can lead to mitochondrial dysfunction and compromise ATP production [81].

Overall, the coordination of cellular homeostasis and energy metabolism (glycolysis and ATP synthesis)—mediated by crucial proteins like HSPs, PKM, and ATP5F1B—is intrinsically dependent on micronutrient balance. This is evidenced by the adaptive role of the *Se/Zn-binding protein* (*Se/Zn-BP*) complex, which proves indispensable for both the regulation of cellular processes and energy provision to laying hens under heat stress, ultimately ensuring proteome integrity and metabolic efficiency.

## 4. Materials and Methods

The experiment was conducted at the Laboratory of Poultry Nutrition at the College of Veterinary Medicine and Animal Science (FMVZ), São Paulo State University (UNESP), Botucatu. The protocol was approved by the institution’s Ethics Committee on the Use of Animals—CEUA (protocol No. 221/2022) in compliance with animal experimentation and welfare standards. Birds were vaccinated against Marek’s disease, Avianpox (Fowlpox), and Gumboro disease (Infectious Bursal Disease—IBD) upon hatching. Until the 26th week of age, they received further vaccinations via the Oculonasal route (eye drop)—Gumboro disease, Infectious Bronchitis, and Newcastle disease; Wing Web (Stick) route—Avianpox (Fowlpox/Bouba); and intramuscular route (oily/inactivated vaccines)—Egg Drop Syndrome (EDS) and Infectious Coryza [82].

### 4.1. Animal Experimentation

#### 4.1.1. Experimental Configuration

A total of 96 Lohmann White laying hens (26 weeks old) were used in a completely randomized design (CRD) experiment with a 2 × 2 factorial arrangement. The factors included two diets (control—C, with 0.9% passion fruit seed oil—PFSO) and two temperatures (thermoneutral—T and warm (heat stress)—S). Thus, four groups were formed, as follows: SC—stress control without PFSO supplementation in the birds’ diet; TC—thermoneutral control without PFSO supplementation in the birds’ diet; SPFO—heat stress with PFSO supplementation in the birds’ diet; TPFSO—thermoneutral with PFSO supplementation in the birds’ diet. The birds, which had been vaccinated at one day of age (Gumboro, Marek’s disease, and Fowlpox/Avianpox), were distributed to 24 galvanized wire cages (0.5 m × 0.5 m × 0.6 m), with six cages per treatment. Water and feed were provided ad libitum in nipple drinkers and trough feeders, respectively [46].

#### 4.1.2. Diets and Lighting

The isocaloric, isonutritive diets were formulated from corn and soybean meal, in accordance with recommendations by Rostagno et al. (2017) [83] (Table S1). Soybean meal replaced soybean oil in the formulation, applying a correction for the apparent metabolizable energy (AME) value of 9378 kcal/kg [84]. The lighting program followed the lineage management guide, providing 16 h of daily light (natural and artificial).

#### 4.1.3. Climatic Conditions

The experiment lasted 84 days. The birds were kept in two distinct climatic chambers [46,82]:

1. Thermoneutral (T) Chamber: maintained in the thermal comfort zone (20 °C to 25 °C).
2. Heat Stress (S) Chamber: subjected to thermal stress (31 °C to 32 °C) for 8 h daily (8:30 a.m. to 4:30 p.m.). The temperature was reduced to the thermoneutral level after this period.

Temperature and relative humidity (RH) were monitored daily at bird height using maximum and minimum thermometers and HOBO electronic sensors (One-Time Data Loggers, Elitech LogEt 6, Xuzhou-China).

### 4.2. Sample Collection

At 84 days of age, the birds were individually identified and fasted for 8 h. For liver tissue collection, 1 bird from each experimental unit (totaling 6 per treatment) was euthanized via cervical dislocation, selecting animals with a weight close to the average of their respective group. Each liver fragment was sectioned into slices and immediately transferred to cryotubes. The samples were then stored in liquid nitrogen and transported to the Bioanalytic and Metalloproteomic Laboratory (LBM) of the Department of Chemistry and Biochemistry at IBB/UNESP, Botucatu/SP-Brazil [46,82]. Considering that the concentrations of Se and Zn in the six birds from each of the groups (SC, TC, SPFSO, and TPFSO) did not show significant differences (*p* > 0.05; Tables S2–S5, Supplementary Materials), four biological pools were created from the individual liver tissue samples, corresponding to the four treatments studied. Each pool was generated by homogenizing the liver samples from 6 birds in the same treatment group.

### 4.3. Protein Extraction and Precipitation

To extract proteins from liver tissue, 150 mg aliquots from each pool were placed into 2 mL microtubes and homogenized with 1000 µL of ultrapure water (18.2 MΩ cm^−1^) using an OMNI-BEAD RUPTOR 4 disruptor (Kennesaw, GA, USA). The resulting homogenates were chilled for 5 min before being centrifuged at 12,000× *g* for 15 min at 4 °C (Hettich Universal 320R, Andreas Hettich GmbH & Co. KG, Tuttlingen, Germany). For precipitation, 200 µL of the supernatant was mixed with 400 µL of ice-cold 80% (*v*/*v*) acetone and incubated at 4 °C for 90 min. Following pellet formation, a second centrifugation step (12,000× *g*, 15 min, 4 °C) was performed, and the supernatants were removed to isolate the protein pellets [10,46,57].

#### Determination of Total Protein

The protein content of the resulting pellets was quantified via the Biuret method, employing bovine serum albumin (BSA) as the calibration standard [85]. A linear calibration curve was constructed between 10 and 100 g L^−1^. For the assay, 50 µL of either sample or standard was combined with 2.5 mL of Biuret reagent and incubated at 32 °C for 10 min in a water bath. After cooling to room temperature for 5 min, the absorbance was measured at 545 nm using a UV/Visible spectrophotometer with 1 cm path length cuvettes [57].

### 4.4. Two-Dimensional Electrophoresis (2D-PAGE)

Following total protein quantification, proteome fractionation was performed via two-dimensional polyacrylamide gel electrophoresis (2D-PAGE). Protein pellets were reconstituted in a lysis buffer (7 mol L^−1^ urea, 2 mol L^−1^ thiourea, 2% (m/*v*) CHAPS, 0.5% (*v*/*v*) ampholytes pH 3–10, and 0.002% (*m*/*v*) bromophenol blue) to reach a final concentration of 1.50 mol L^−1^. Isoelectric Focusing (IEF) was initiated by loading 250 µL aliquots (375 µg of protein) onto 13 cm immobilized pH gradient strips (pH 3–10) using an EPS 1001 system (Amersham Biosciences, Amersham, UK). Post-IEF, the strips underwent a two-stage equilibration under gentle shaking (15 min each): first in dithiothreitol (DTT) for disulfide bond reduction, followed by iodoacetamide for thiol group alkylation to inhibit reoxidation. The second dimension (separation by molecular mass) was performed by transferring the strips to 12.5% (*m*/*v*) polyacrylamide gels. Electrophoresis was conducted using an Ettan™ DALT six system (GE Healthcare, Uppsala, Sweden) at 100 V for 30 min, then 180 V for 4.5 h, alongside a molecular weight marker (14.4–97.0 kDa). Finally, gels were fixed (10% (*v*/*v*) acetic acid/40% (*v*/*v*) ethanol) and stained with colloidal Coomassie G-250 for 72 h. After aqueous destaining, gel images were captured and processed with Image Master Platinum v.7.0. This analysis allowed for spot detection, replicate matching, and the determination of isoelectric points (pI) and molecular masses (MM) [10,57,86].

### 4.5. Se and Zn Determination in Liver Tissue, Pellets, and Protein Spots

Se and Zn concentrations in liver tissues and protein pellets were determined using graphite furnace atomic absorption spectrometry (GFAAS) and Flame Atomic Absorption Spectrometry (FAAS), respectively. A SHIMADZU (Osaka, Japan) model AA-6800 atomic absorption spectrometer—equipped with a Self-Reversal (SR) method background corrector, a pyrolytic graphite tube with an integrated platform, and an ASC-6100 autosampler—was used for the measurements. SHIMADZU (Osaka, Japan) hollow cathode lamps for Se and Zn, operated at a current of 12 mA, were used. The wavelengths were 196.0 nm and 213.9 nm for Se and Zn, respectively, with a spectral resolution of 0.5 nm. Argon was used as the inert gas, maintaining a constant flow of 1 L min^−1^ throughout the heating program, except during the atomization stage, when the gas flow was interrupted. Absorbance signals were measured as peak areas. The sample digestion involved an oxidizing mixture of 15 mol L^−1^ HNO_3_ (J.T. Baker, Phillipsburg, NJ, USA) and 0.02 mol L^−1^ KMnO_4_ (Merck, Rahway, NJ, USA) in a 1.0:0.5 (*v*/*v*) ratio. Approximately 100 mg of tissue or 40 mg of protein pellet was weighed into digestion vessels, treated with the oxidizing solution, and heated at 120 ± 2 °C until a clear or pale purple extract was obtained. These extracts were diluted to 5.00 mL with ultrapure water (18.2 MΩ cm^−1^) prior to spectroscopic analysis [87,88,89].

For metal mapping of protein spots across the SC, TC, SPFSO, and TPFSO groups, GFAAS was employed after spot excision and acid digestion, following the aforementioned protocol. The specific GFAAS parameters for Se and Zn quantification followed established methods by Lima et al. (2020) and Bataglioli et al. (2024) [57,89]. Quantitation was based on analytical curves generated from Titrisol MERCK standard solutions 1000 mg L^−1^ (Merck, Rahway, NJ, USA) diluted in 0.10 mol L^−1^ HCl (Merck, Rahway, NJ, USA). Instrumental operating conditions, blank preparations, and linear ranges were adopted from Neves et al. (2009) and Bataglioli et al. (2024) [88,89]. Analytical validation of results was performed using the DOLT-4 certified reference material (dogfish liver certified reference material for trace metals—the National Research Council of Canada). Following the mineralization process, Se and Zn recovery rates of 98.40% and 98.70%, respectively, were achieved relative to the certified values [10,11].

### 4.6. Characterization of Se/Zn-Associated Protein Spot via Liquid Chromatography–Tandem Mass Spectrometry (LC–MS/MS)

Selenium- and zinc-associated protein spots were manually recovered from the gels with a scalpel and fragmented into 1 mm^3^ pieces. Following transfer to microtubes with 1 mL of 5% (*v*/*v*) acetic acid, the samples underwent decolorization, reduction, alkylation, and digestion using a 10 ng mL^−1^ trypsin solution. The resulting peptide extracts were transferred to clean vials and reduced to approximately 12 µL using a Savant SpeedVac (Thermo Scientific, Waltham, MA, USA) [10,57,89]. Prior to proteomic profiling, the peptides were purified using an apparatus equipped with the appropriate column (C18 column, Thermo Scientific) and kept at −20 °C. Analysis was conducted on a nano ACQUITY UPLC-Xevo QTOF-MS (Waters, Manchester, UK), equipped with an HSS T3 column, in the positive electrospray ionization mode. Protein identification was achieved by processing mass spectra through Protein Lynx Global Server (PLGS v.2.5) against the *Gallus gallus* UniProt database. Subsequent bioinformatics analyses, including protein–protein interaction mapping and Gene Ontology classification (molecular function, cellular component, and biological process), were performed using the STRING platform based on FASTA sequences [10,57,89].

### 4.7. Characterization of Passion Fruit Seed Oil

#### 4.7.1. Se and Zn Determination

Approximately 0.9354 g of PFSO, weighed in triplicate, was subjected to acid digestion. Initially, 2 mL of nitric acid was added, and the sample was heated until partial digestion of the organic matter was achieved (extract with a cloudy appearance). Subsequently, 1 mL of hydrogen peroxide was added, and heating was maintained until the extract became transparent, indicating complete mineralization of the organic matter. After mineralization, the resulting acid extract was diluted to a final volume with ultrapure water (18.2 MΩ cm^−1^). The Se and Zn determinations in the acid extracts, from the PFSO samples processed in triplicate, followed the procedure described in item 4.5 [87,88,89].

#### 4.7.2. Volatile Compounds Analysis

Volatile profile characterization of the PFSO samples was conducted using Headspace Solid-Phase Microextraction (HS-SPME) coupled with Gas Chromatography–Mass Spectrometry (GC-MS), following the methodology outlined by Campos Oliveira et al. (2024) [90]. Briefly, 2.00 mL of raw sample was placed in a screw-cap vial and equilibrated at 90 °C for 60 min, with a 1-min agitation period prior to sampling. Volatiles were captured using a DVB/CAR/PDMS fiber (2 cm, 75 μm), which was then introduced into the GC injector at 230 °C (1:1 split ratio). Separation was performed on an Agilent 7890A GC coupled with an Agilent MSD 5975C mass spectrometer, utilizing an Agilent DB-FFAP capillary column (30 m × 250 μm × 0.25 μm), which consists of a nitroterephthalic acid-modified polyethylene glycol (PEG) stationary phase (Agilent Technologies, Santa Clara, CA, USA). Helium as the carrier gas (1 mL min^−1^). The thermal gradient started at 60 °C (2 min), rising to 80 °C at 3 °C min^−1^, then to 180 °C at 5 °C min^−1^, and finally reaching 230 °C at 7 °C min^−1^, where it remained for 3 min. Post-acquisition, data were transformed from .d to .cdf via GCMS ChemStation Translator 7890A GC (Santa Clara, CA, USA), and subsequently to .abf format for MS-DIAL processing. The software parameters included a 20-scan peak width, a 2500-amplitude threshold, and a 0.5 sigma window for deconvolution. Alignment used a 0.075 retention-time factor. Compound identification was performed putatively based on retention indices, mass accuracy, isotopic patterns, and MS/MS spectra, following the developer’s guidelines without standard confirmation.

### 4.8. Statistical Analysis

Analysis of 2D PAGE gel images was conducted using ImageMaster 2D Platinum software (v. 7.0, GE Healthcare), according to the methodology described by Lima et al. (2020) [57]. To assess significant differences in Se and Zn concentrations, Student’s *t*-tests and F-tests were performed using SAS software (v. 8) [57,89], with results expressed as the mean ± standard deviation (M ± SD). Furthermore, proteomic data were acquired on a nanoAcquity UPLC Xevo QT mass spectrometer and processed using Protein Lynx Global Server (PLGS) software (v. 2.5) [11,89].

## 5. Conclusions

The coordination of cellular homeostasis and energy metabolism (glycolysis and ATP synthesis)—mediated by crucial proteins such as HSPs, PKM, and ATP5F1B—is intrinsically dependent on the balance of micronutrients, particularly Se and Zn. Metalloproteomic data demonstrate that supplementation with PFSO in the laying hen diet represents more than a mere source of nutrients; it is a strategy that, through its antioxidant compounds, balances the absorption of Se and Zn. This facilitates the proper incorporation of these minerals into functional proteins, such as HSPs, which are key components of the *Se/Zn-binding protein* (*Se/Zn-BP*) network, participating in antioxidant protection and in the structural and catalytic modulation of core enzymes. This, in turn, ensures cellular resilience and liver health, thereby improving egg productivity and quality. However, further metalloproteomic studies employing strategies such as shotgun LC-MS/MS to analyze the expression of *Se/Zn-BPs* are crucial to accurately elucidate the complex network of interactions among bioactive compounds, minerals, and the liver proteome of laying hens.

## Figures and Tables

**Table 1:** Average Se and Zn concentrations in protein pellets from the liver tissue of laying hens exposed to thermoneutrality and heat stress, with and without PFSO inclusion in the diet.

Treatment Groups	Se Concentration (µg kg^−1^)	Zn Concentration (mg kg^−1^)
SC	324.1 ± 3.971 ^b^	28.43 ± 0.32 ^b^
TC	395.6 ± 4.543 ^c^	34.62 ± 0.38 ^c^
SPFSO	471.5 ± 4.838 ^a^	49.43 ± 0.55 ^a^
TPFSO	475.5 ± 4.973 ^a^	50.34 ± 0.57 ^a^
DOLT-4	8.168 ± 0.1454 *	114.5 ± 1.970

^a–c^ Means followed by different superscript letters in the same column are significantly different according to Tukey’s test (*p* < 0.05). Groups: SC—stress control without PFSO supplementation in the birds’ diet; TC—thermoneutral control without PFSO supplementation in the birds’ diet; SPFSO—heat stress with PFSO supplementation in the birds’ diet; TPFSO—thermoneutral with PFSO supplementation in the birds’ diet. DOLT-4—certified reference material, containing * Se (certified value in mg kg^−1^) at 8.300 ± 1.300 and Zn (certified value in mg kg^−1^) at 116.0 ± 6.000.

**Table 2:** Se and Zn concentrations in protein spots of liver tissue from laying hens in the experimental groups. TM—thermoneutral control, TPFSO—thermoneutral passion fruit seed oil, SPFSO—stress passion fruit seed oil, and SC (stress control).

	Se Concentration (mg kg^−1^)				Zn Concentration (mg kg^−1^)			
ID Spots	TPFSO	TC	SPFSO	SC	TPFSO	TC	SPFSP	SC
2	117.6 ± 1.24	91.44 ± 0.98	112.3 ± 1.21	36.11 ± 0.43	108.8 ± 1.16	85.12 ± 0.91	104.3 ± 1.14	33.53 ± 0.48
3	125.2 ± 1.31	88.13 ± 0.95	116.4 ± 1.21	36.66 ± 0.42	116.4 ± 1.29	82.05 ± 0.91	110.7 ± 1.13	34.67 ± 0.41
4	122.2 ± 1.29	85.12 ± 0.90	117.6 ± 1.23	31.12 ± 0.38	113.4 ± 1.22	80.24 ± 0.89	108.9 ± 1.14	29.64 ± 0.33
6	129.3 ± 1.36	86.93 ± 0.92	120.3 ± 1.26	33.35 ± 0.39	122.4 ± 1.27	82.35 ± 0.88	115.4 ± 1.22	31.22 ± 0.37
7	127.2 ± 1.34	89.16 ± 0.97	114.6 ± 1.21	41.53 ± 0.52	117.9 ± 1.31	86.16 ± 0.94	106.7 ± 1.14	38.14 ± 0.49
9	131.2 ± 1.39	90.23 ± 0.98	116.4 ± 1.21	37.44 ± 0.45	123.5 ± 1.28	88.13 ± 0.52	107.8 ± 1.15	35.12 ± 0.41
10	124.6 ± 1.31	87.73 ± 0.91	117.2 ± 1.22	34.41 ± 0.43	118.2 ± 1.25	85.52 ± 0.91	112.3 ± 1.18	31.72 ± 0.39
11	128.5 ± 1.33	84.13 ± 0.89	119.5 ± 1.26	36.93 ± 0.43	121.6 ± 1.29	83.45 ± 0.89	111.9 ± 1.20	33.92 ± 0.39
12	132.6 ± 1.36	86.12 ± 0.93	121.2 ± 1.28	33.21 ± 0.39	125.4 ± 1.31	83.25 ± 0.91	114.9 ± 1.19	31.44 ± 0.41
14	118.5 ± 1.24	84.12 ± 0.91	112.9 ± 1.23	32.65 ± 0.39	112.6 ± 1.19	81.93 ± 0.89	104.8 ± 1.17	31.12 ± 0.37
19	132.6 ± 1.36	86.34 ± 0.90	113.6 ± 1.20	36.94 ± 0.44	111.9 ± 1.19	82.73 ± 0.89	106.7 ± 1.17	34.13 ± 0.45

Results are expressed as the mean value ± standard deviation (n = 3, *p* < 0.05).

**Table 3:** Average concentrations of Se and Zn in PFSO.

Replicas	Se	Zn
C_1_	112.7 µg kg^−1^	17.22 mg kg^−1^
C_2_	113.1 µg kg^−1^	16.90 mg kg^−1^
C_3_	113.4 µg kg^−1^	17.24 mg kg^−1^
C_average_	113.1 ± 0.3512 µg kg^−1^	17.12 ± 0.1910 mg kg^−1^

Results are expressed as mean value ± standard deviation (n = 3, *p* < 0.05).

**Table 4:** Proteins identified by LC-MS/MS in protein spots associated with Se and Zn in the liver tissue of laying hens whose diet was supplemented with 0.9% passion fruit seed oil.

Spot ID	Protein Accession	Protein Description	Protein Score	Protein Cover (%)
2	Q90593	Endoplasmic reticulum chaperone BiP	16,770.69	73.93
	P19121	Albumin	2094.377	60.81
	O73885	Heat shock cognate 71 kDa protein	894.0323	19.2
	P08106	Heat shock 70 kDa protein	806.1342	24.61
	P11501	Heat shock protein HSP 90-alpha	272.8448	15.93
3	P19121	Albumin	23,501.01	81.14
	O73885	Heat shock cognate 71 kDa protein	12,071.11	60.99
	P08106	Heat shock 70 kDa protein	4705.15	38.01
	Q90593	Endoplasmic reticulum chaperone BiP	2289.996	41.41
	Q5ZM98	Stress-70 protein mitochondrial	810.4526	37.33
	P19121	Albumin	23,501.01	81.14
4	Q5ZL72	60 kDa heat shock protein mitochondrial	28,583.4	77.14
	P19121	Albumin	4066.385	69.59
	O73885	Heat shock cognate 71 kDa protein	901.8395	29.10
	P23228	Hydroxymethylglutaryl-CoA synthase Cytoplasmic	393.8779	25.67
6	Q5ZLC5	ATP synthase F(1) complex catalytic subunit beta mitochondrial	11,994.83	70.54
	P60706	Actin cytoplasmic 1	3044.344	71.73
	P09652	Tubulin beta-4 chain	512.5506	27.84
	Q5ZL72	60 kDa heat shock protein mitochondrial	434.258	38.74
7	P09102	Protein disulfide-isomerase	9240.172	68.93
	Q5ZLC5	ATP synthase F(1) complex catalytic subunit beta mitochondrial	2771.582	66.60
9	P60706	Actin cytoplasmic 1	25,819.53	89.87
10	Q5ZMQ2	Actin cytoplasmic 2	4799.331	72.80
	P07341	Fructose-bisphosphate aldolase B	426.1642	35.99
	P47826	Large ribosomal subunit protein uL10	300.3903	9.49
11	P60706	Actin cytoplasmic 1	2892.584	64.00
12	P60706	Actin cytoplasmic 1	2670.898	49.07
14	O57476	Hsp90 co-chaperone Cdc37	233.532	13.49
	Q5F4B1	Glycerol-3-phosphate phosphatase	2296.394	43.27
	P13127	F-actin-capping protein subunit alpha-1	1172.36	60.84
19	P00368	Glutamate dehydrogenase 1 mitochondrial (Fragment)	4594.764	39.96
	P51913	Alpha-enolase	1111.93	18.66
	P07322	Beta-enolase	1019.503	10.83
	P00548	Pyruvate kinase PKM	891.7976	35.09
	Q5F3T9	UDP-glucose 6-dehydrogenase	388.1268	31.58
	P27463	Aldehyde dehydrogenase 1A1	305.6971	18.47

## Data Availability

The datasets generated and analyzed during the current study are available in the Supplementary Materials. Further inquiries can be directed to the corresponding author.

## Supplementary Materials

The following supporting information can be downloaded at: https://www.mdpi.com/article/10.3390/ijms27041646/s1.
