# Supplementary material for: Passion Fruit Seed Oil Modulates the Hepatic Metalloproteomic Profile of Selenium and Zinc in Laying Hens Under Heat Stress
Source: Int J Mol Sci. 2026 Feb 8;27(4):1646. doi: 10.3390/ijms27041646 (PMC12941272; doi:10.3390/ijms27041646)
Supplement: Supplementary file 1 [file ijms-27-01646-s001.zip › ijms-4083458-supplementary.pdf]

Supplementary Material

Table S1 - Ingredients and calculated chemical composition of the experimental diets.

Ingredients (%)	Control	PFOS 0,9%
Corn	58.39	58.39
Soybean meal	28.90	28.90
Soybean oil	2.000	1.040
Passion fruit seed oil (PFSO)	0.000	0.9000
Fine limestone	1.600	1.600
Coarse limestone	5.400	5.400
Dicalcium phosphate	0.4500	0.450
Salt	0.08000	0.0800
DL-Methionine (99%)	0.08000	0.0800
Laying hen premix	3.000	3.000
Kaolin – Inert	0.1000	0.1600
Total (kg)	100.0	100.0
Apparent metabolizable energy (kcal/kg)	2,833	2.833
Crude protein (%)	17.50	17.50
Calcium (%)	3.690	3.6900
Available phosphorus (%)	0.4200	0.4200
Sodium (%)	0.1700	0.1700
Digestible lysine (%)	0.8400	0.8400
Digestible methionine (%)	0.3600	0.3600
Digestible methionine + cystine (%)	0.6300	0.6300
Digestible tryptophan (%)	0.2000	0.2000
Digestible threonine (%)	0.5900	05900

Passion fruit seed oil; Premix for egg production (guaranteed levels per kg of product): Folic acid (min.): 15 mg/kg. Pantothenic acid (min.): 350 mg/kg. Biotin (min.): 1.5 mg/kg. Calcium (min.): 250 g/kg. Calcium (max.): 270 g/kg. Copper: 225 mg/kg. Choline (min.): 6,000 mg/kg. Iron: 1,500 mg/kg. Phytase: 16.67 ftu/g. Fluorine: 460 mg/kg. Phosphorus: 49 g/kg. Iodine (min.): 20 mg/kg. Manganese (min.): 2,800 mg/kg. Methionine: 23.43 g/kg. Selenium: 6 mg/kg. Sodium (min): 45 g/kg. Niacin (min): 800 mg/kg. Vitamin A (min): 233,000 IU/kg. Vitamin B1 (min): 35 mg/kg. Vitamin B12 (min): 350 mcg/kg. Vitamin B2 (min): 100 mg/kg. Vitamin B6 (min): 100 mg/kg. Vitamin D3 (min): 80,000 IU/kg. Vitamin E (min): 333,333 IU/kg. Vitamin K3 (min): 60 mg/kg. Zinc (min): 2,000 mg/kg.

Table S2. Se and Zn determinations in liver tissue samples from laying hens exposed in a thermoneutral chamber, with the inclusion of PFSO in the diet.

Treatments	Se Concentration ($\mu g\ kg^{-1}$)	Zn Concentration ($\mu g\ kg^{-1}$)
TM2	585.2±6.681	64.40±0.7624
TM4	586.7±6.739	66.04±0.7941
TM9	587.3±6.785	65.31±0.8121
TM12	587.4±6.670	64.83±0.7213
TM14	587.6±6.658	65.80±0.7414
TM16	585.8±6.727	65.90±0.8342

There was no statistically significant difference in the concentrations of Se and Zn determined among the six treatments ($p > 0.05$).

Table S3. Se and Zn determinations in liver tissue samples from laying hens exposed to the thermoneutral chamber (control), without the inclusion of PFSO in the diet.

Treatments	Se Concentration ($\mu g\ kg^{-1}$)	Zn Concentration ($\mu g\ kg^{-1}$)
TC3	533.4±5.576	44.73±0.5431
TC5	535.3±6.050	46.90±0.5724
TC7	535.7±5.753	45.04±0.5312
TC10	533.5±5.391	44.72±0.4822
TC15	534.4±5.412	45.70±0.4941
TC17	533.6±6.010	46.10±05733

There was no statistically significant difference in the concentrations of Se and Zn determined among the six treatments ($p > 0.05$).

Table S4. Se and Zn Determination in liver tissue samples from laying hens exposed to a heat stress chamber with the inclusion of PFSO in the diet.

Treatments	Se Concentration ($\mu g\ kg^{-1}$)	Zn Concentration ($\mu g\ kg^{-1}$)
ME19	578.3±6.473	63.43±0.7224
ME24	576.2±6.345	65.51±0.7712
ME26	578.1±6.493	64.50±0.7652
ME29	576.6±6.347	64.30±0.7144
ME33	577.4±6.364	63.34±0.7533
ME34	577.8±6.351	64.52±0.7422

There was no statistically significant difference in the concentrations of Se and Zn determined among the six treatments ($p > 0.05$).

Table S5. Se and Zn determination in liver tissue samples from laying hens exposed to a heat stress chamber, without the inclusion of PFSO in the diet.

Treatments	Se Concentration ($\mu g\ kg^{-1}$)	Zn Concentration ($\mu g\ kg^{-1}$)
	Tecido hepático	
CE20	462.5±0,5213	37.28±0.4313
CE22	461.6±0,5134	36.50±0.3908
CE27	463.2±0,5522	38.64±0.4522
CE28	461.8±0,5617	36.20±0.4131
CE32	463.3±0,5412	36.46±0.4214
CE36	462.8±0,5113	37.22±0.4033

There was no statistically significant difference in the concentrations of Se and Zn determined among the six treatments ($p > 0.05$).

Table S6. Raw data from the proteins identification analysis by LC-MS/MS in the protein spots associated with Se and Zn in the liver tissue of laying hens fed diets supplemented with 0.9% PFSO.

Spot ID	Protein Accession	Protein Description	Protein score	protein seqCover(%)	pI/MM (Da) Teórica	pI/MM (Da) Experimental
2	Q90593	Endoplasmic reticulum chaperone BiP	16770,69	73,93	5,12/72,019	5,35/76,990
	P19121	Albumin	2094,377	60,81	5,51/69,918	
	O73885	Heat shock cognate 71 kDa protein	894,0323	19,2	5,46/70,827	
	P08106	Heat shock 70 kDa protein	806,1342	24,61	5,52/69,751	
	P11501	Heat shock protein HSP 90-alpha	272,8448	15,93	5,00/84,059	
3	P19121	Albumin	23501,01	81,14	5,51/69,918	5,75/72,259
	O73885	Heat shock cognate 71 kDa protein	12071,11	60,99	5,46/70,827	
	P08106	Heat shock 70 kDa protein	4705,15	38,01	5,52/69,751	
	Q90593	Endoplasmic reticulum chaperone BiP	2289,996	41,41	5,12/72,019	
	Q5ZM98	Stress-70 protein mitochondrial	810,4526	37,33	6,09/73,192	
	P19121	Albumin	23501,01	81,14	5,51/69,918	
	Q5ZL72	60 kDa heat shock protein mitochondrial	28583,4	77,14	5,72/60,973	5,73/60,523
	P19121	Albumin	4066,385	69,59	5,51/69,918	

4	O73885	Heat shock cognate 71 kDa protein	901,8395	29,1	5,46/70,827	
	P23228	Hydroxymethylglutaryl-CoA synthase cytoplasmic	393,8779	25,67	5,41/57,559	
6	Q5ZLC5	ATP synthase F (1) complex catalytic subunit beta mitochondrial	11994,83	70,54	5,59/56,628	5,31/50,204
	P60706	Actin cytoplasmic 1	3044,344	71,73	5,29/41,737	
	P09652	Tubulin beta-4 chain	512,5506	27,84	4,86/50,421	
	Q5ZL72	60 kDa heat shock protein mitochondrial	434,258	38,74	5,72/60,973	
7	P09102	Protein disulfide-isomerase	9240,172	68,93	4,69/57,410	4,87/57,563
	Q5ZLC5	ATP synthase F(1) complex catalytic subunit beta mitochondrial	2771,582	66,6	5,59/56,628	
9	P60706	Actin cytoplasmic 1	25819,53	89,87	5,29/41,737	5,57/41,672
10	Q5ZMQ2	Actin cytoplasmic 2	4799,331	72,8	5,31/41,793	6,13/36,742
	P07341	Fructose-bisphosphate aldolase B	426,1642	35,99	8,81/39,296	
	Q9I923	Regucalcin	381,3167	21,07	5,77/33,230	
	P47826	Large ribosomal subunit protein uL10	300,3903	9,49	5,71/34,286	
11	Q9I923	Regucalcin	11270,92	72,58	5,77/33,230	6,12/33,664
	P60706	Actin cytoplasmic 1	2892,584	64	5,29/41,737	
	P17153	Annexin A5	8340,756	81	5,59/36,198	

12	P60706	Actin cytoplasmic 1	2670,898	49,07	5,29/41,737	5,98/32,120
	Q9I923	Regucalcin	631,6088	42,14	5,77/33,230	
	Q45KJ4	Protein lin-28 homolog B	228,4286	26,4	8,94/27,398	
14	P60706	Actin cytoplasmic 1	7737,45	57,87	5,29/41,737	5,86/34,611
	Q9I923	Regucalcin	3141,335	66,22	5,77/33,230	
	Q5F4B1	Glycerol-3-phosphate phosphatase	2296,394	43,27	5,51/32,996	
	P17153	Annexin A5	1682,657	44,55	5,59/36,198	
	Q5ZML3	Serine/arginine-rich splicing factor 1	1622,281	38,13	6,46/28,060	
	P13127	F-actin-capping protein subunit alpha-1	1172,36	60,84	5,43/32,960	
	P12275	Arylamine N-acetyltransferase liver isozyme	945,7117	27,18	5,56/32,915	
	P62207	Serine/threonine-protein phosphatase PP1-beta catalytic subunit	857,9753	15,29	5,84/37,187	
	Q5ZLY5	Pleckstrin homology domain-containing family F member 2	266,3604	17,27	8,41/27,715	
	Q05917	Homeobox protein engrailed-2	263,67	13,49	9,95/30,507	
	Q90XD2	Agmatinase mitochondrial	249,4255	19,12	5,99/36,488	
	O57476	Hsp90 co-chaperone Cdc37	233,532	13,49	5,24/45,608	
	P35001	Neuromodulin	190,3513	36,59	4,51/25,631	
	Q05437	Paired mesoderm homeobox protein 1	178,5257	3,67	9,48/27,201	

19	P00368	Glutamate dehydrogenase 1 mitochondrial (Fragment)	4594,764	39,96	8,48/55,712	7,52/53,757
	P51913	Alpha-enolase	1111,93	18,66	6,17/47,305	
	P07322	Beta-enolase	1019,503	10,83	7,28/47,196	
	P00548	Pyruvate kinase PKM	891,7976	35,09	7,29/58,015	
	Q5F3T9	UDP-glucose 6-dehydrogenase	388,1268	31,58	6,99/55,064	
	P27463	Aldehyde dehydrogenase 1A1	305,6971	18,47	7,49/55,809	

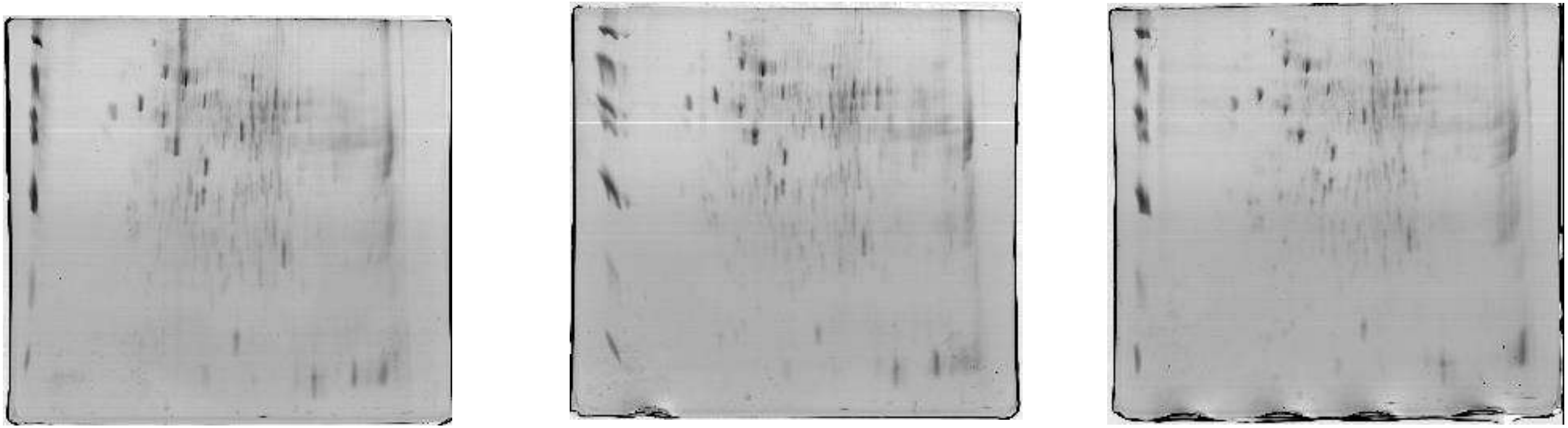

Fig. S1 - Crude replicas of 2D PAGE gels from liver proteome fractionation of laying hens fed a diet supplemented with 0.90% PFSO (**Stress Control - SC)**

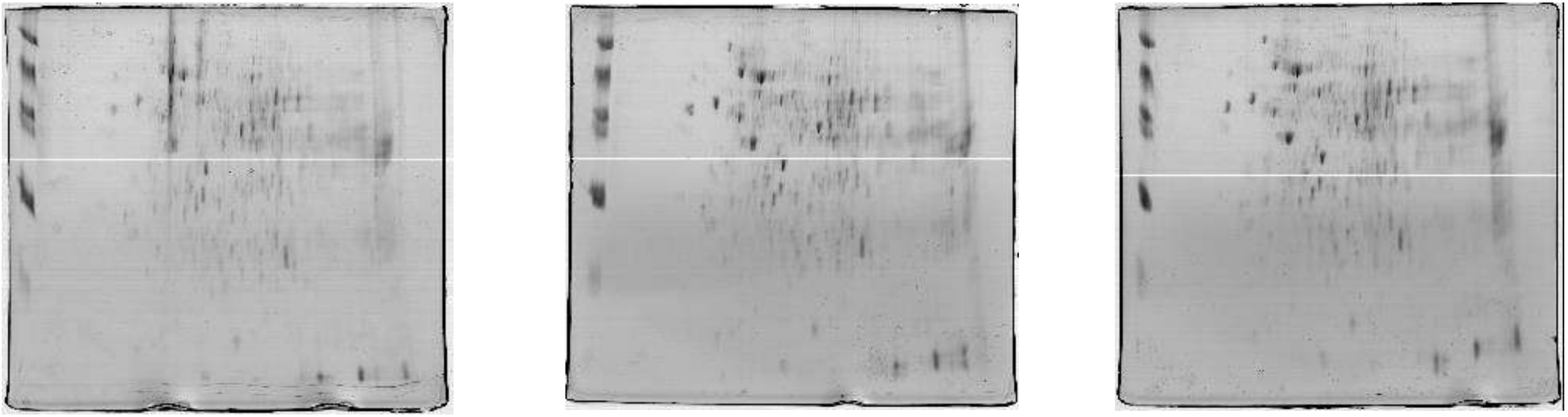

Fig. S2 - Crude replicas of 2D PAGE gels from liver proteome fractionation of laying hens fed a diet supplemented with 0.90% PFSO (**Stress PFSO - SPFSO)**

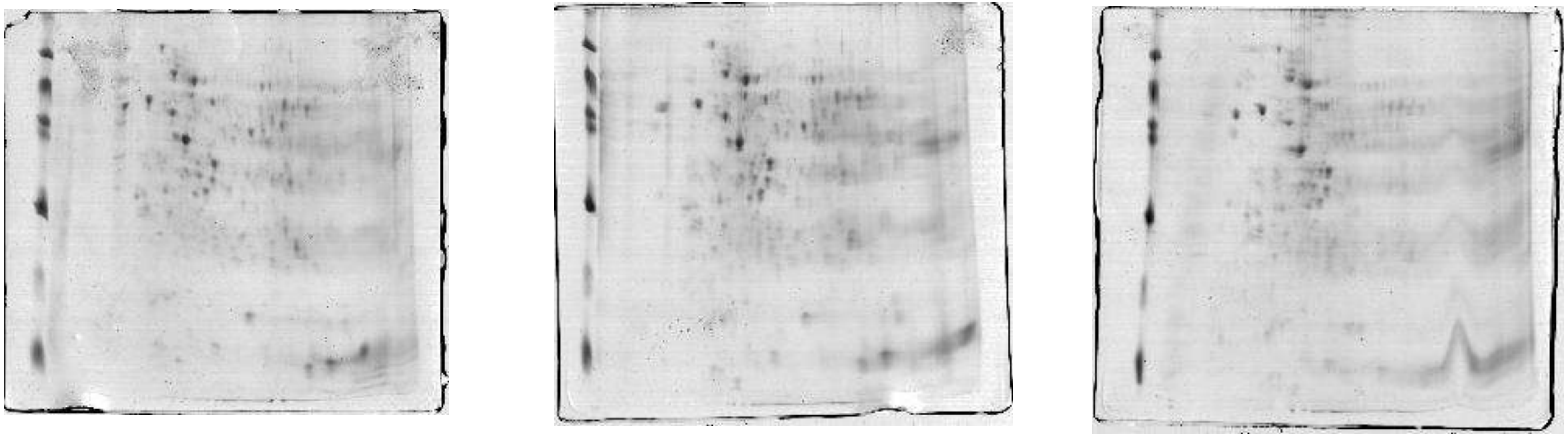

Fig. S3 - Crude replicas of 2D PAGE gels from liver proteome fractionation of laying hens fed a diet supplemented with 0.90% PFSO (**Thermoneutral Control – TC)**

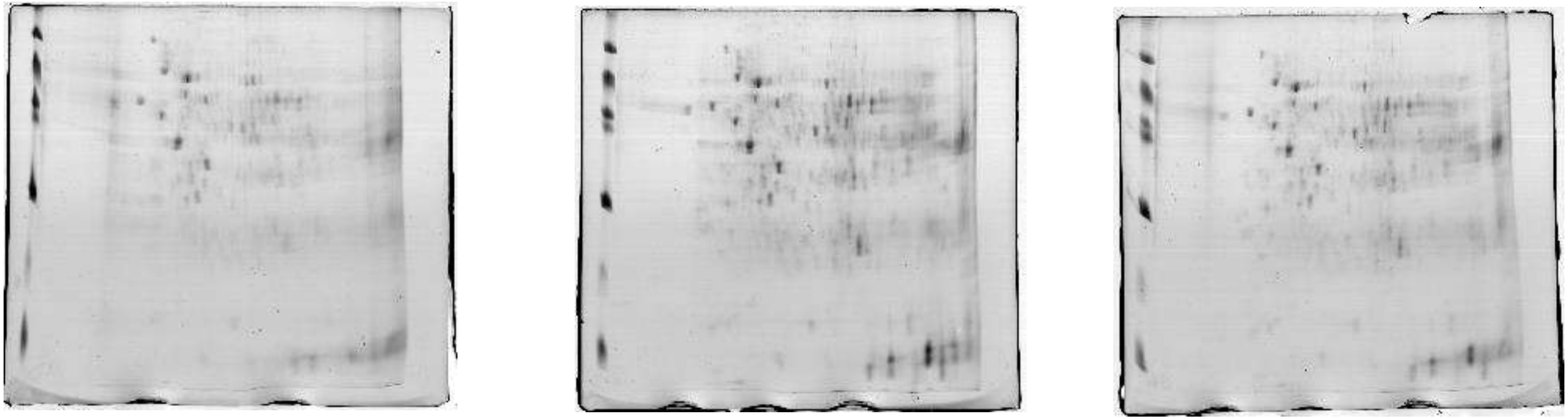

Fig. S4 - Crude replicas of 2D PAGE gels from liver proteome fractionation of laying hens fed a diet supplemented with 0.90% PFSO (Thermoneutral PFSO – TPFSO)

Raw Data - HS-SPME-LC-MS

Library Search Report

Data Path : C:\msdchem\1\data\2025\Docentes\Sussulini\Romani\
Data File : 300625_O2.D
Acq On : 30 Jun 2025 17:02
Operator :
Sample :
Misc :
ALS Vial : 2 Sample Multiplier: 1

Search Libraries: C:\Database\NIST08.L Minimum Quality: 0

Unknown Spectrum: Apex
Integration Events: ChemStation Integrator - autoint1.e

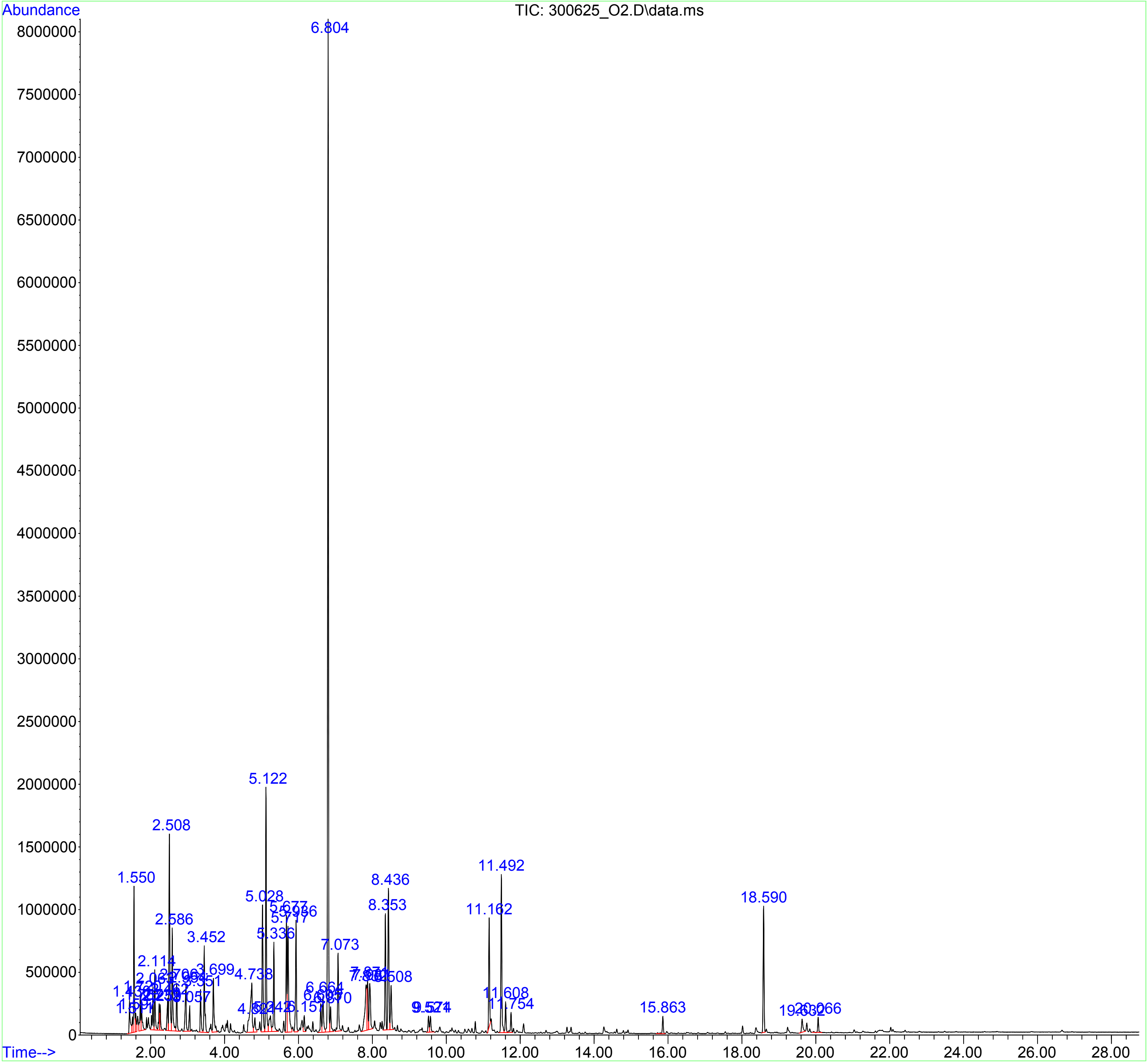

Library Search Report - ChemStation Integrator

Unknown Spectrum based on Apex

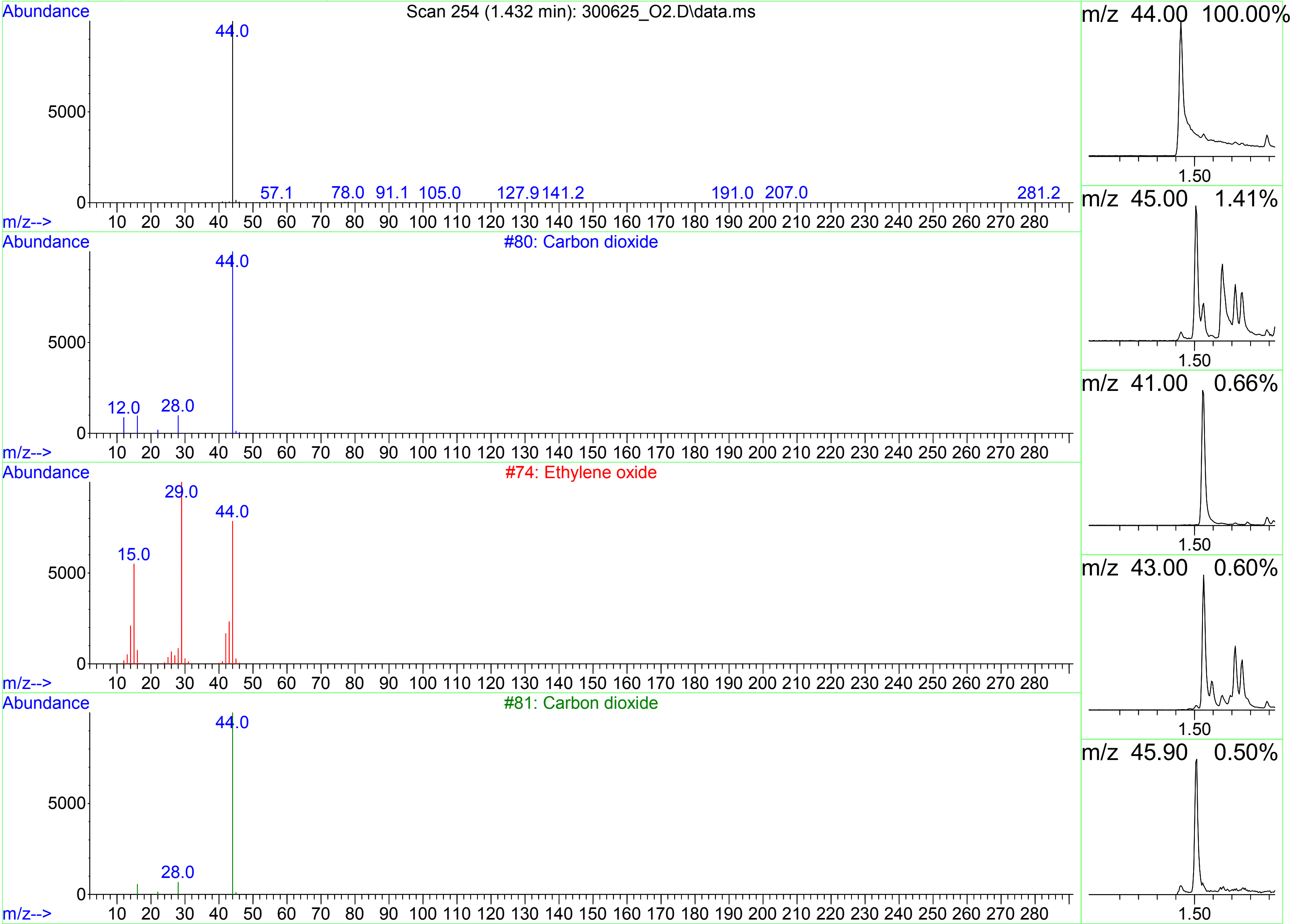

Data File: C:\msdchem\1\data\2025\Docentes\Sussulini\Romani\300625_O2.D
Sample :

Peak Number: 1 at 1.432 min Area: 5285471 Area % 0.92

The 3 best hits from each library.	Ref#	CAS#	Qual
C:\Database\NIST08.L			
1 Carbon dioxide	80	000124-38-9	4
2 Ethylene oxide	74	000075-21-8	3
3 Carbon dioxide	81	000124-38-9	3

Unknown Spectrum based on Apex

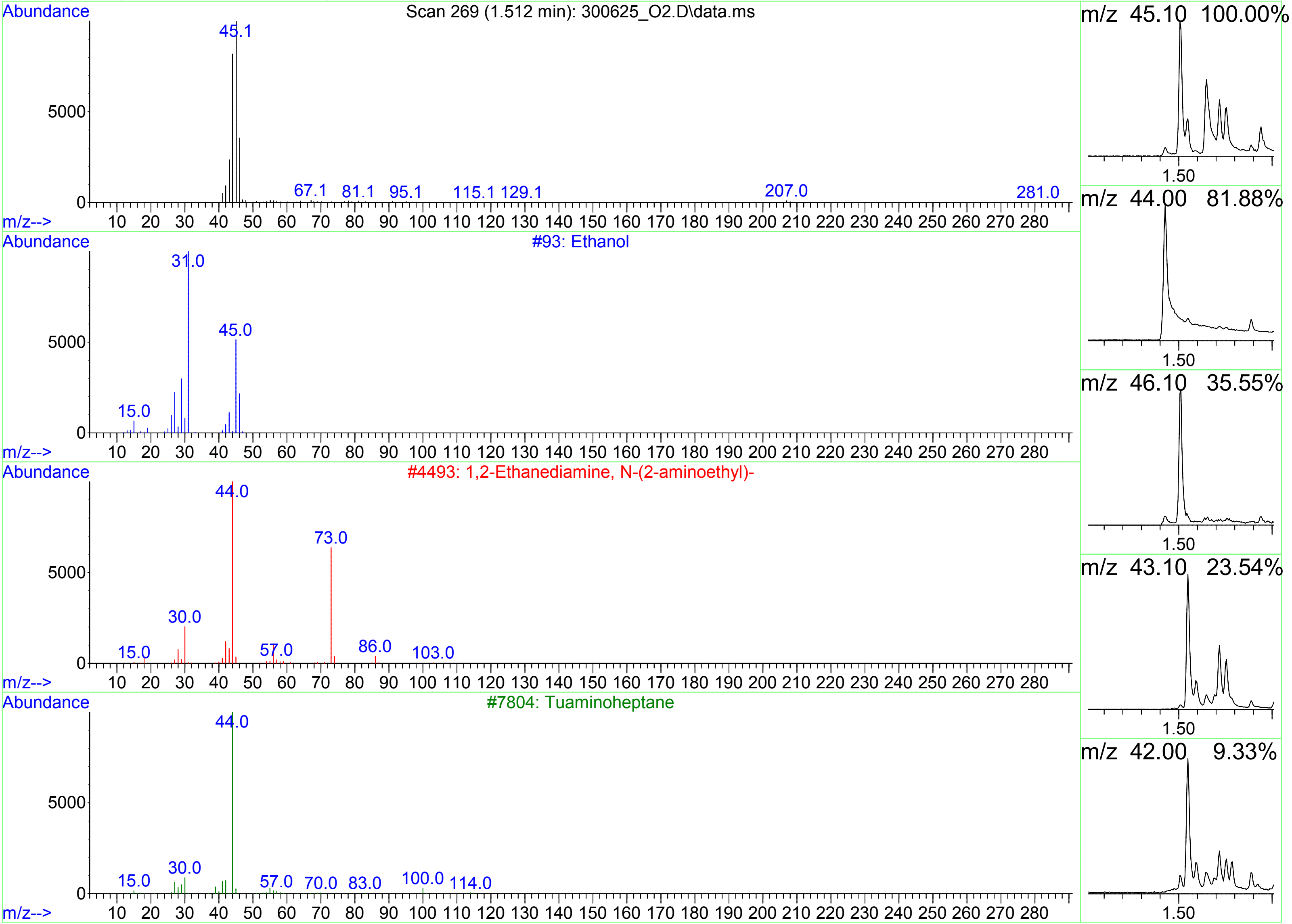

Data File: C:\msdchem\1\data\2025\Docentes\Sussulini\Romani\300625_O2.D
Sample :

Peak Number: 2 at 1.512 min Area: 1946992 Area % 0.34

The 3 best hits from each library. Ref\# CAS\# Qual

C:\Database\NIST08.L

1	Ethanol	93	000064-17-5	37
2	1,2-Ethanediamine, N-(2-aminoeth...	4493	000111-40-0	9
3	Tuaminoheptane	7804	000123-82-0	9

Unknown Spectrum based on Apex

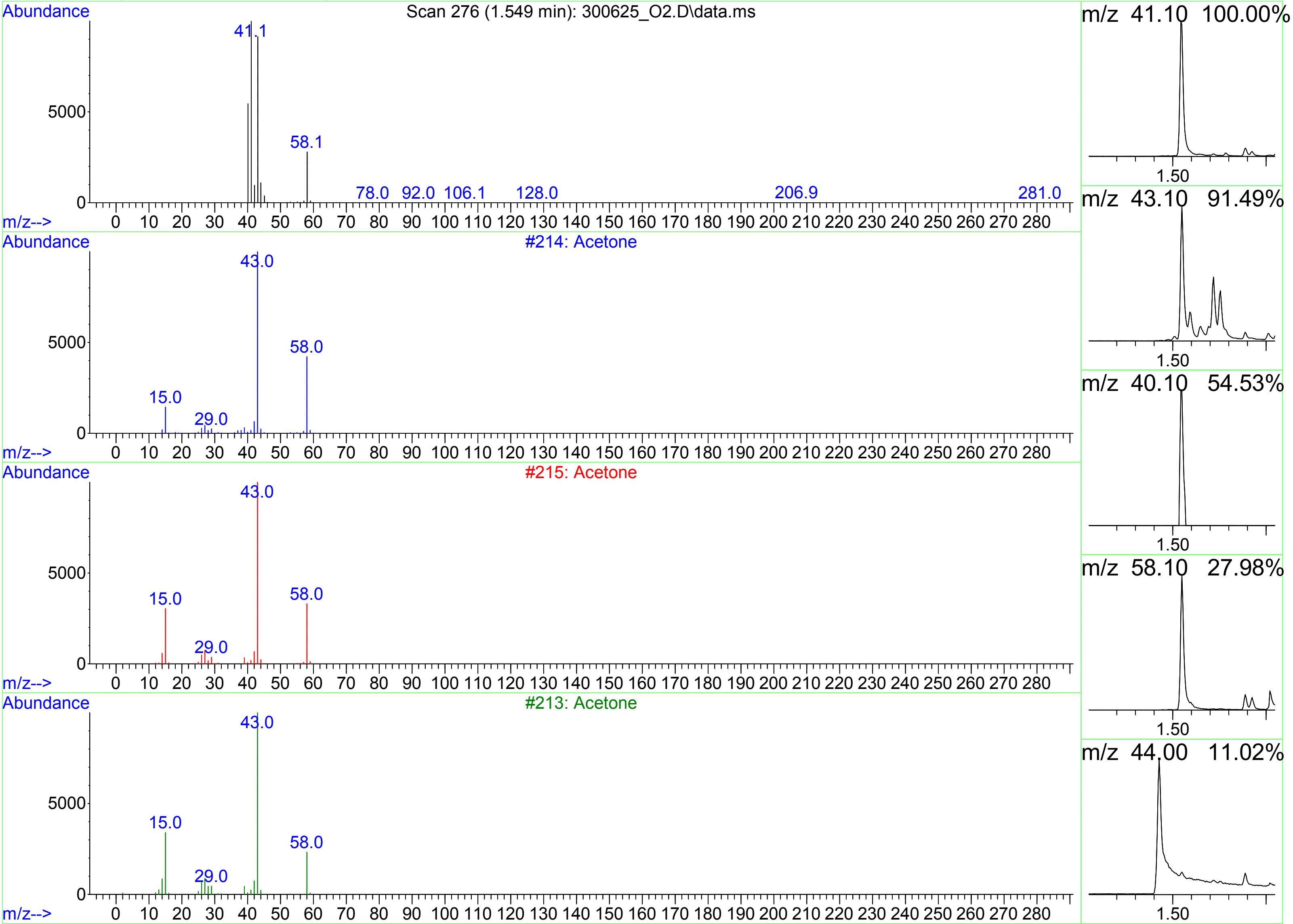

Data File: C:\msdchem\1\data\2025\Docentes\Sussulini\Romani\300625_O2.D
Sample :

Peak Number: 3 at 1.549 min Area: 15435619 Area % 2.70

The 3 best hits from each library.	Ref#	CAS#	Qual
C:\Database\NIST08.L			
1 Acetone	214	000067-64-1	37
2 Acetone	215	000067-64-1	9
3 Acetone	213	000067-64-1	7

Unknown Spectrum based on Apex

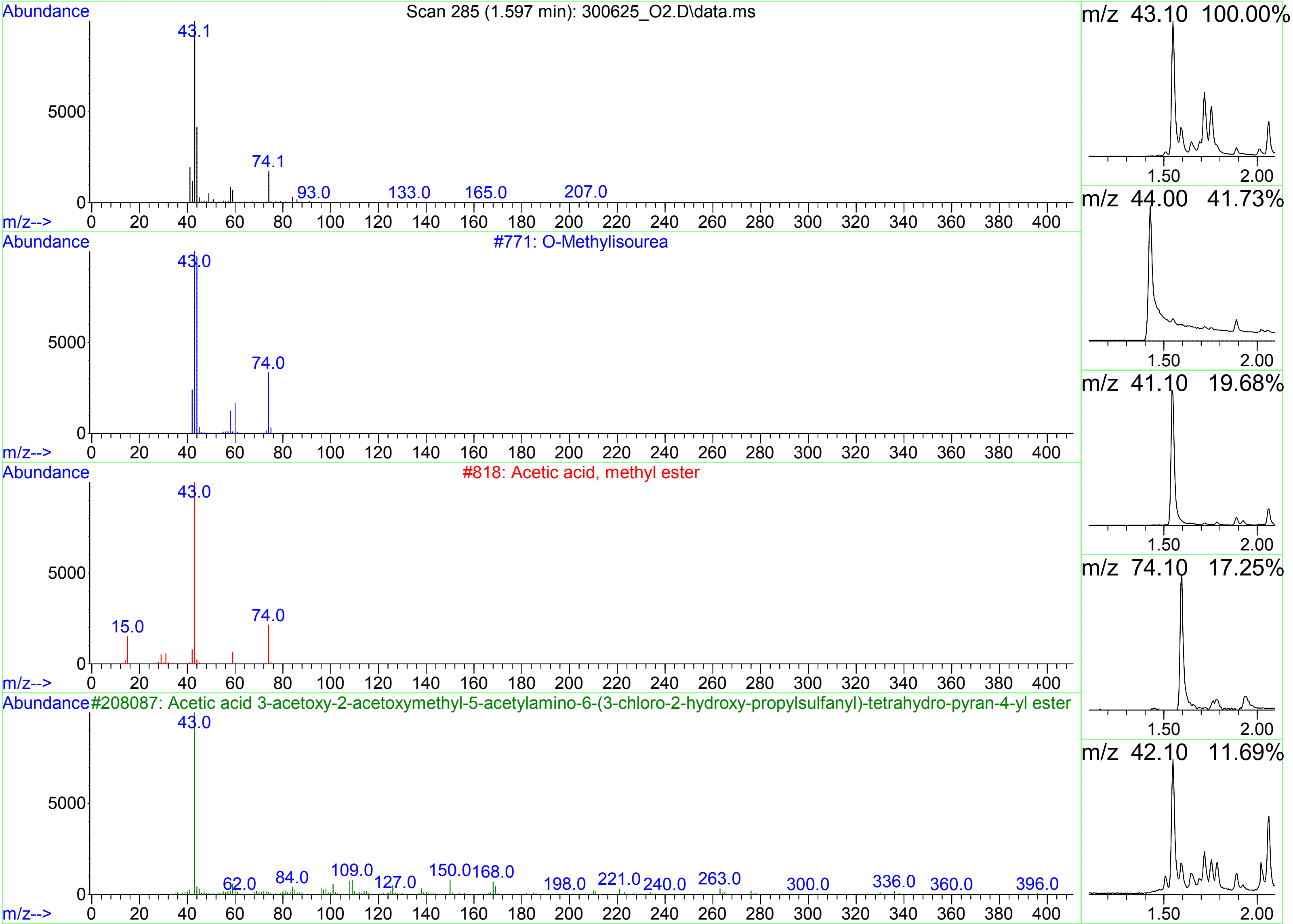

Data File: C:\msdchem\1\data\2025\Docentes\Sussulini\Romani\300625_O2.D
Sample :

Peak Number: 4 at 1.597 min Area: 2782710 Area % 0.49

The 3 best hits from each library.	Ref\#	CAS\#	Qual
C:\Database\NIST08.L			
1 O-Methylisourea	771	002440-60-0	53
2 Acetic acid, methyl ester	818	000079-20-9	9
3 Acetic acid 3-acetoxy-2-acetoxym...	208087	1000301-00-1	9

Unknown Spectrum based on Apex

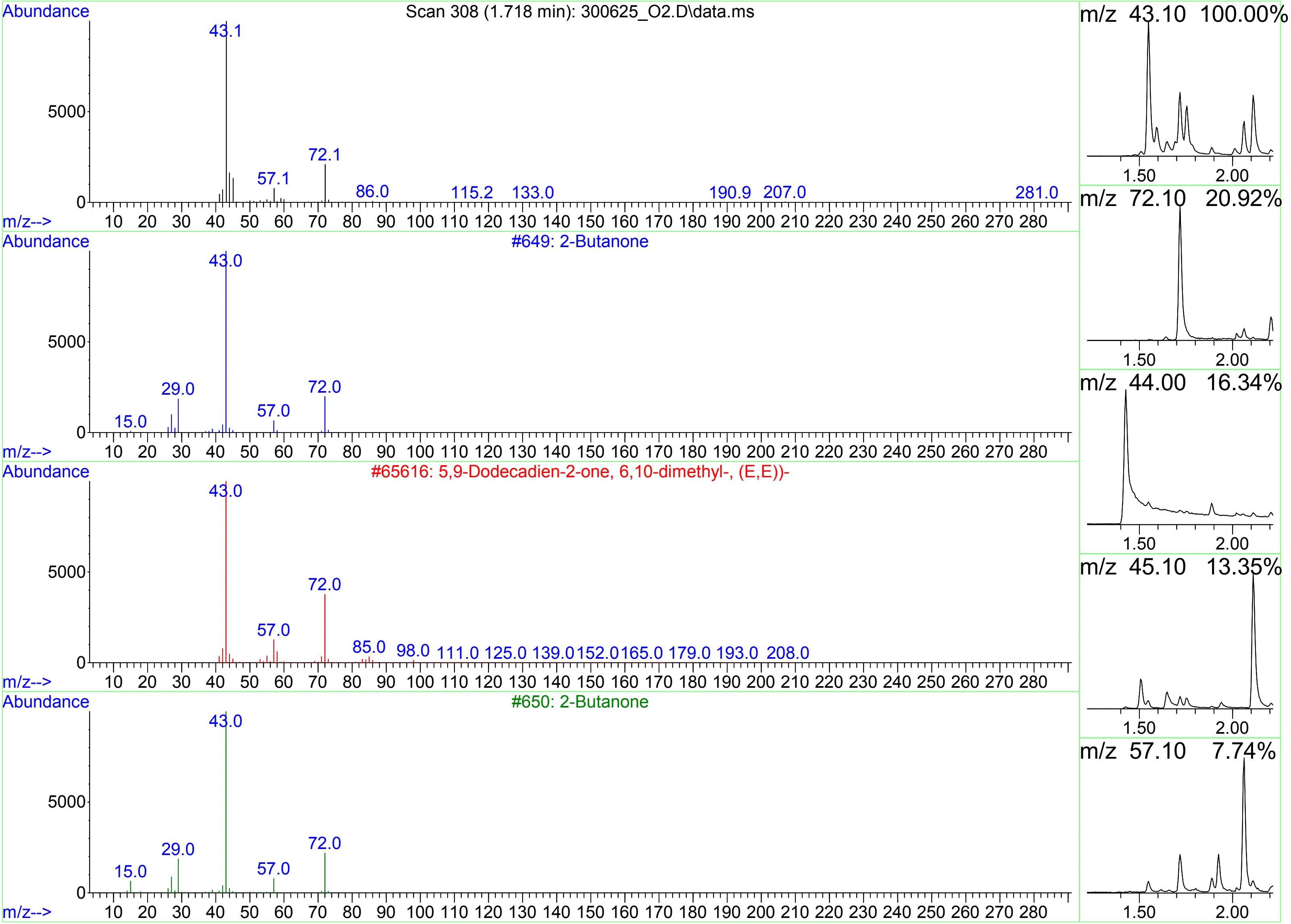

Data File: C:\msdchem\1\data\2025\Docentes\Sussulini\Romani\300625_O2.D
Sample :

Peak Number: 5 at 1.718 min Area: 4468445 Area % 0.78

The 3 best hits from each library.	Ref#	CAS#	Qual
C:\Database\NIST08.L			
1 2-Butanone	649	000078-93-3	50
2 5,9-Dodecadien-2-one, 6,10-dimet...	65616	1000132-10-9	42
3 2-Butanone	650	000078-93-3	40

Unknown Spectrum based on Apex

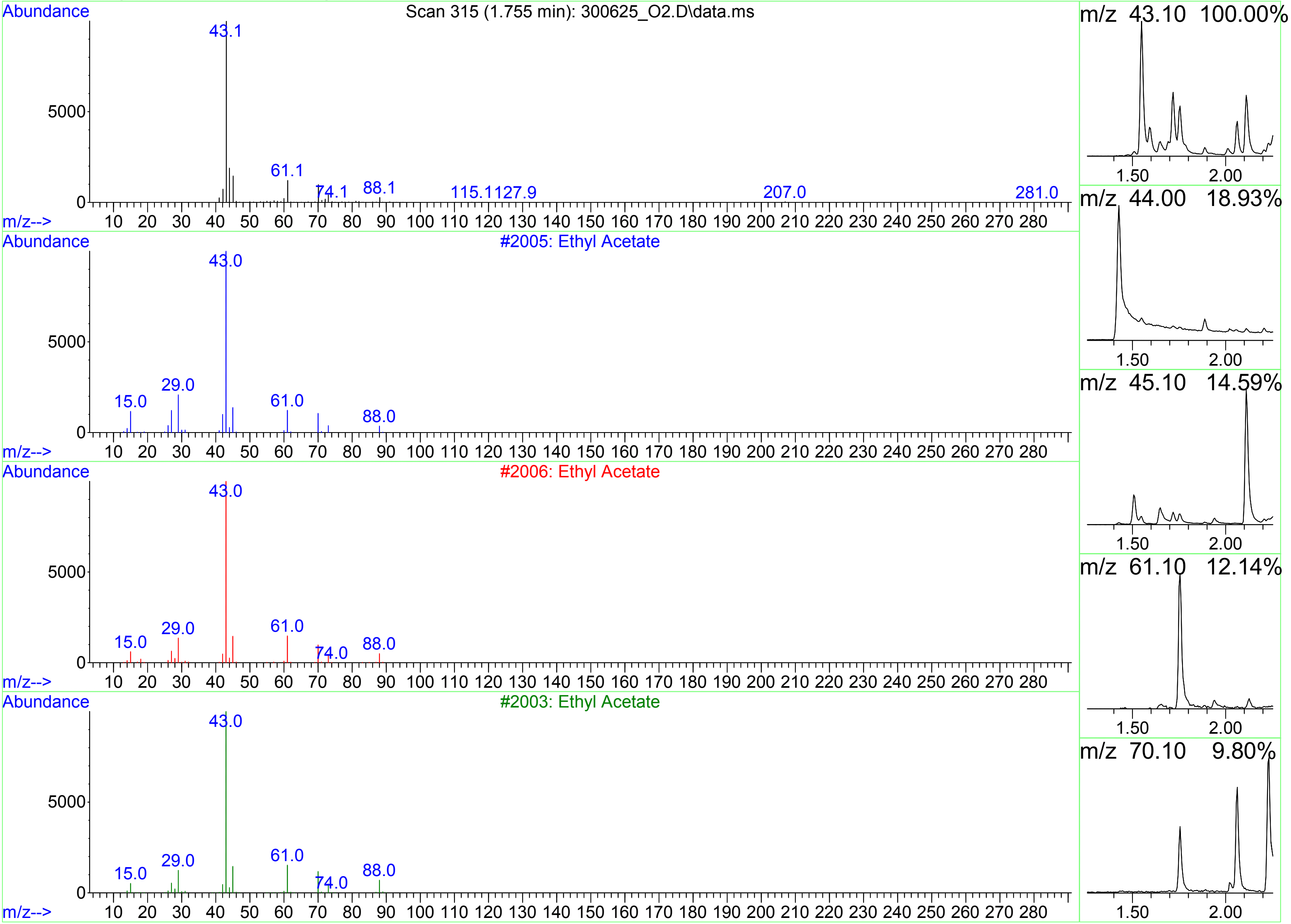

Data File: C:\msdchem\1\data\2025\Docentes\Sussulini\Romani\300625_O2.D
Sample :

Peak Number: 6 at 1.755 min Area: 3999067 Area % 0.70

The 3 best hits from each library. Ref\# CAS\# Qual

C:\Database\NIST08.L

1	Ethyl Acetate	2005	000141-78-6	72
2	Ethyl Acetate	2006	000141-78-6	50
3	Ethyl Acetate	2003	000141-78-6	50

Unknown Spectrum based on Apex

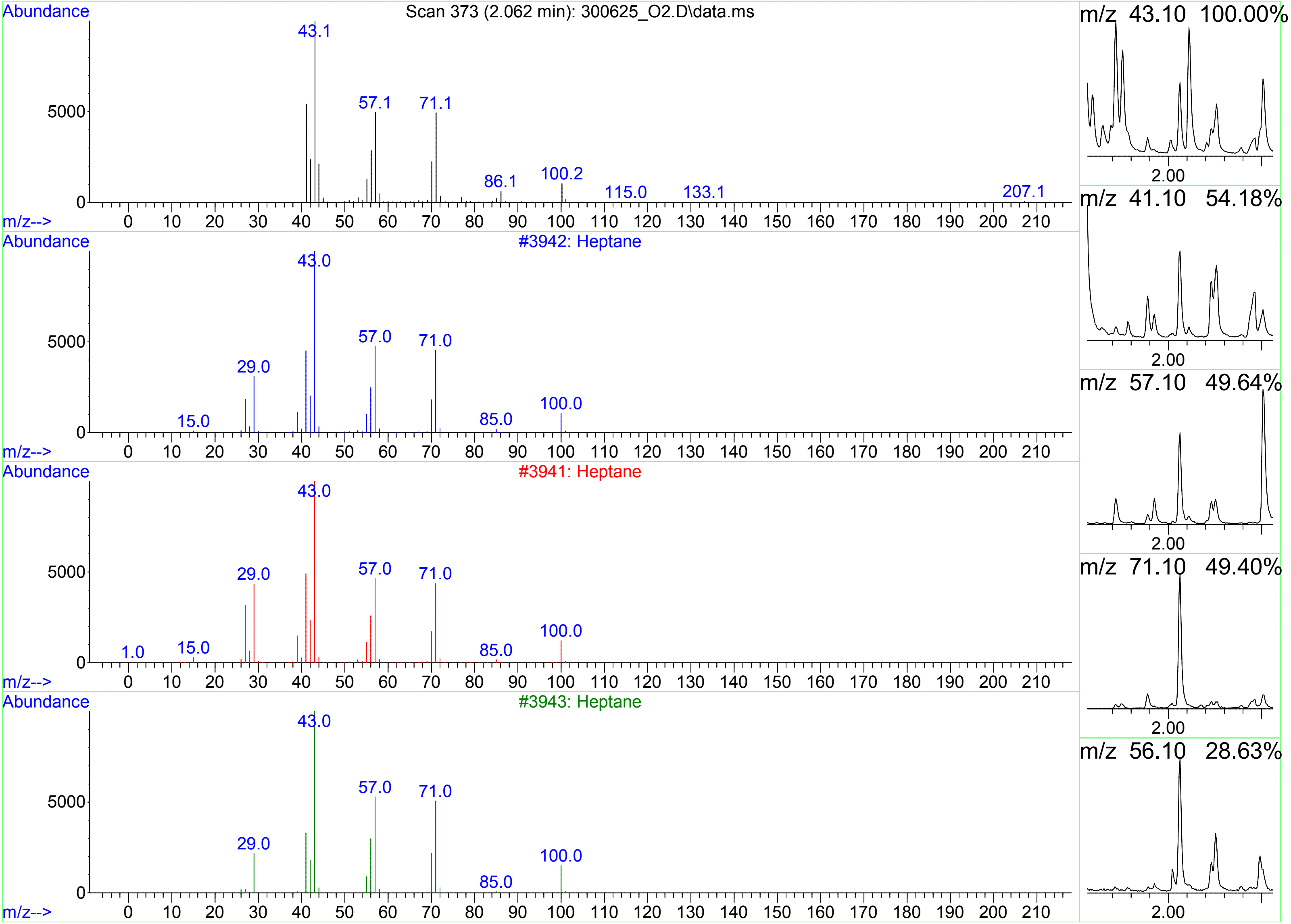

Data File: C:\msdchem\1\data\2025\Docentes\Sussulini\Romani\300625_O2.D
Sample :

Peak Number: 7 at 2.062 min Area: 5533060 Area % 0.97

The 3 best hits from each library.	Ref#	CAS#	Qual
C:\Database\NIST08.L			
1 Heptane	3942	000142-82-5	87
2 Heptane	3941	000142-82-5	76
3 Heptane	3943	000142-82-5	72

Library Search Report - ChemStation Integrator

Unknown Spectrum based on Apex

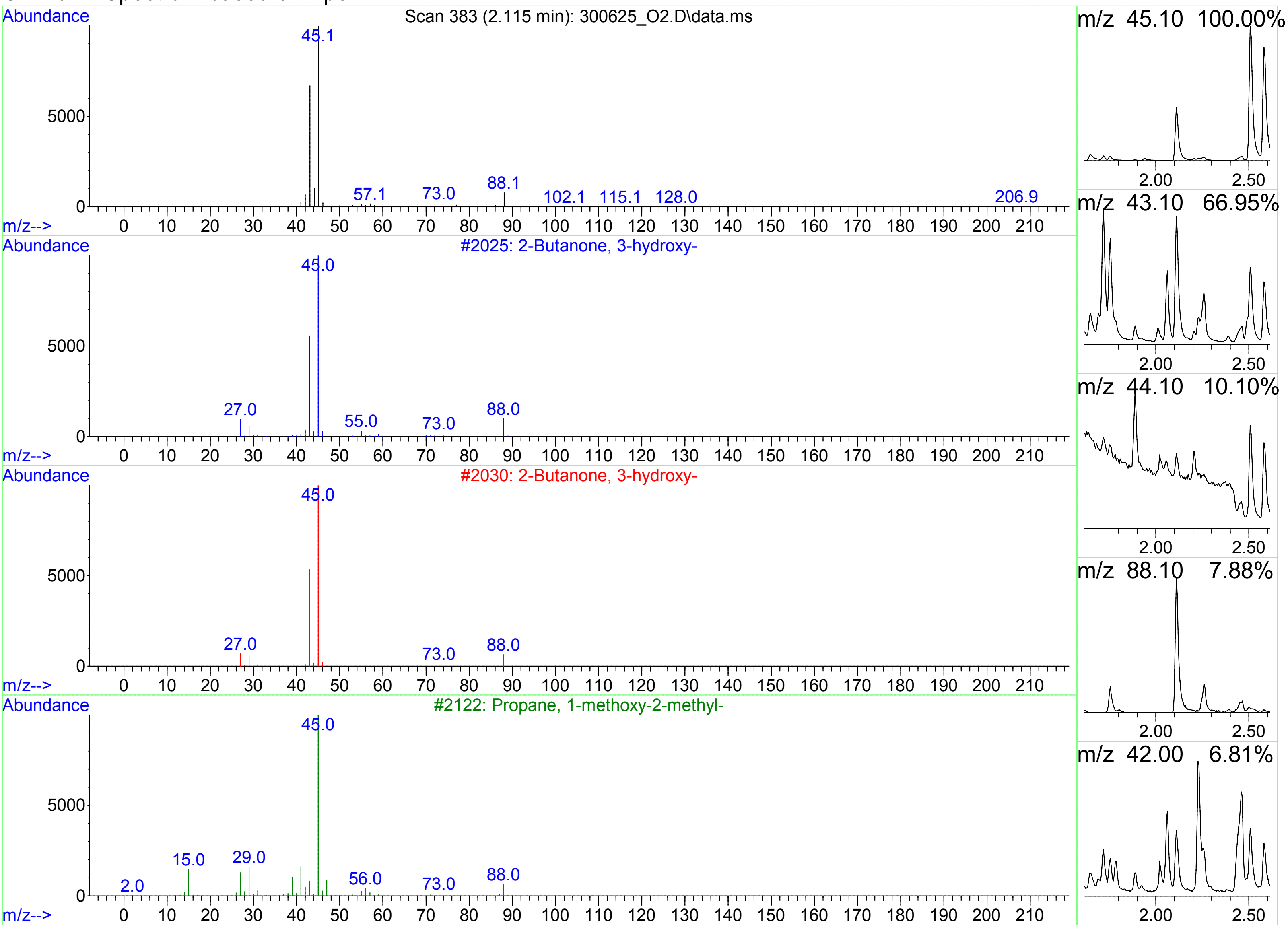

Data File: C:\msdchem\1\data\2025\Docentes\Sussulini\Romani\300625_O2.D
Sample :

Peak Number: 8 at 2.115 min Area: 6604945 Area % 1.16

The 3 best hits from each library. Ref# CAS# Qual

C:\Database\NIST08.L

		Ref#	CAS#	Qual
1	2-Butanone, 3-hydroxy-	2025	000513-86-0	72
2	2-Butanone, 3-hydroxy-	2030	000513-86-0	56
3	Propane, 1-methoxy-2-methyl-	2122	000625-44-5	9

Unknown Spectrum based on Apex

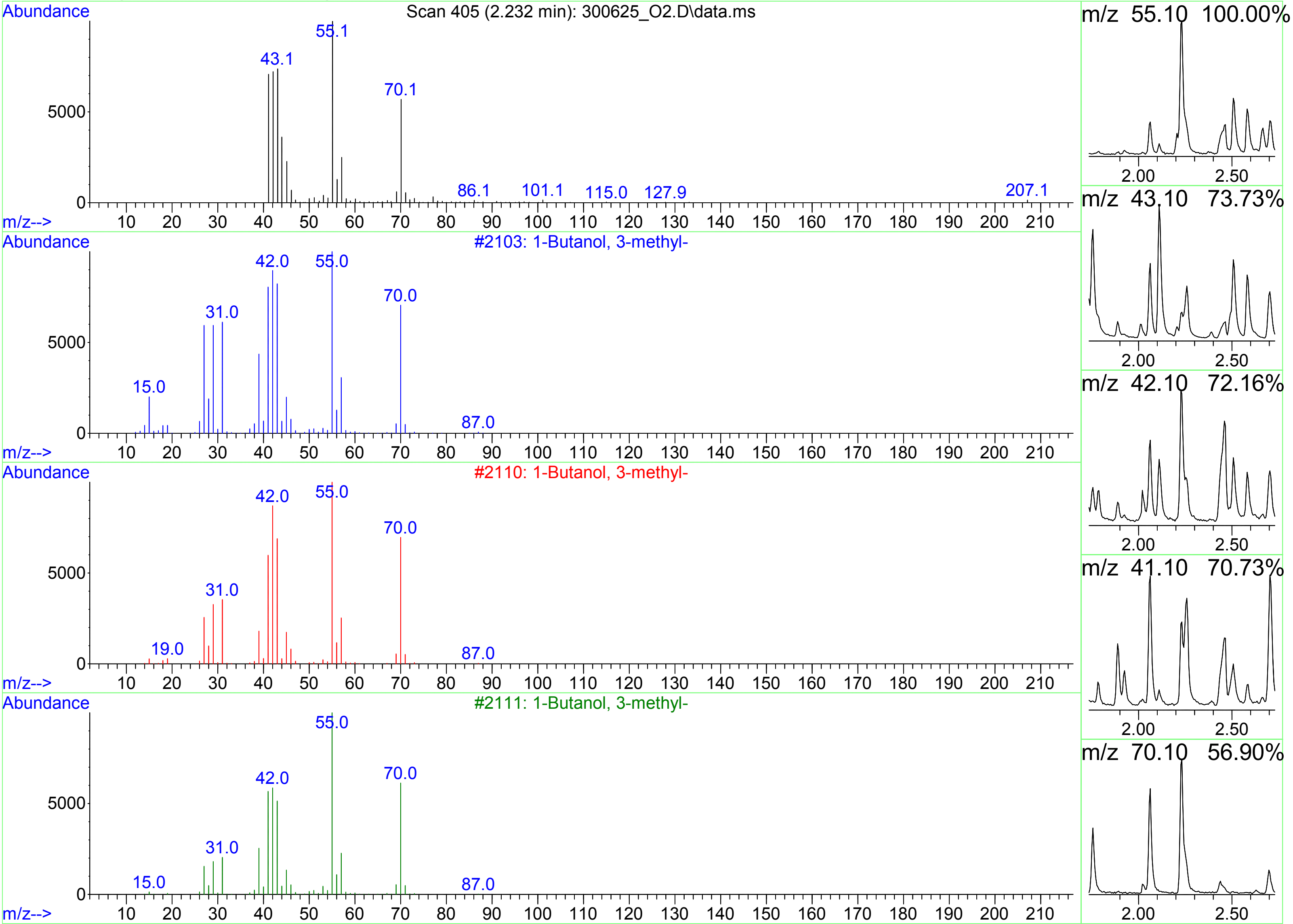

Data File: C:\msdchem\1\data\2025\Docentes\Sussulini\Romani\300625_O2.D
Sample :

Peak Number: 9 at 2.232 min Area: 3157476 Area % 0.55

The 3 best hits from each library.	Ref\#	CAS\#	Qual
C:\Database\NIST08.L			
1 1-Butanol, 3-methyl-	2103	000123-51-3	72
2 1-Butanol, 3-methyl-	2110	000123-51-3	64
3 1-Butanol, 3-methyl-	2111	000123-51-3	64

Unknown Spectrum based on Apex

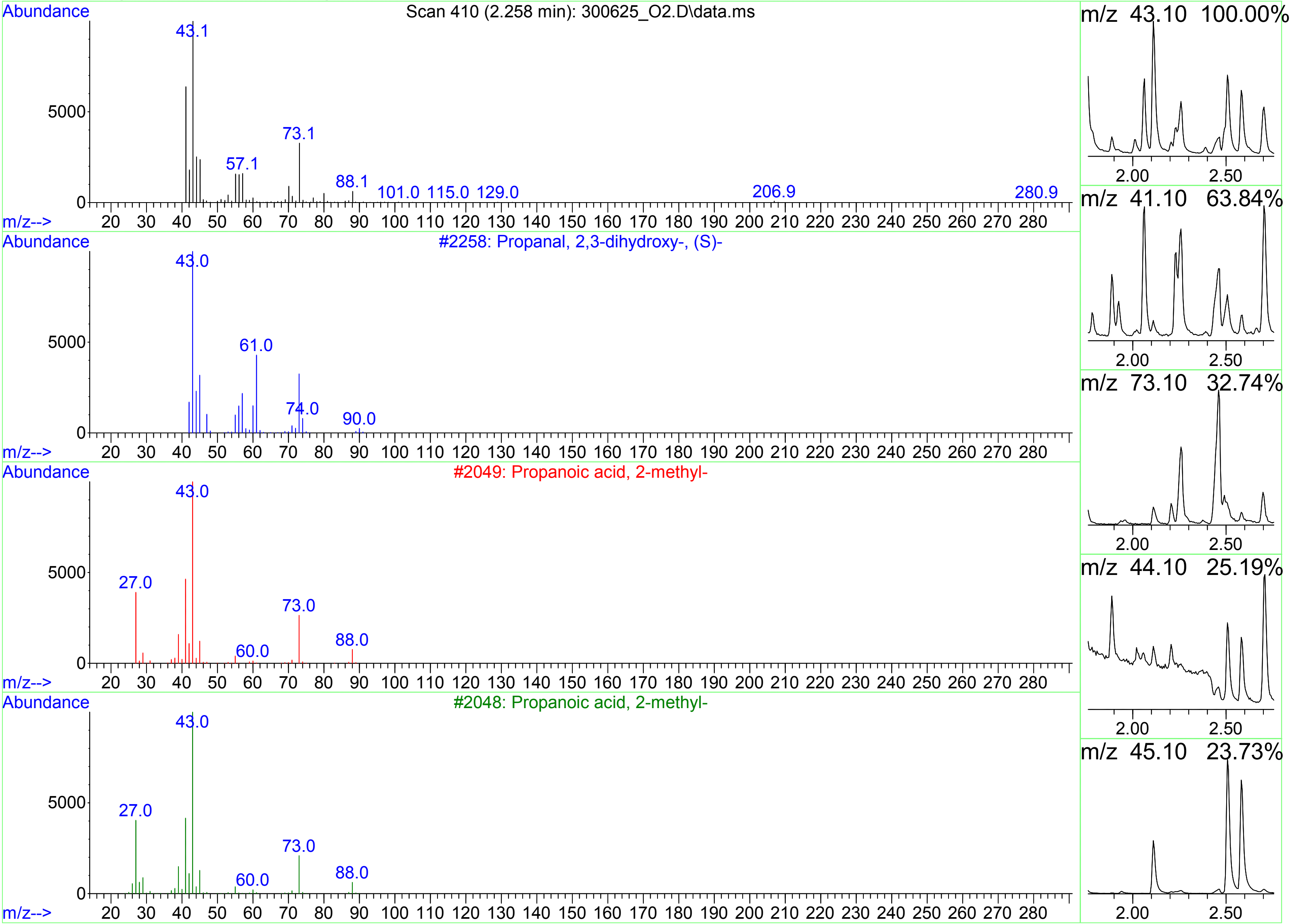

Data File: C:\msdchem\1\data\2025\Docentes\Sussulini\Romani\300625_O2.D
Sample :

Peak Number: 10 at 2.258 min Area: 3227504 Area % 0.56

The 3 best hits from each library.	Ref#	CAS#	Qual
C:\Database\NIST08.L			
1 Propanal, 2,3-dihydroxy-, (S)-	2258	000497-09-6	50
2 Propanoic acid, 2-methyl-	2049	000079-31-2	38
3 Propanoic acid, 2-methyl-	2048	000079-31-2	38

Unknown Spectrum based on Apex

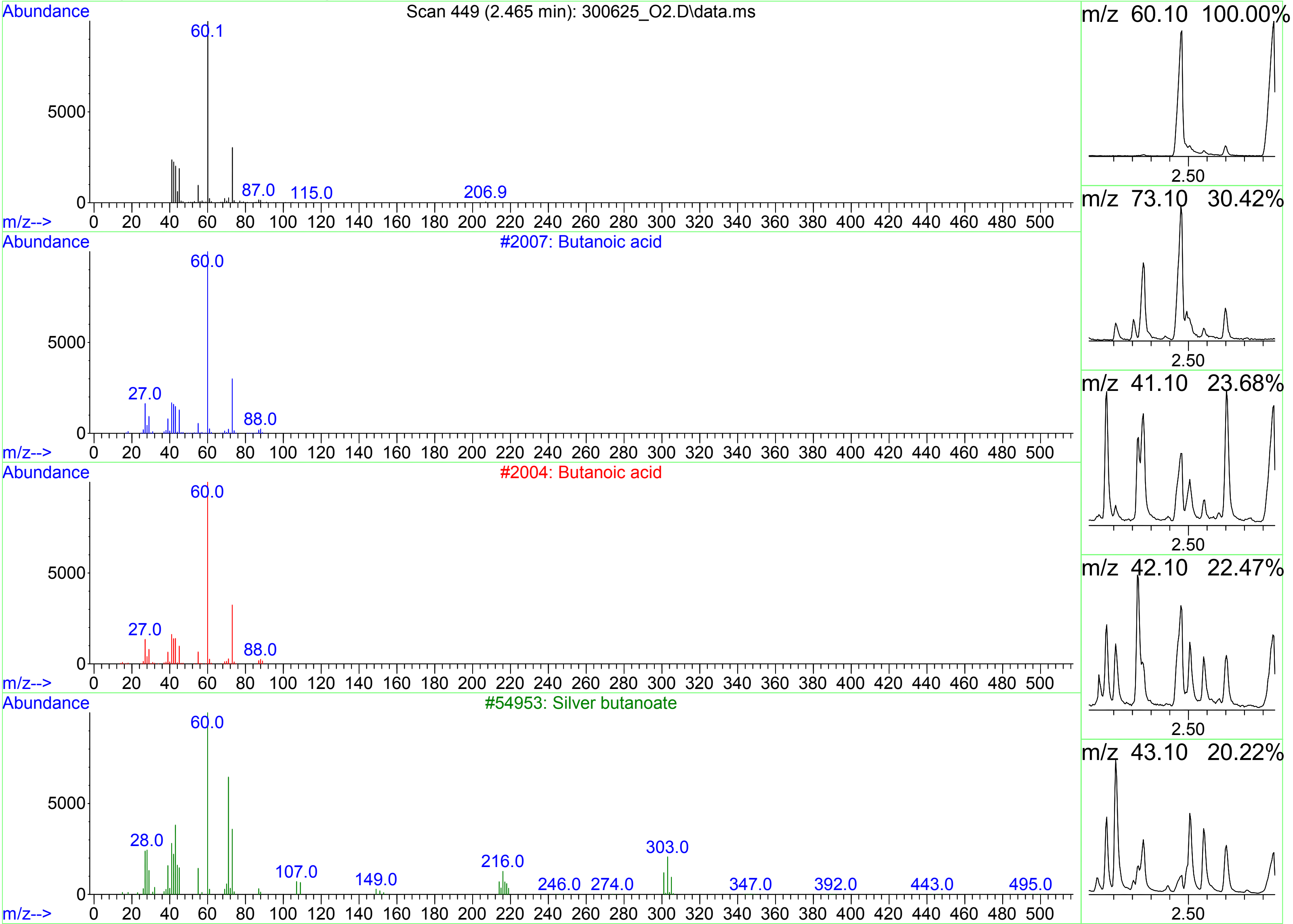

Data File: C:\msdchem\1\data\2025\Docentes\Sussulini\Romani\300625_O2.D
Sample :

Peak Number: 11 at 2.465 min Area: 4482516 Area % 0.78

The 3 best hits from each library.	Ref#	CAS#	Qual
C:\Database\NIST08.L			
1 Butanoic acid	2007	000107-92-6	80
2 Butanoic acid	2004	000107-92-6	56
3 Silver butanoate	54953	005076-24-4	43

Unknown Spectrum based on Apex

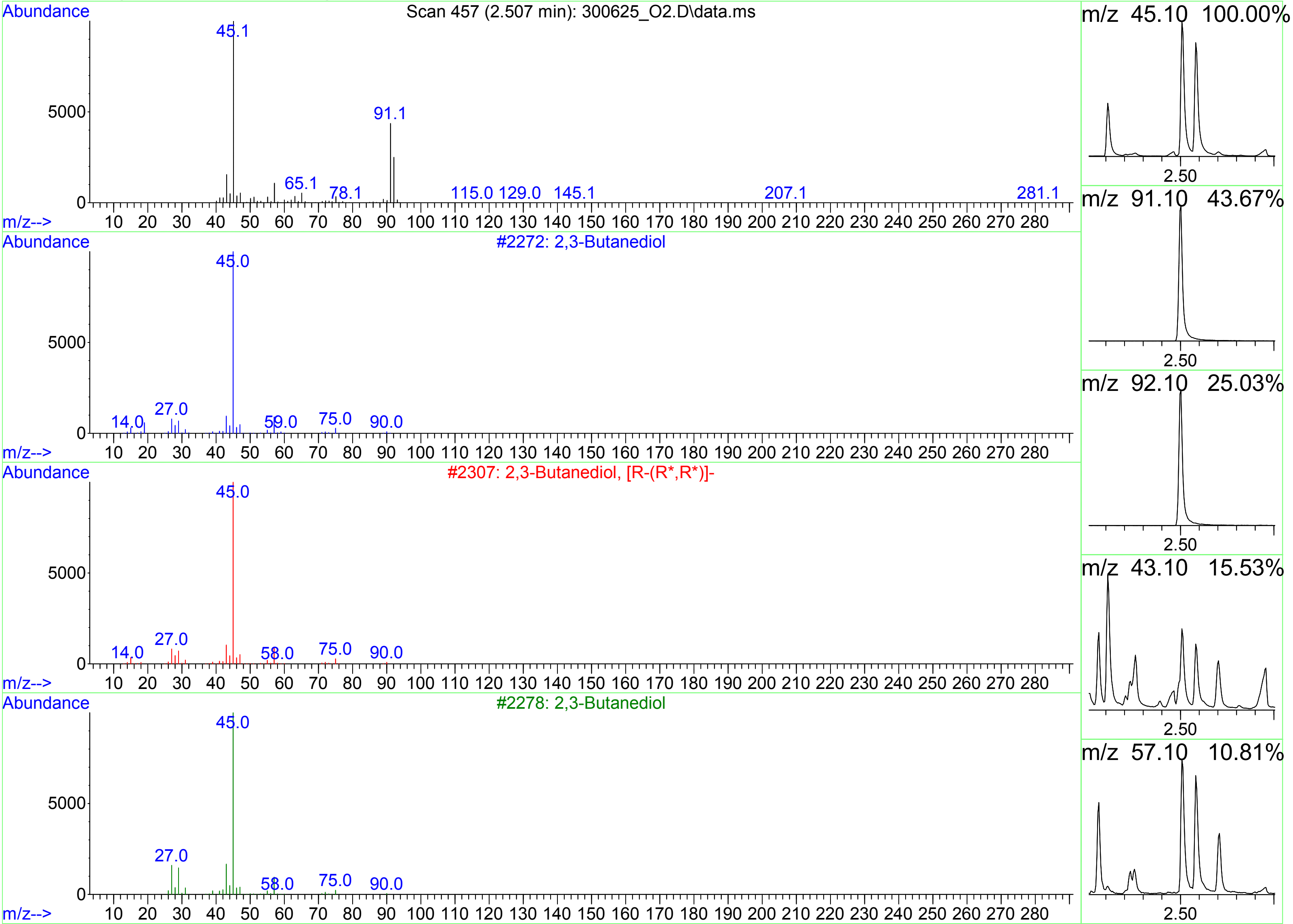

Data File: C:\msdchem\1\data\2025\Docentes\Sussulini\Romani\300625_O2.D
Sample :

Peak Number: 12 at 2.507 min Area: 26317985 Area % 4.60

The 3 best hits from each library.	Ref\#	CAS\#	Qual
C:\Database\NIST08.L			
1 2,3-Butanediol	2272	000513-85-9	64
2 2,3-Butanediol, [R-(R*,R*)]-	2307	024347-58-8	64
3 2,3-Butanediol	2278	000513-85-9	59

Unknown Spectrum based on Apex

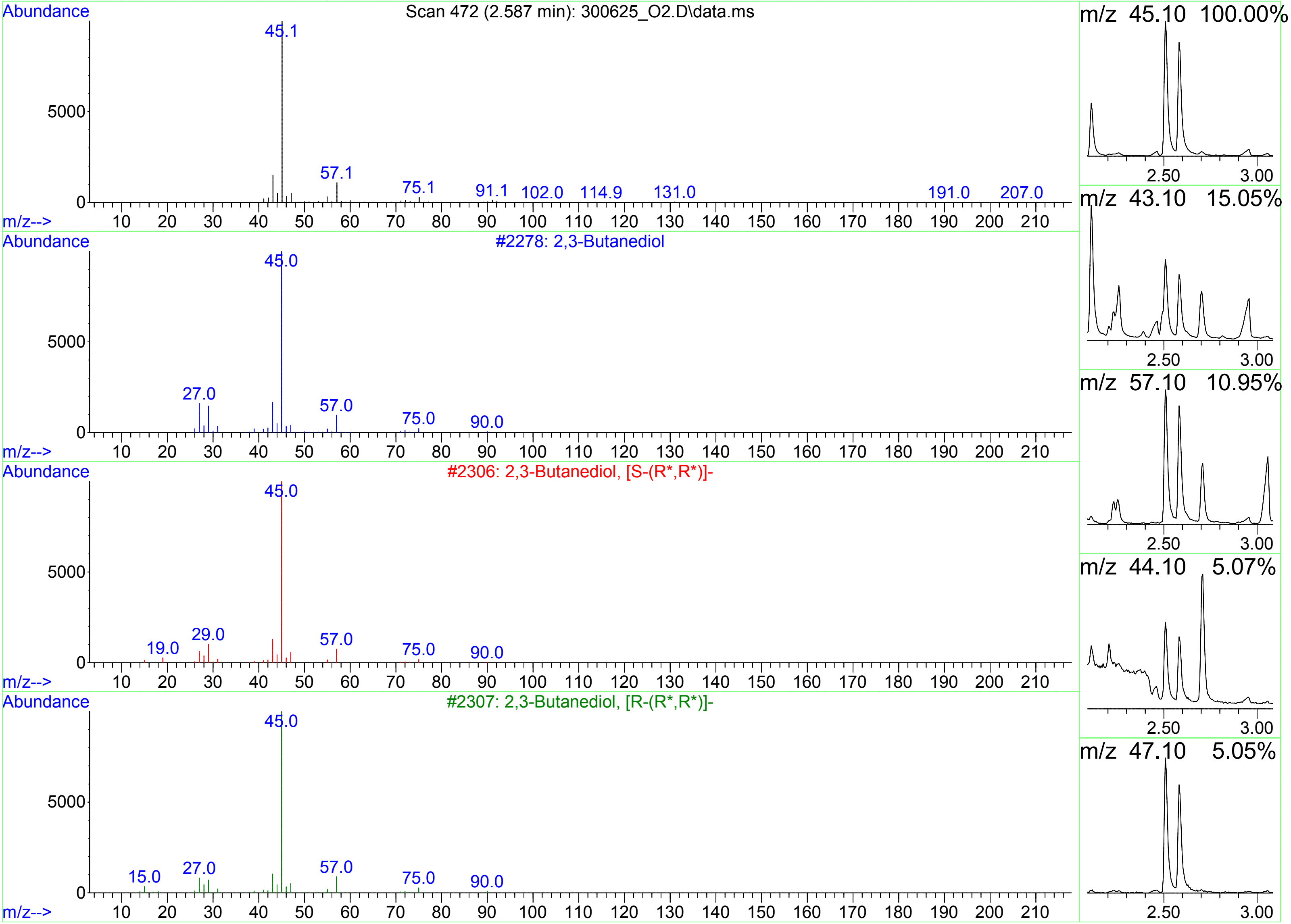

Data File: C:\msdchem\1\data\2025\Docentes\Sussulini\Romani\300625_O2.D
Sample :

Peak Number: 13 at 2.587 min Area: 12812556 Area % 2.24

The 3 best hits from each library.	Ref#	CAS#	Qual
C:\Database\NIST08.L			
1 2,3-Butanediol	2278	000513-85-9	86
2 2,3-Butanediol, [S-(R*,R*)]-	2306	019132-06-0	78
3 2,3-Butanediol, [R-(R*,R*)]-	2307	024347-58-8	78

Library Search Report - ChemStation Integrator

Unknown Spectrum based on Apex

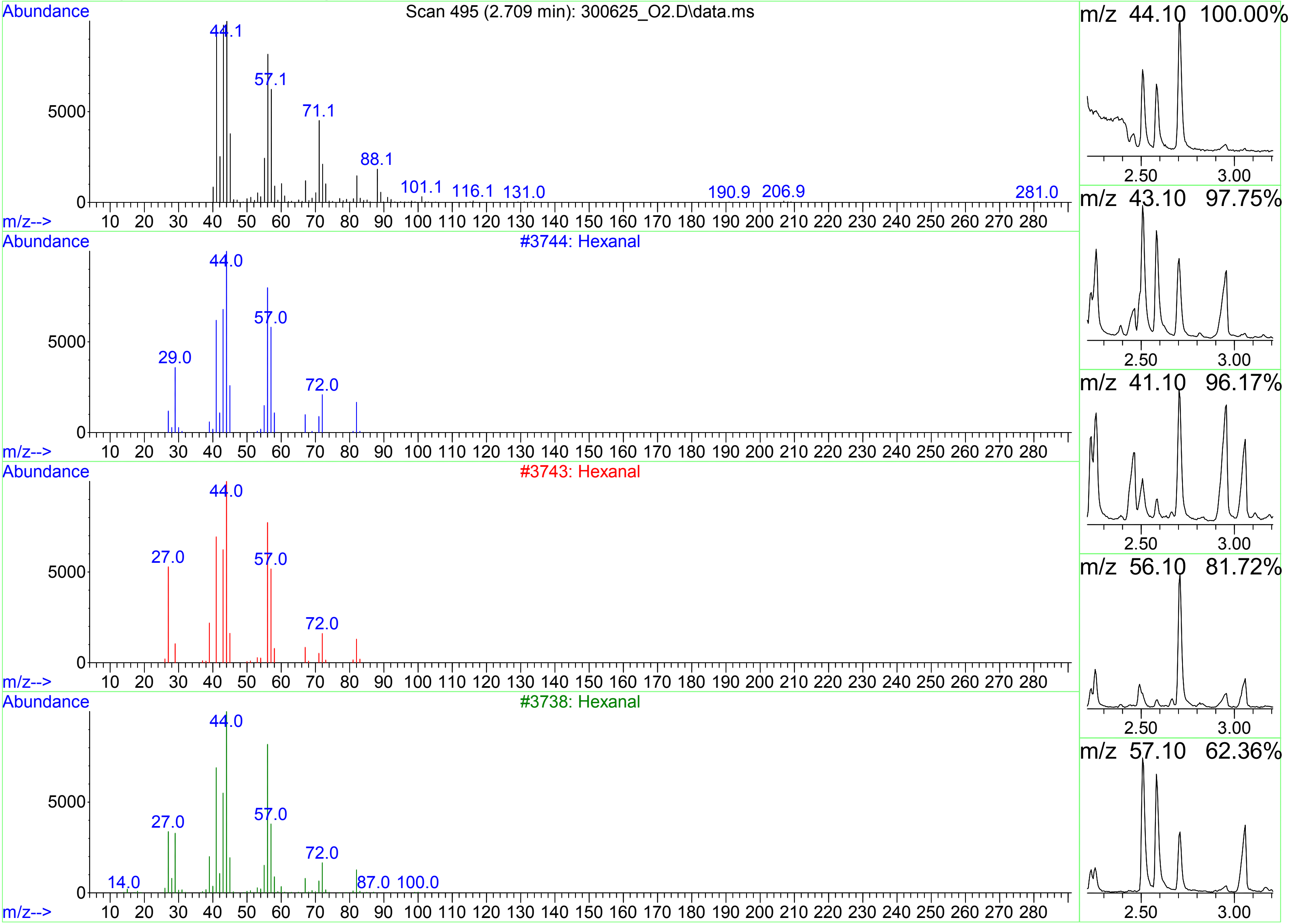

Data File: C:\msdchem\1\data\2025\Docentes\Sussulini\Romani\300625_O2.D
Sample :

Peak Number: 14 at 2.709 min Area: 5864772 Area % 1.03

The 3 best hits from each library.	Ref#	CAS#	Qual
C:\Database\NIST08.L			
1 Hexanal	3744	000066-25-1	53
2 Hexanal	3743	000066-25-1	50
3 Hexanal	3738	000066-25-1	45

Library Search Report - ChemStation Integrator

Unknown Spectrum based on Apex

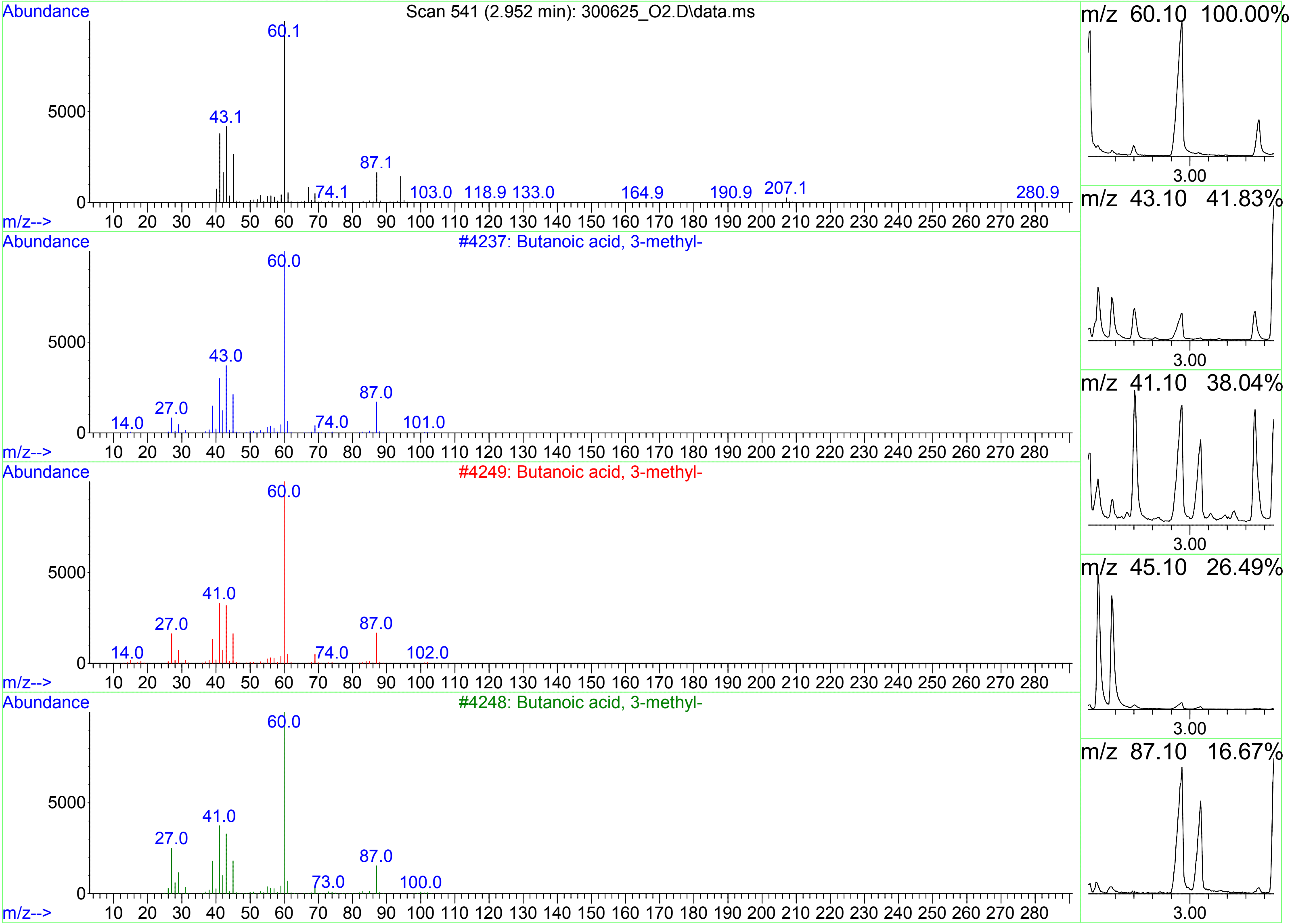

Data File: C:\msdchem\1\data\2025\Docentes\Sussulini\Romani\300625_O2.D
Sample :

Peak Number: 15 at 2.952 min Area: 7400975 Area % 1.29

The 3 best hits from each library.	Ref#	CAS#	Qual
C:\Database\NIST08.L			
1 Butanoic acid, 3-methyl-	4237	000503-74-2	72
2 Butanoic acid, 3-methyl-	4249	000503-74-2	59
3 Butanoic acid, 3-methyl-	4248	000503-74-2	59

Library Search Report - ChemStation Integrator

Unknown Spectrum based on Apex

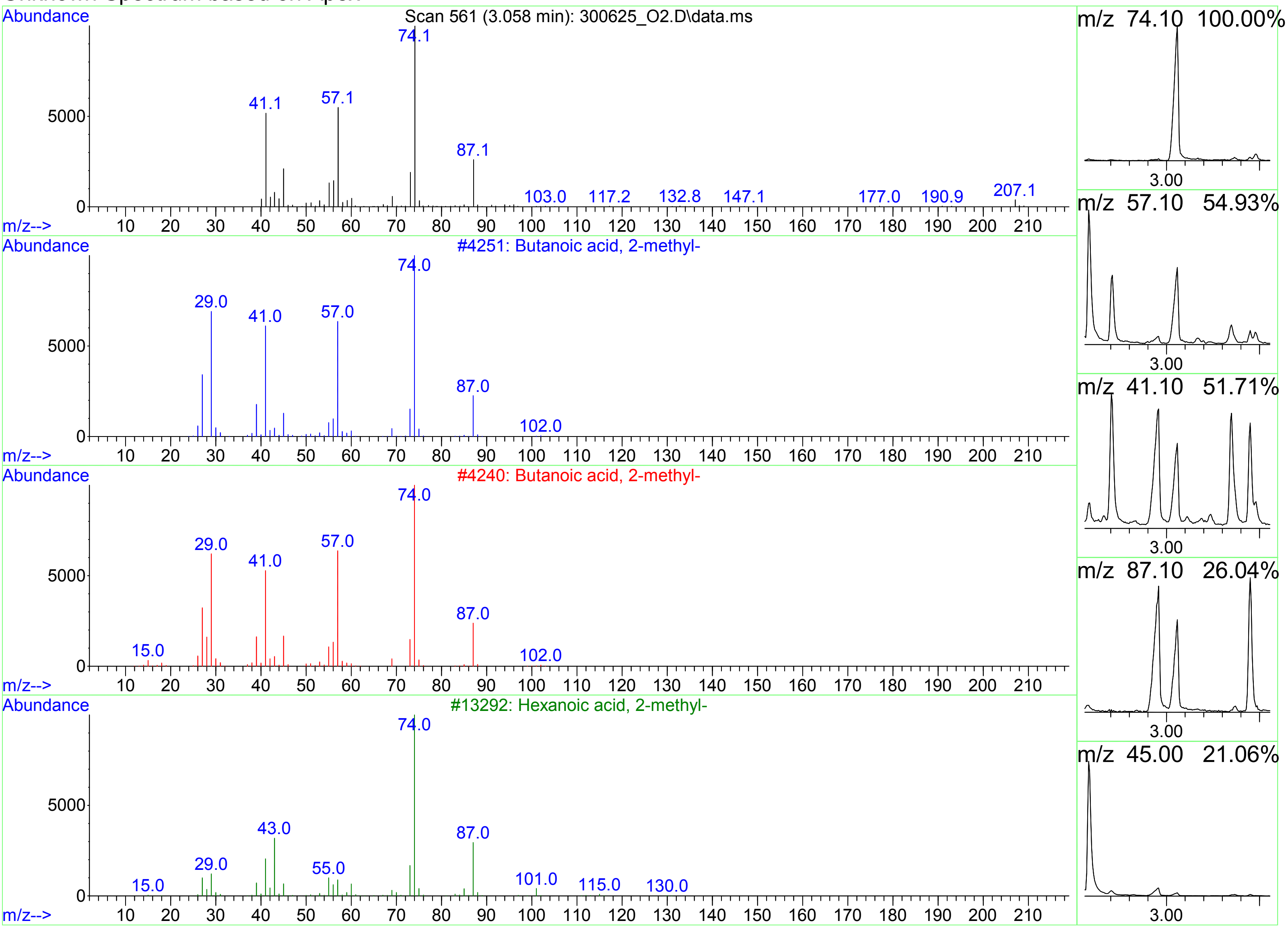

Data File: C:\msdchem\1\data\2025\Docentes\Sussulini\Romani\300625_O2.D
Sample :

Peak Number: 16 at 3.058 min Area: 3255582 Area % 0.57

The 3 best hits from each library. Ref\# CAS\# Qual

C:\Database\NIST08.L

1 Butanoic acid, 2-methyl-	4251	000116-53-0	59
2 Butanoic acid, 2-methyl-	4240	000116-53-0	59
3 Hexanoic acid, 2-methyl-	13292	004536-23-6	56

Unknown Spectrum based on Apex

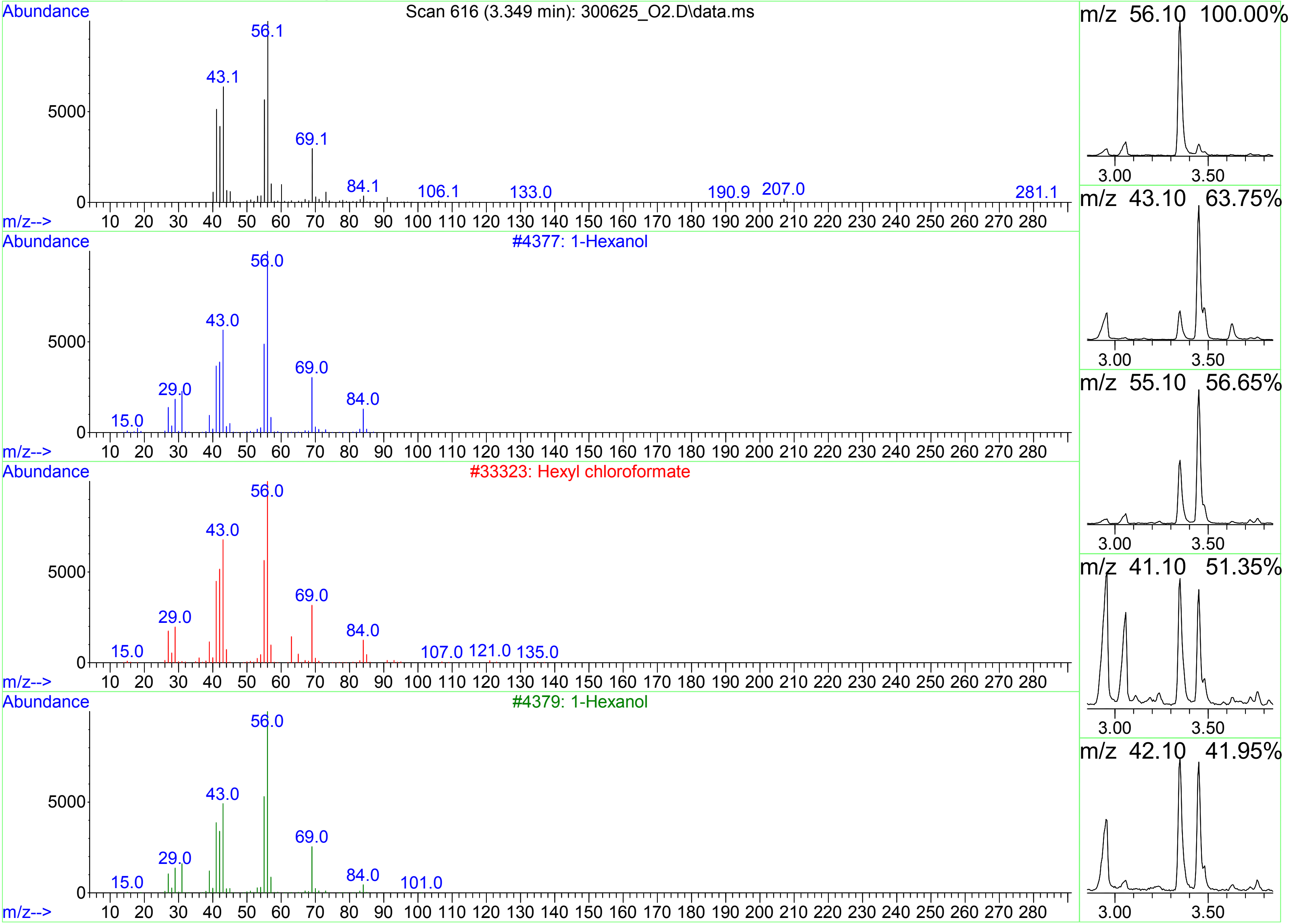

Data File: C:\msdchem\1\data\2025\Docentes\Sussulini\Romani\300625_O2.D
Sample :

Peak Number: 17 at 3.349 min Area: 5837707 Area % 1.02

The 3 best hits from each library.	Ref#	CAS#	Qual
C:\Database\NIST08.L			
1 1-Hexanol	4377	000111-27-3	72
2 Hexyl chloroformate	33323	006092-54-2	72
3 1-Hexanol	4379	000111-27-3	72

Unknown Spectrum based on Apex

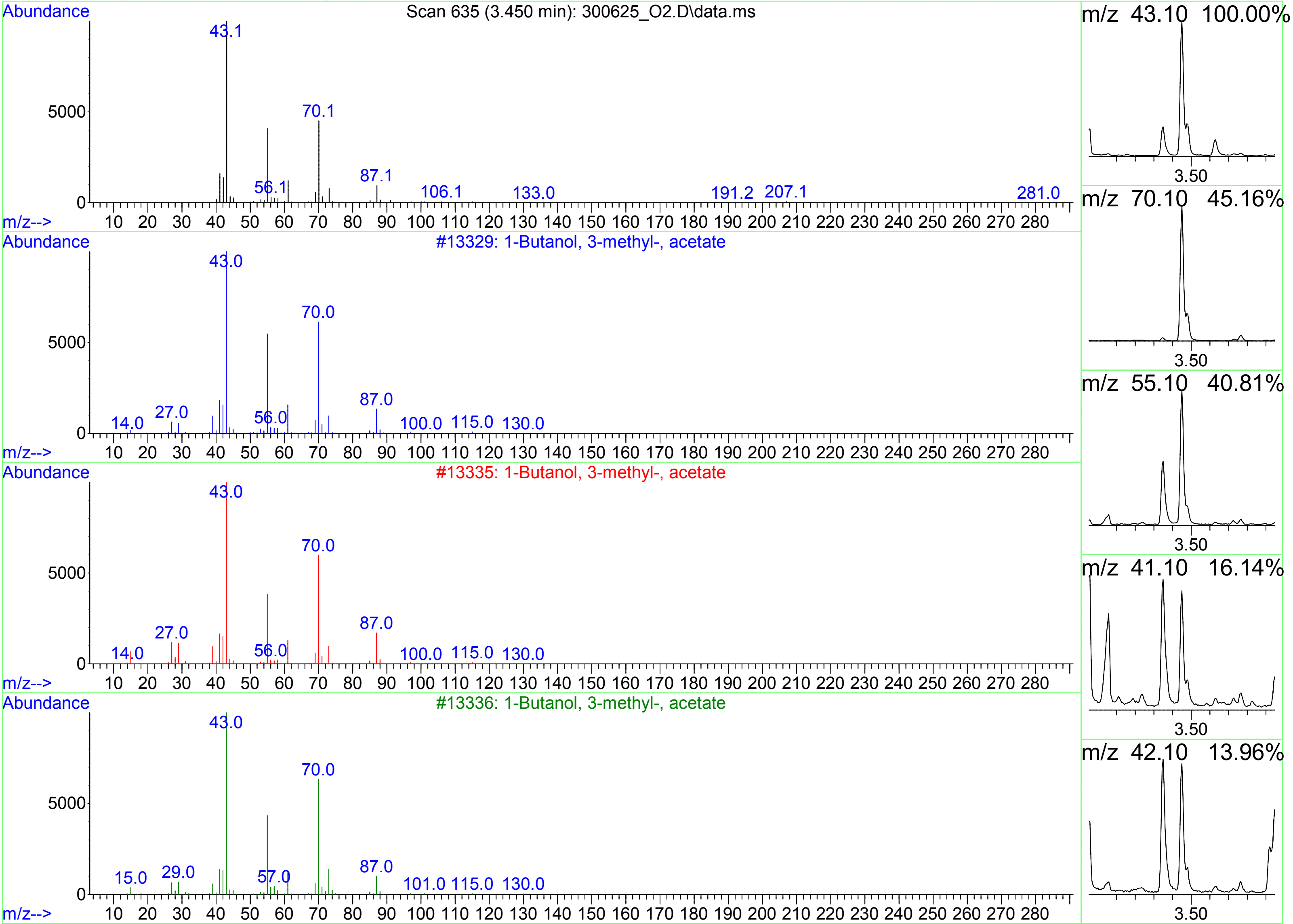

Data File: C:\msdchem\1\data\2025\Docentes\Sussulini\Romani\300625_O2.D
Sample :

Peak Number: 18 at 3.450 min Area: 10946467 Area % 1.92

The 3 best hits from each library.	Ref#	CAS#	Qual
C:\Database\NIST08.L			
1 1-Butanol, 3-methyl-, acetate	13329	000123-92-2	90
2 1-Butanol, 3-methyl-, acetate	13335	000123-92-2	72
3 1-Butanol, 3-methyl-, acetate	13336	000123-92-2	72

Unknown Spectrum based on Apex

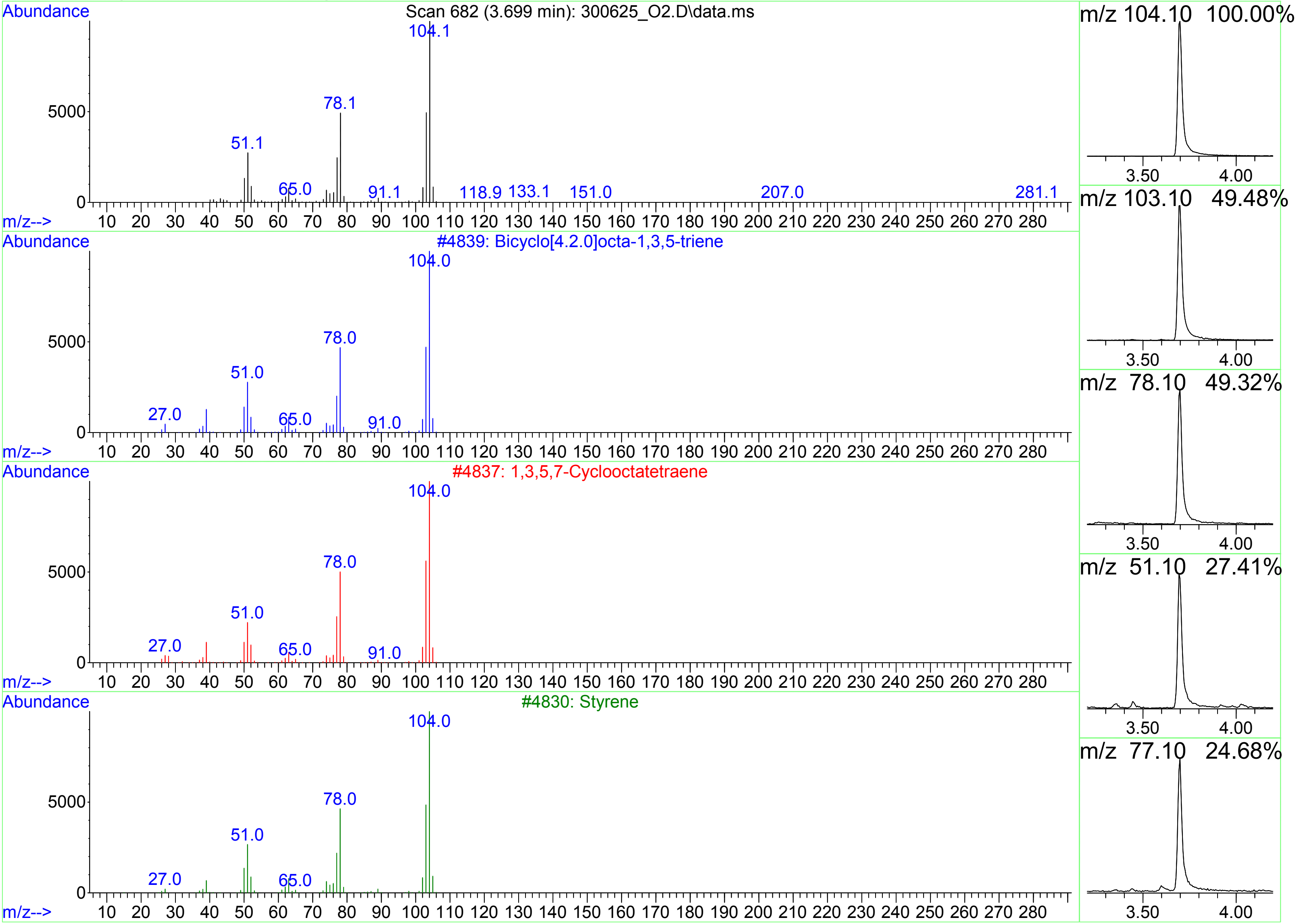

Data File: C:\msdchem\1\data\2025\Docentes\Sussulini\Romani\300625_O2.D
Sample :

Peak Number: 19 at 3.699 min Area: 8794285 Area % 1.54

The 3 best hits from each library.	Ref\#	CAS\#	Qual
C:\Database\NIST08.L			
1 Bicyclo[4.2.0]octa-1,3,5-triene	4839	000694-87-1	96
2 1,3,5,7-Cyclooctatetraene	4837	000629-20-9	96
3 Styrene	4830	000100-42-5	96

Unknown Spectrum based on Apex

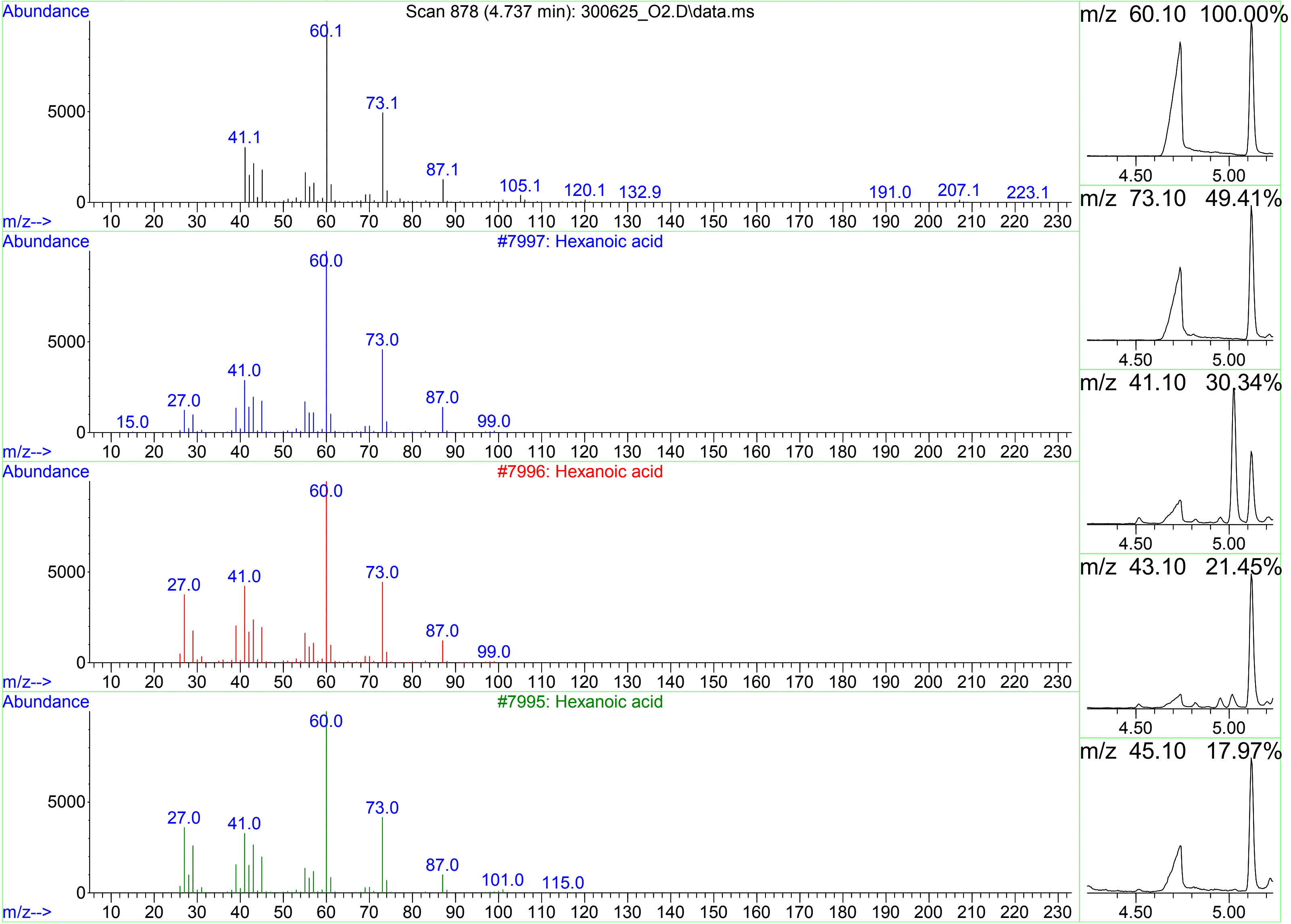

Data File: C:\msdchem\1\data\2025\Docentes\Sussulini\Romani\300625_O2.D
Sample :

Peak Number: 20 at 4.737 min Area: 15672112 Area % 2.74

The 3 best hits from each library.	Ref#	CAS#	Qual
C:\Database\NIST08.L			
1 Hexanoic acid	7997	000142-62-1	90
2 Hexanoic acid	7996	000142-62-1	90
3 Hexanoic acid	7995	000142-62-1	83

Unknown Spectrum based on Apex

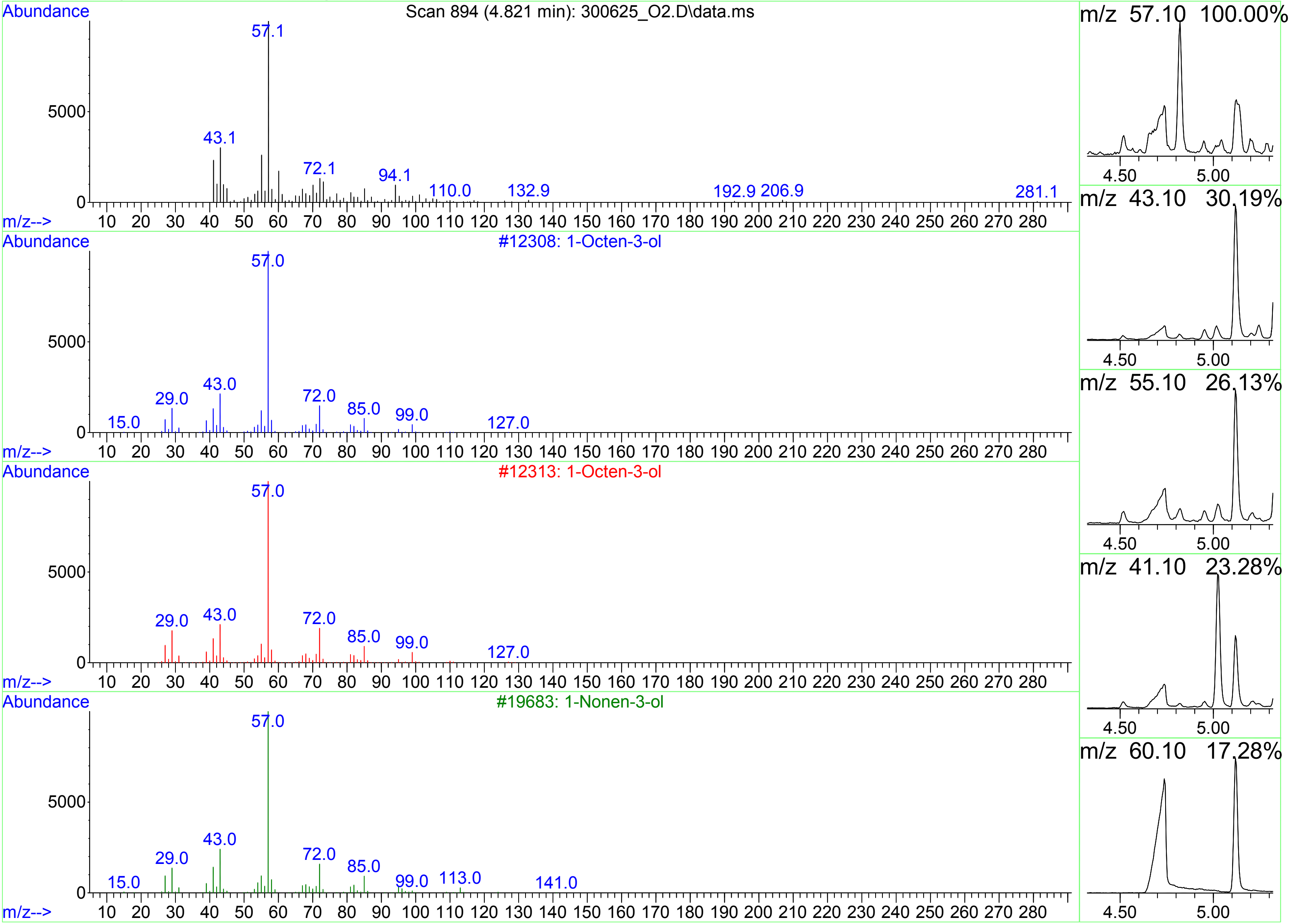

Data File: C:\msdchem\1\data\2025\Docentes\Sussulini\Romani\300625_O2.D
Sample :

Peak Number: 21 at 4.821 min Area: 2869028 Area % 0.50

The 3 best hits from each library.	Ref#	CAS#	Qual
C:\Database\NIST08.L			
1 1-Octen-3-ol	12308	003391-86-4	38
2 1-Octen-3-ol	12313	003391-86-4	35
3 1-Nonen-3-ol	19683	021964-44-3	35

Unknown Spectrum based on Apex

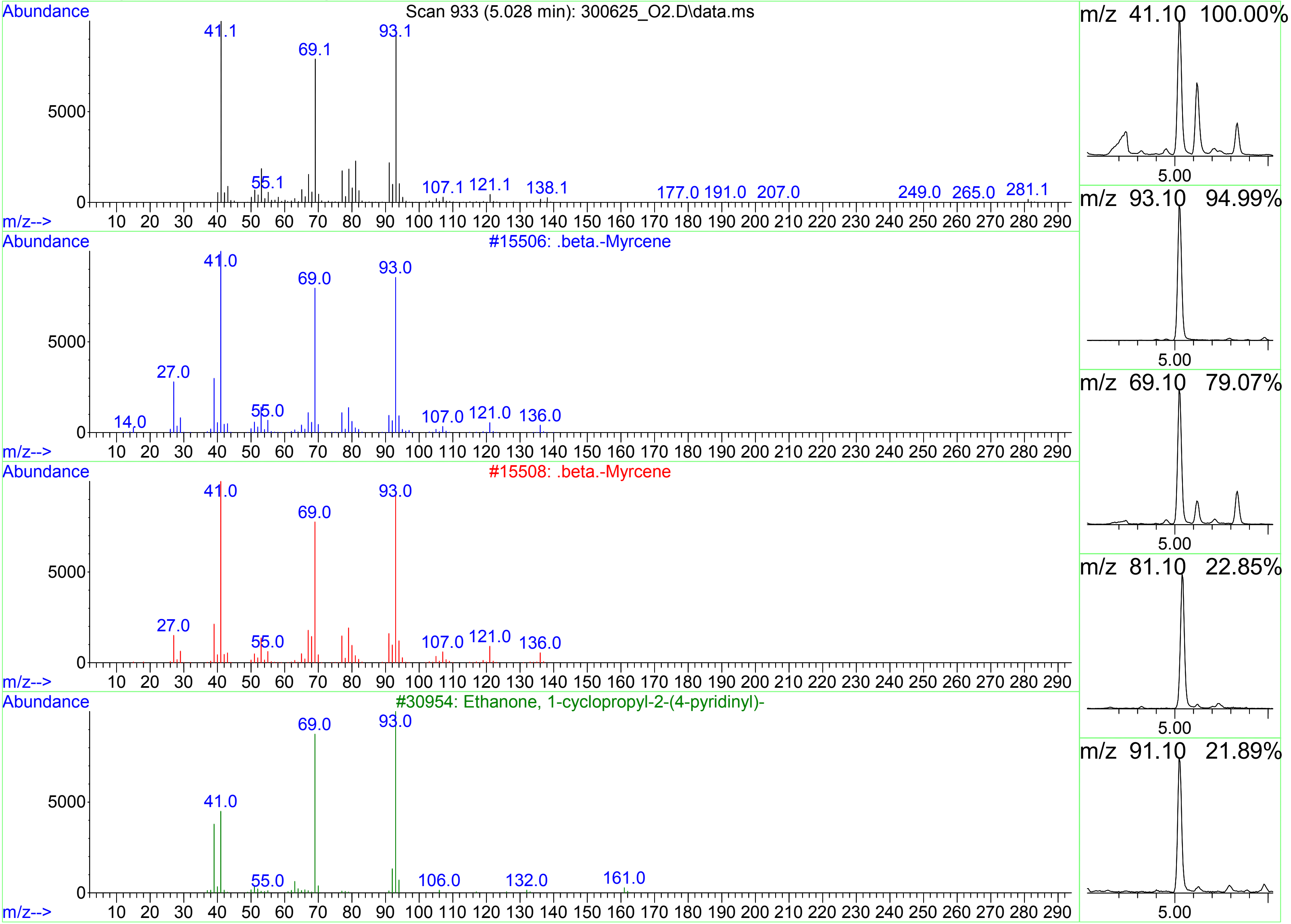

Data File: C:\msdchem\1\data\2025\Docentes\Sussulini\Romani\300625_O2.D
Sample :

Peak Number: 22 at 5.028 min Area: 18376506 Area % 3.22

The 3 best hits from each library.	Ref\#	CAS\#	Qual
C:\Database\NIST08.L			
1 .beta.-Myrcene	15506	000123-35-3	91
2 .beta.-Myrcene	15508	000123-35-3	83
3 Ethanone, 1-cyclopropyl-2-(4-pyr...	30954	006580-95-6	64

Library Search Report - ChemStation Integrator

Unknown Spectrum based on Apex

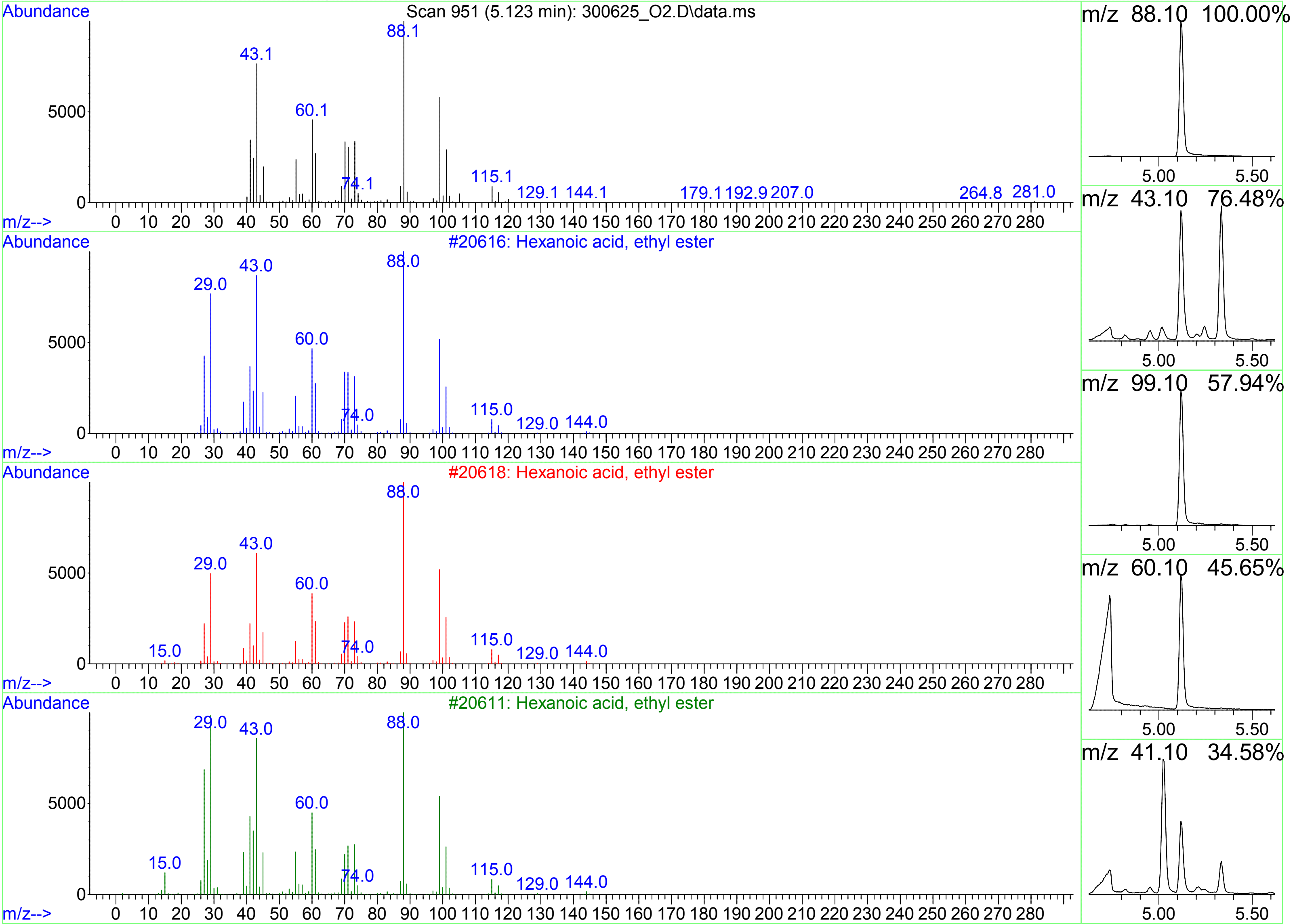

Data File: C:\msdchem\1\data\2025\Docentes\Sussulini\Romani\300625_O2.D
Sample :

Peak Number: 23 at 5.123 min Area: 30304020 Area % 5.30

The 3 best hits from each library.	Ref#	CAS#	Qual
C:\Database\NIST08.L			
1 Hexanoic acid, ethyl ester	20616	000123-66-0	91
2 Hexanoic acid, ethyl ester	20618	000123-66-0	91
3 Hexanoic acid, ethyl ester	20611	000123-66-0	90

Library Search Report - ChemStation Integrator

Unknown Spectrum based on Apex

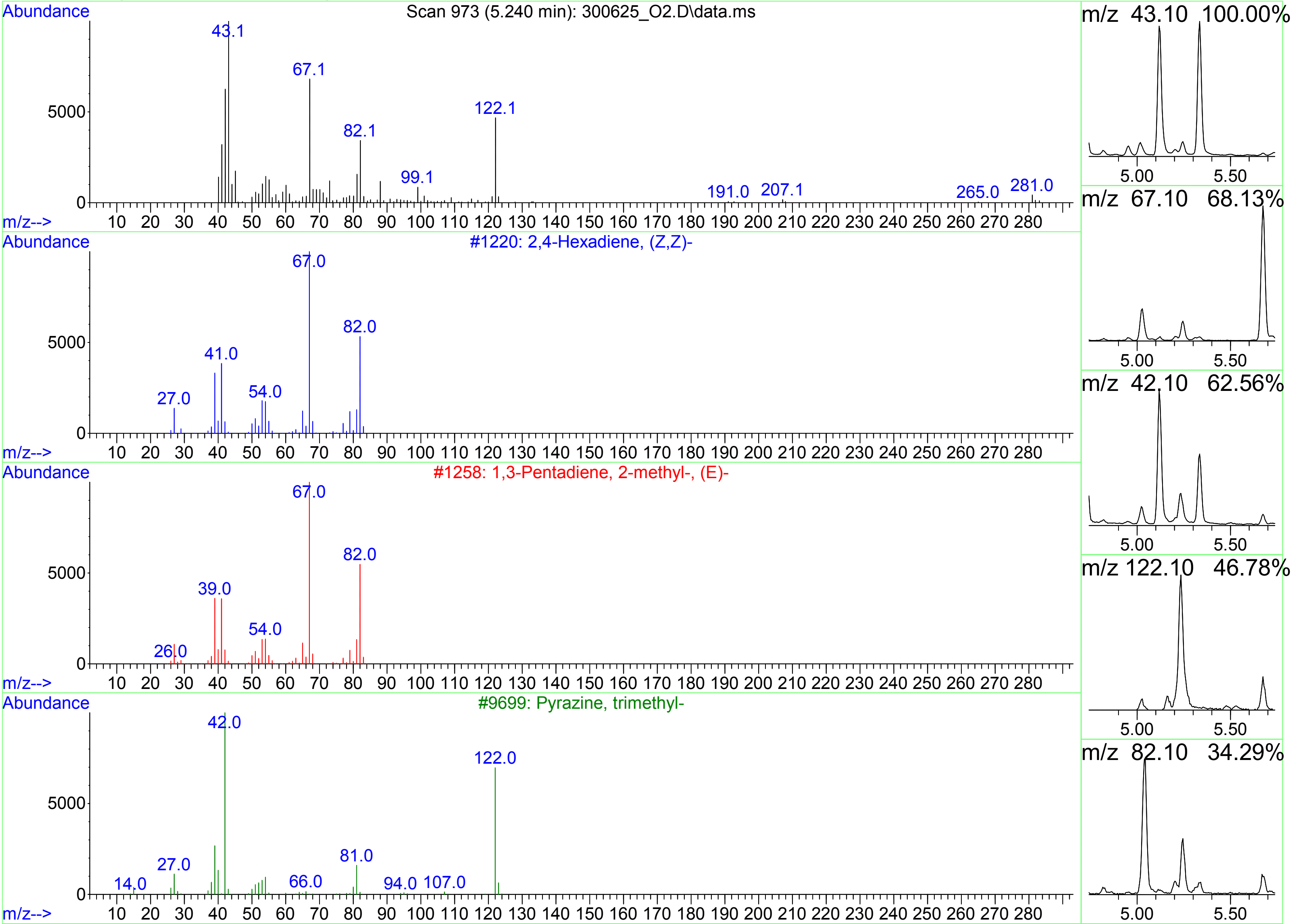

Data File: C:\msdchem\1\data\2025\Docentes\Sussulini\Romani\300625_O2.D
Sample :

Peak Number: 24 at 5.240 min Area: 4544482 Area % 0.80

The 3 best hits from each library.	Ref\#	CAS\#	Qual
C:\Database\NIST08.L			
1 2,4-Hexadiene, (Z,Z)-	1220	006108-61-8	35
2 1,3-Pentadiene, 2-methyl-, (E)-	1258	000926-54-5	30
3 Pyrazine, trimethyl-	9699	014667-55-1	30

Unknown Spectrum based on Apex

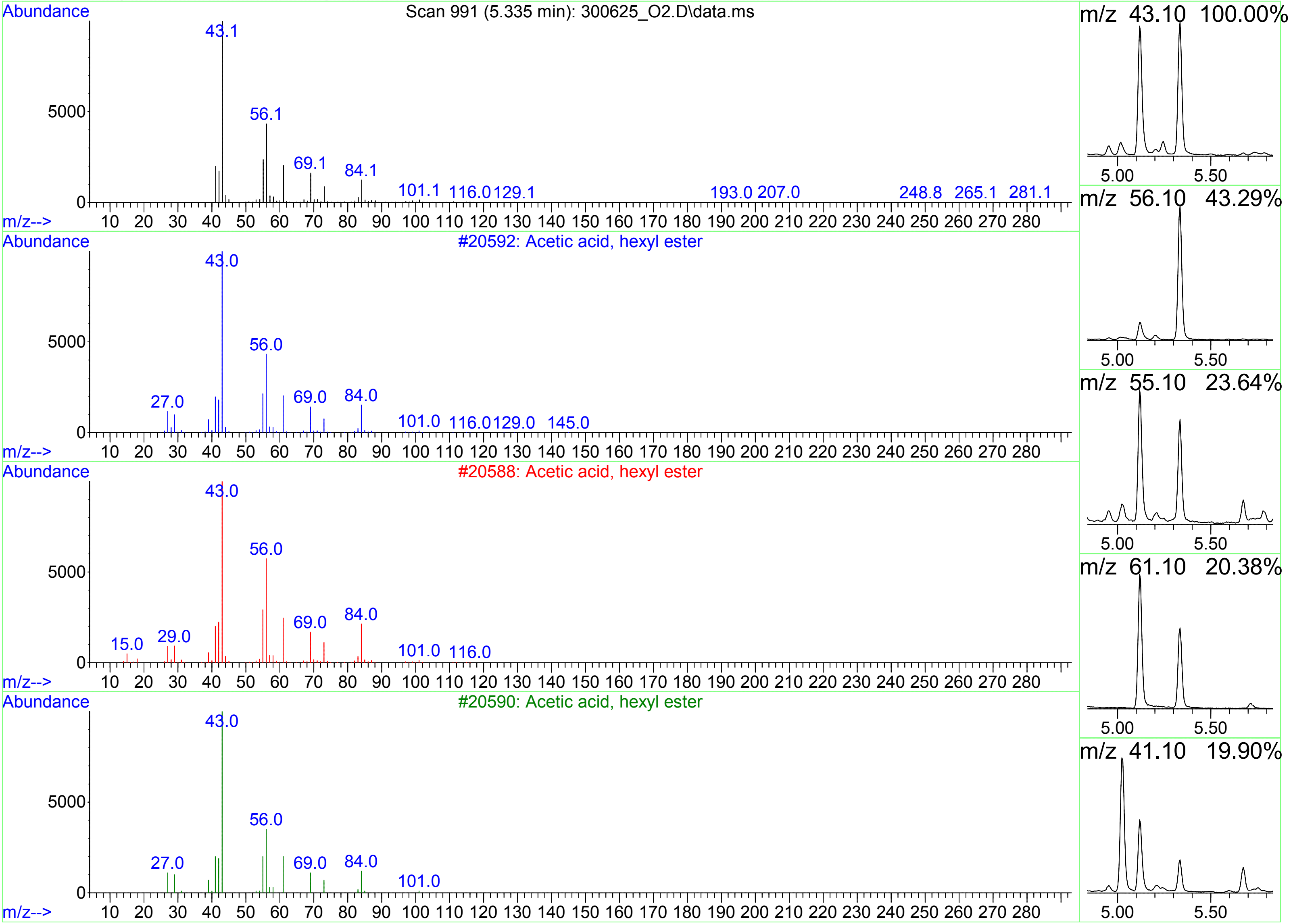

Data File: C:\msdchem\1\data\2025\Docentes\Sussulini\Romani\300625_O2.D
Sample :

Peak Number: 25 at 5.335 min Area: 11907298 Area % 2.08

The 3 best hits from each library.	Ref#	CAS#	Qual
C:\Database\NIST08.L			
1 Acetic acid, hexyl ester	20592	000142-92-7	86
2 Acetic acid, hexyl ester	20588	000142-92-7	86
3 Acetic acid, hexyl ester	20590	000142-92-7	78

Unknown Spectrum based on Apex

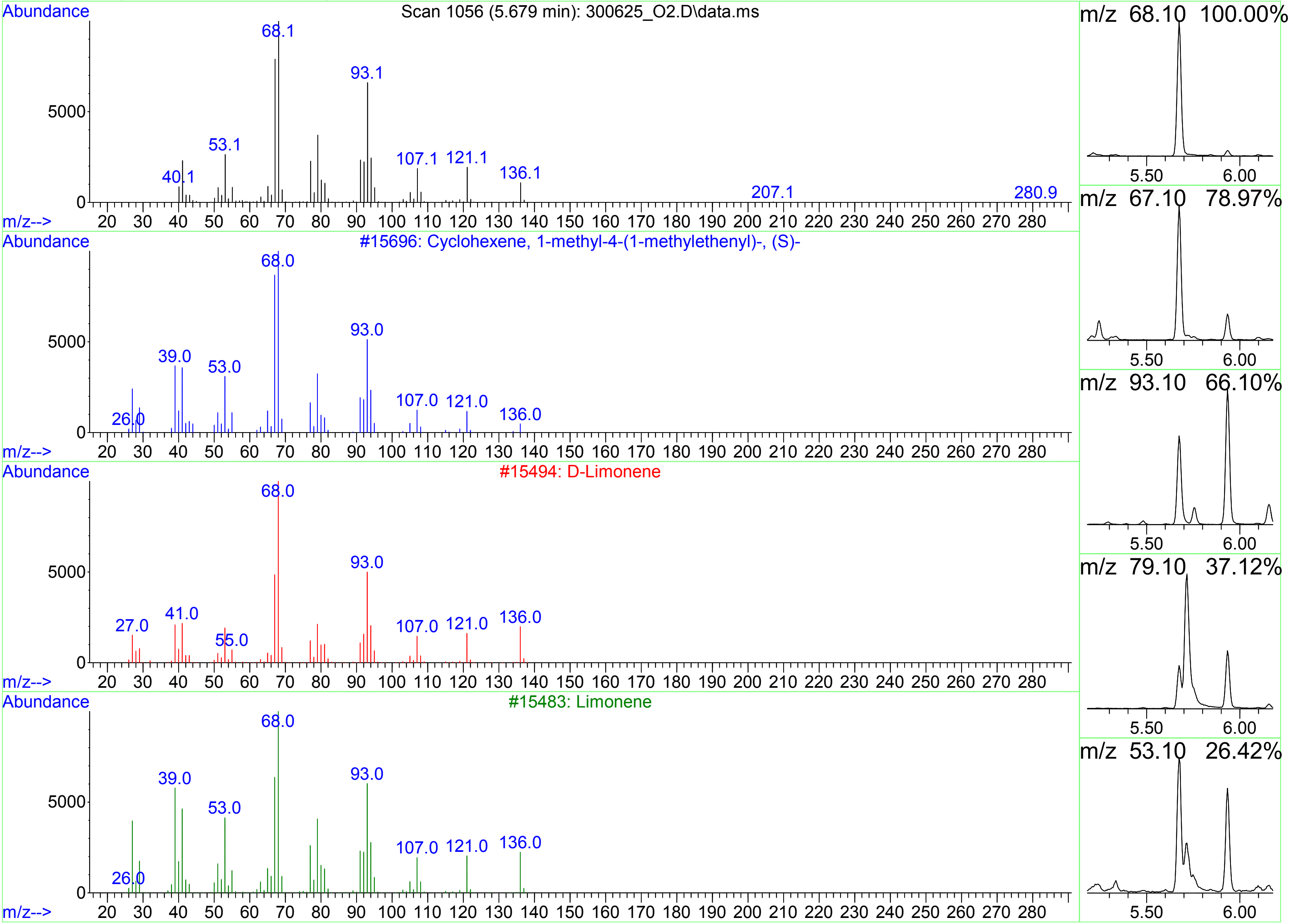

Data File: C:\msdchem\1\data\2025\Docentes\Sussulini\Romani\300625_O2.D
Sample :

Peak Number: 26 at 5.679 min Area: 13932593 Area % 2.44

The 3 best hits from each library. Ref\# CAS\# Qual

C:\Database\NIST08.L

1	Cyclohexene, 1-methyl-4-(1-methy...	15696	005989-54-8	94
2	D-Limonene	15494	005989-27-5	93
3	Limonene	15483	000138-86-3	91

Unknown Spectrum based on Apex

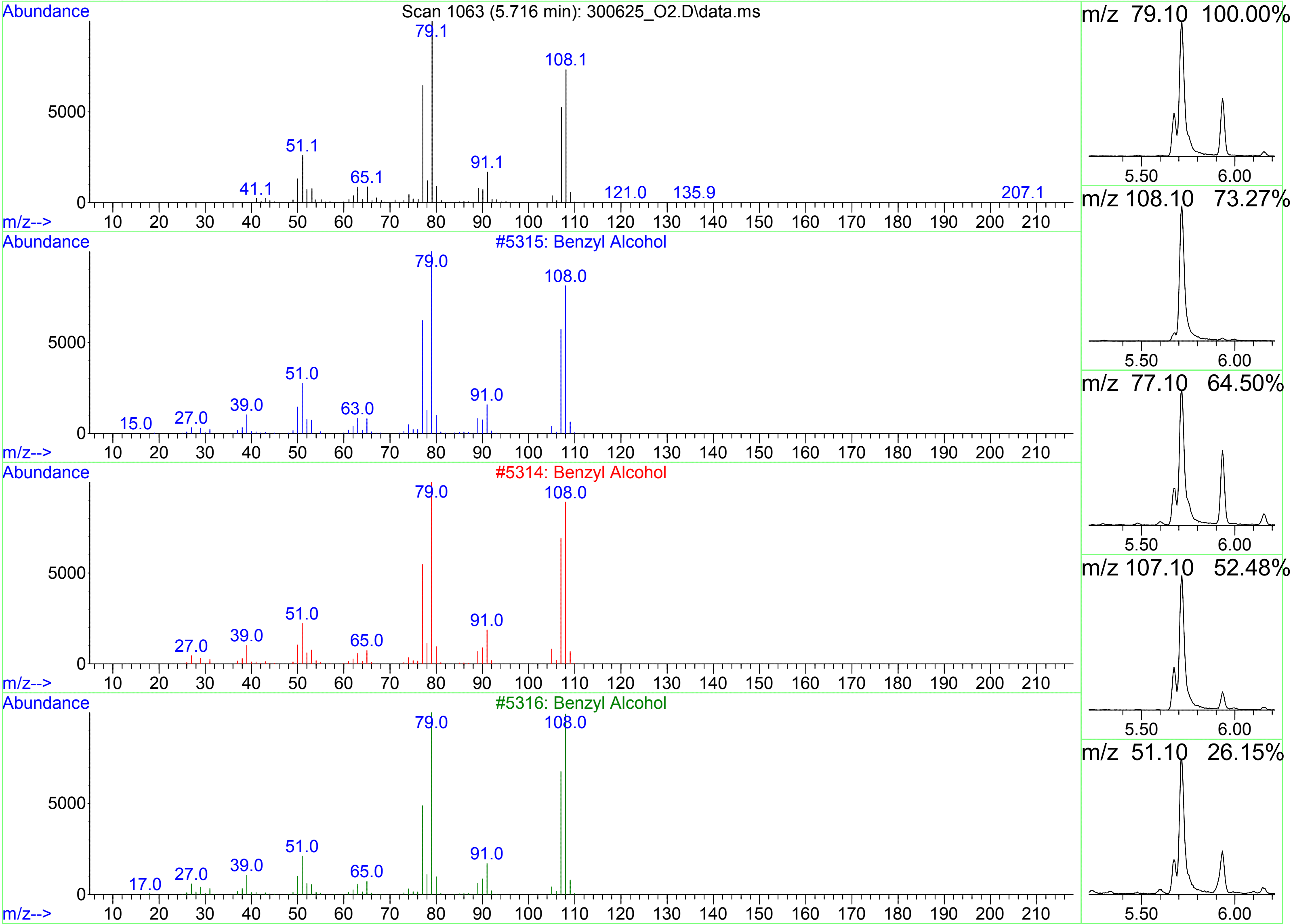

Data File: C:\msdchem\1\data\2025\Docentes\Sussulini\Romani\300625_O2.D
Sample :

Peak Number: 27 at 5.716 min Area: 19328288 Area % 3.38

The 3 best hits from each library.	Ref\#	CAS\#	Qual
C:\Database\NIST08.L			
1 Benzyl Alcohol	5315	000100-51-6	98
2 Benzyl Alcohol	5314	000100-51-6	97
3 Benzyl Alcohol	5316	000100-51-6	96

Library Search Report - ChemStation Integrator

Unknown Spectrum based on Apex

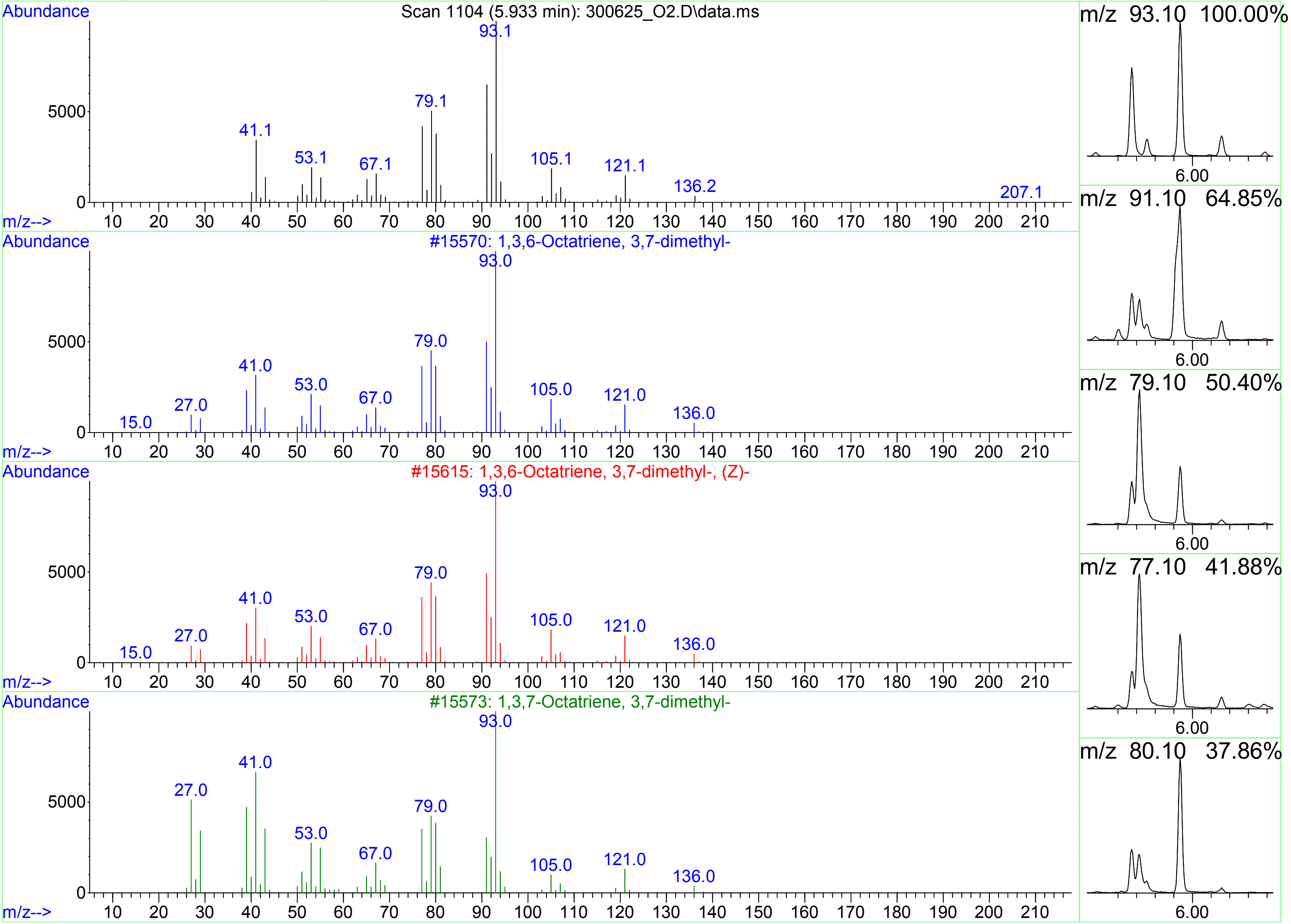

Data File: C:\msdchem\1\data\2025\Docentes\Sussulini\Romani\300625_O2.D
Sample :

Peak Number: 28 at 5.933 min Area: 14927477 Area % 2.61

The 3 best hits from each library.	Ref\#	CAS\#	Qual
C:\Database\NIST08.L			
1 1,3,6-Octatriene, 3,7-dimethyl-	15570	013877-91-3	97
2 1,3,6-Octatriene, 3,7-dimethyl-,...	15615	003338-55-4	96
3 1,3,7-Octatriene, 3,7-dimethyl-	15573	000502-99-8	93

Unknown Spectrum based on Apex

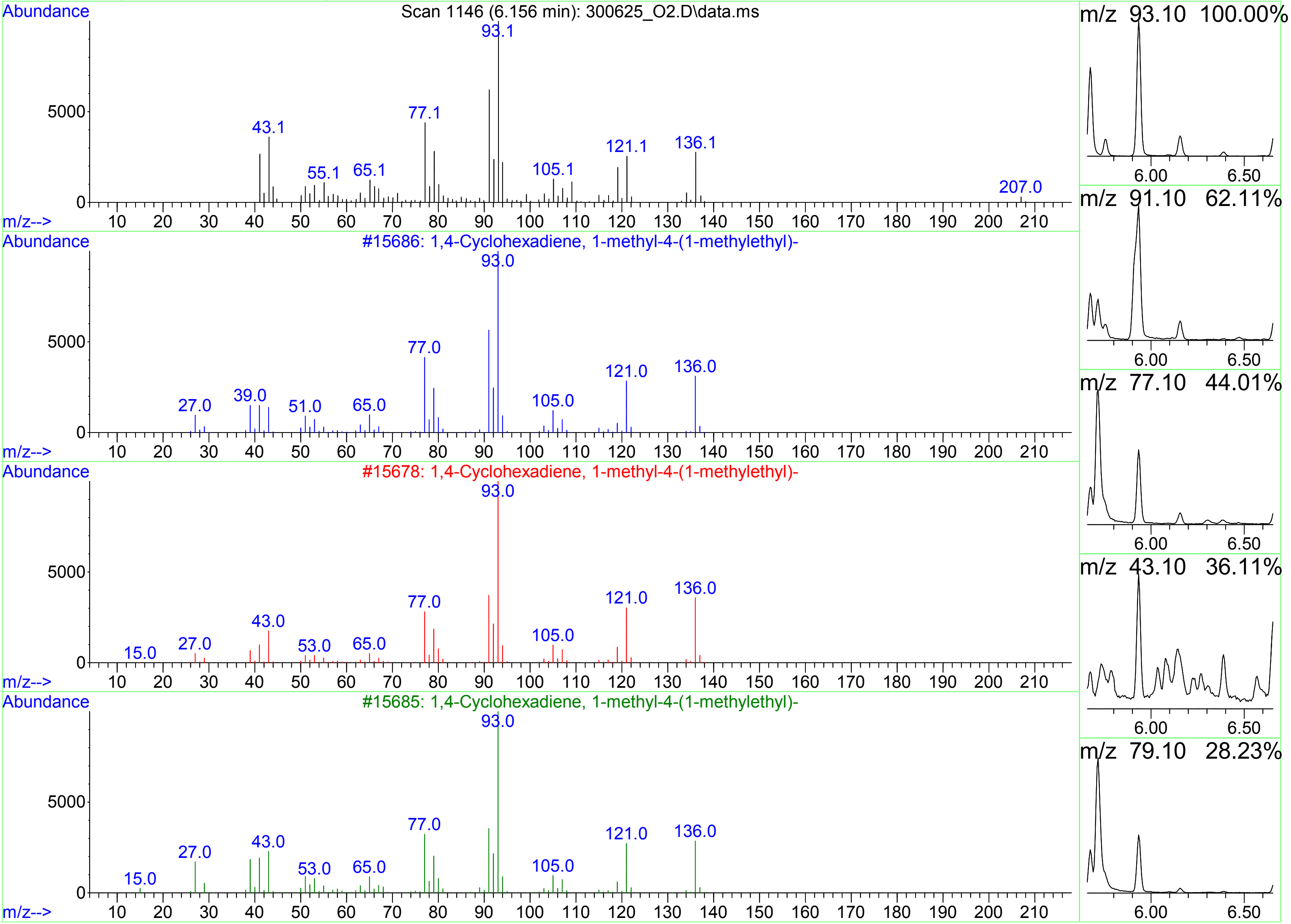

Data File: C:\msdchem\1\data\2025\Docentes\Sussulini\Romani\300625_O2.D
Sample :

Peak Number: 29 at 6.156 min Area: 2466816 Area % 0.43

The 3 best hits from each library. Ref\# CAS\# Qual

C:\Database\NIST08.L

1	1,4-Cyclohexadiene, 1-methyl-4-(...	15686	000099-85-4	96
2	1,4-Cyclohexadiene, 1-methyl-4-(...	15678	000099-85-4	81
3	1,4-Cyclohexadiene, 1-methyl-4-(...	15685	000099-85-4	81

Unknown Spectrum based on Apex

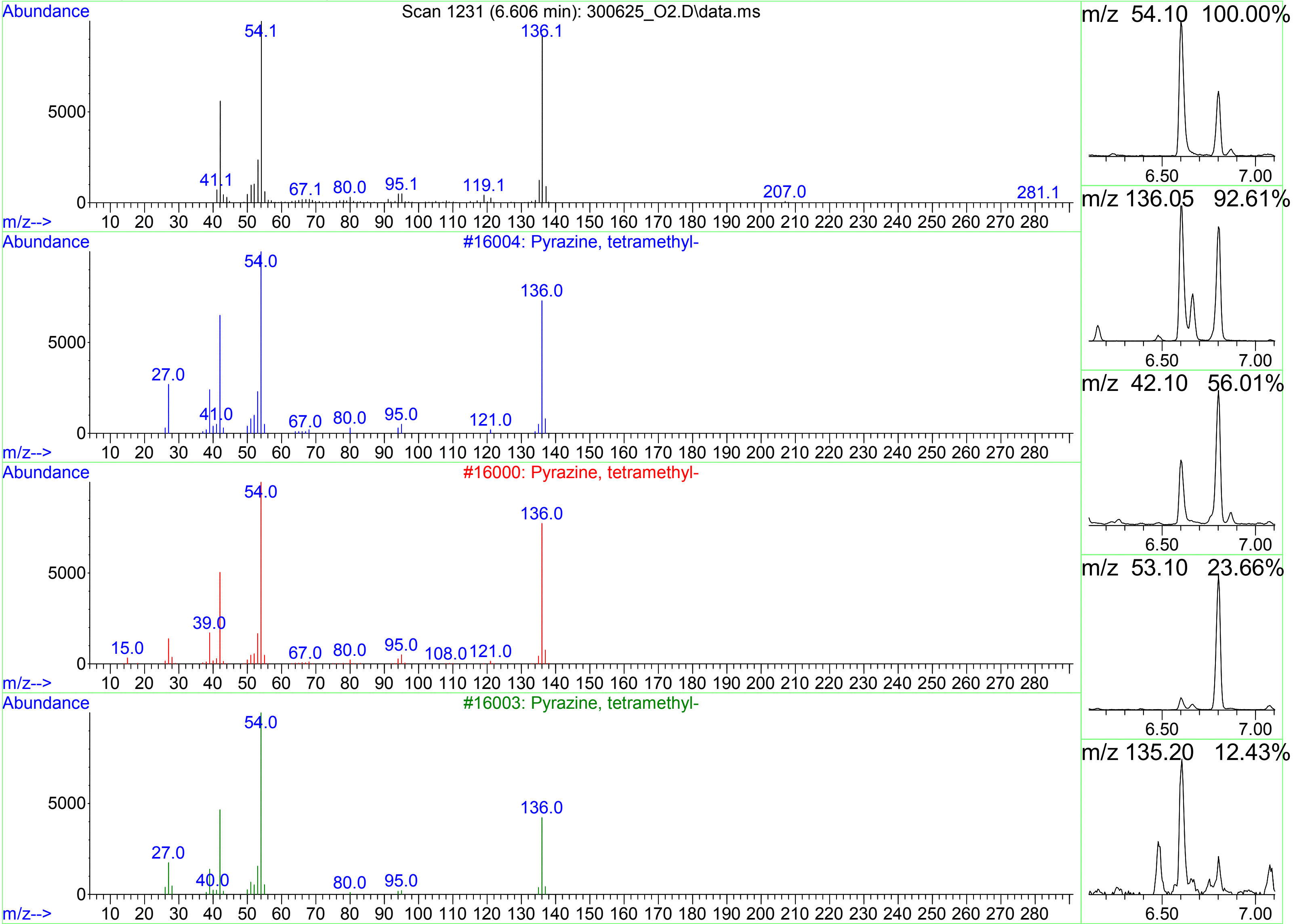

Data File: C:\msdchem\1\data\2025\Docentes\Sussulini\Romani\300625_O2.D
Sample :

Peak Number: 30 at 6.606 min Area: 4195379 Area % 0.73

The 3 best hits from each library.	Ref#	CAS#	Qual
C:\Database\NIST08.L			
1 Pyrazine, tetramethyl-	16004	001124-11-4	95
2 Pyrazine, tetramethyl-	16000	001124-11-4	94
3 Pyrazine, tetramethyl-	16003	001124-11-4	91

Unknown Spectrum based on Apex

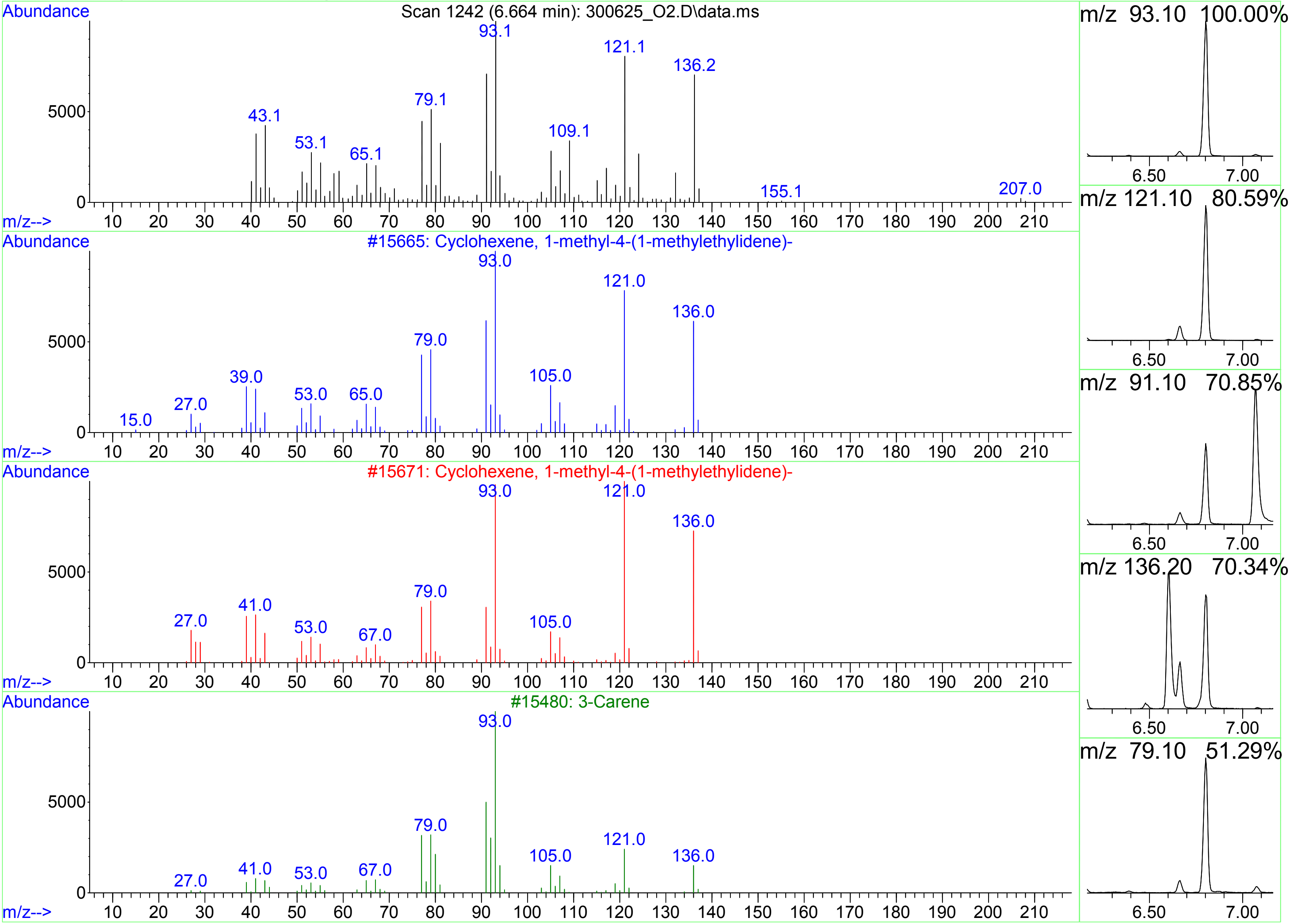

Data File: C:\msdchem\1\data\2025\Docentes\Sussulini\Romani\300625_O2.D
Sample :

Peak Number: 31 at 6.664 min Area: 6299709 Area % 1.10

The 3 best hits from each library.	Ref#	CAS#	Qual
C:\Database\NIST08.L			
1 Cyclohexene, 1-methyl-4-(1-methy...	15665	000586-62-9	93
2 Cyclohexene, 1-methyl-4-(1-methy...	15671	000586-62-9	90
3 3-Carene	15480	013466-78-9	90

Unknown Spectrum based on Apex

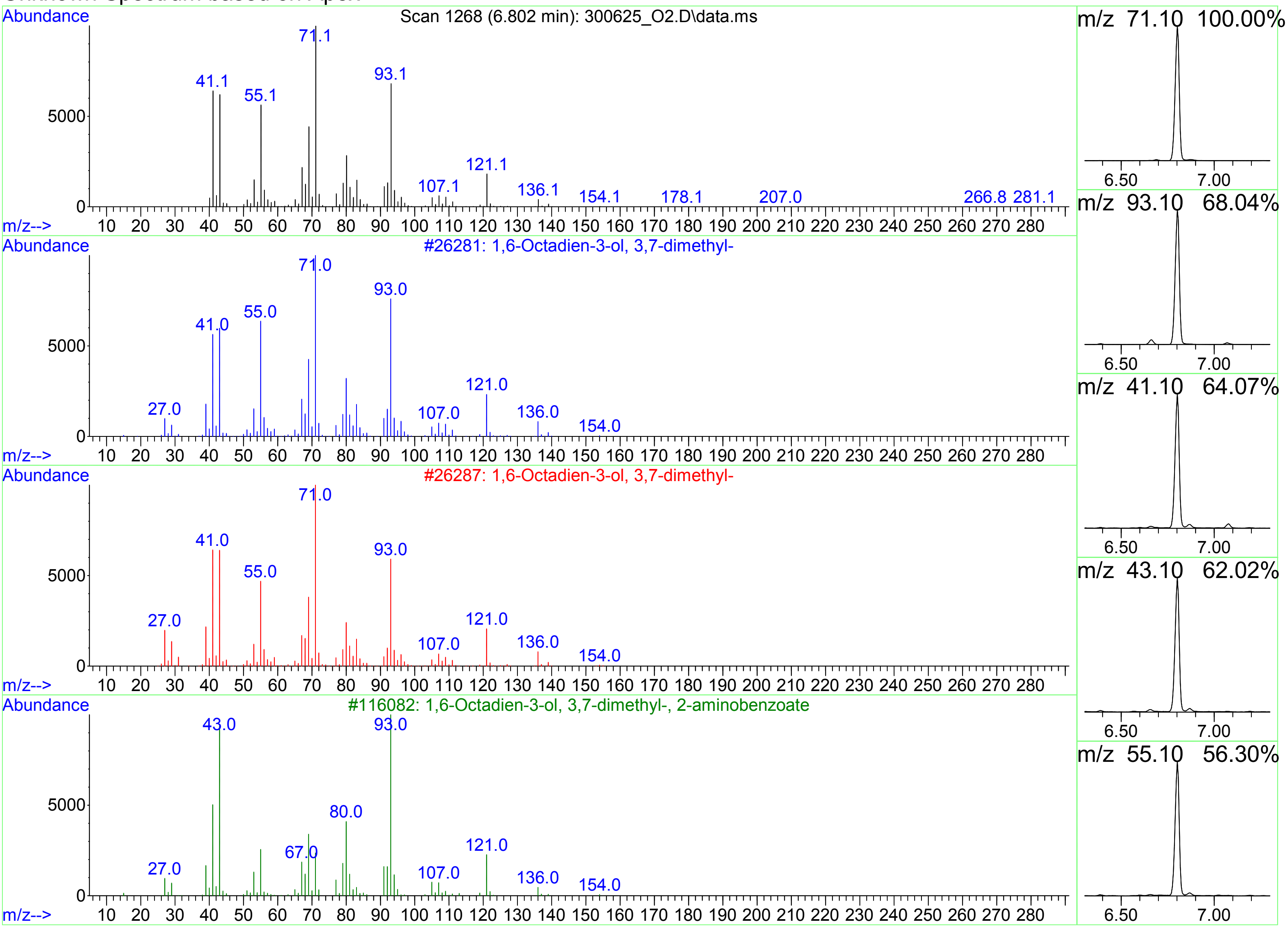

Data File: C:\msdchem\1\data\2025\Docentes\Sussulini\Romani\300625_O2.D
Sample :

Peak Number: 32 at 6.802 min Area: 126074182 Area % 22.06

The 3 best hits from each library.	Ref#	CAS#	Qual
C:\Database\NIST08.L			
1 1,6-Octadien-3-ol, 3,7-dimethyl-	26281	000078-70-6	97
2 1,6-Octadien-3-ol, 3,7-dimethyl-	26287	000078-70-6	80
3 1,6-Octadien-3-ol, 3,7-dimethyl-...	116082	007149-26-0	53

Library Search Report - ChemStation Integrator

Unknown Spectrum based on Apex

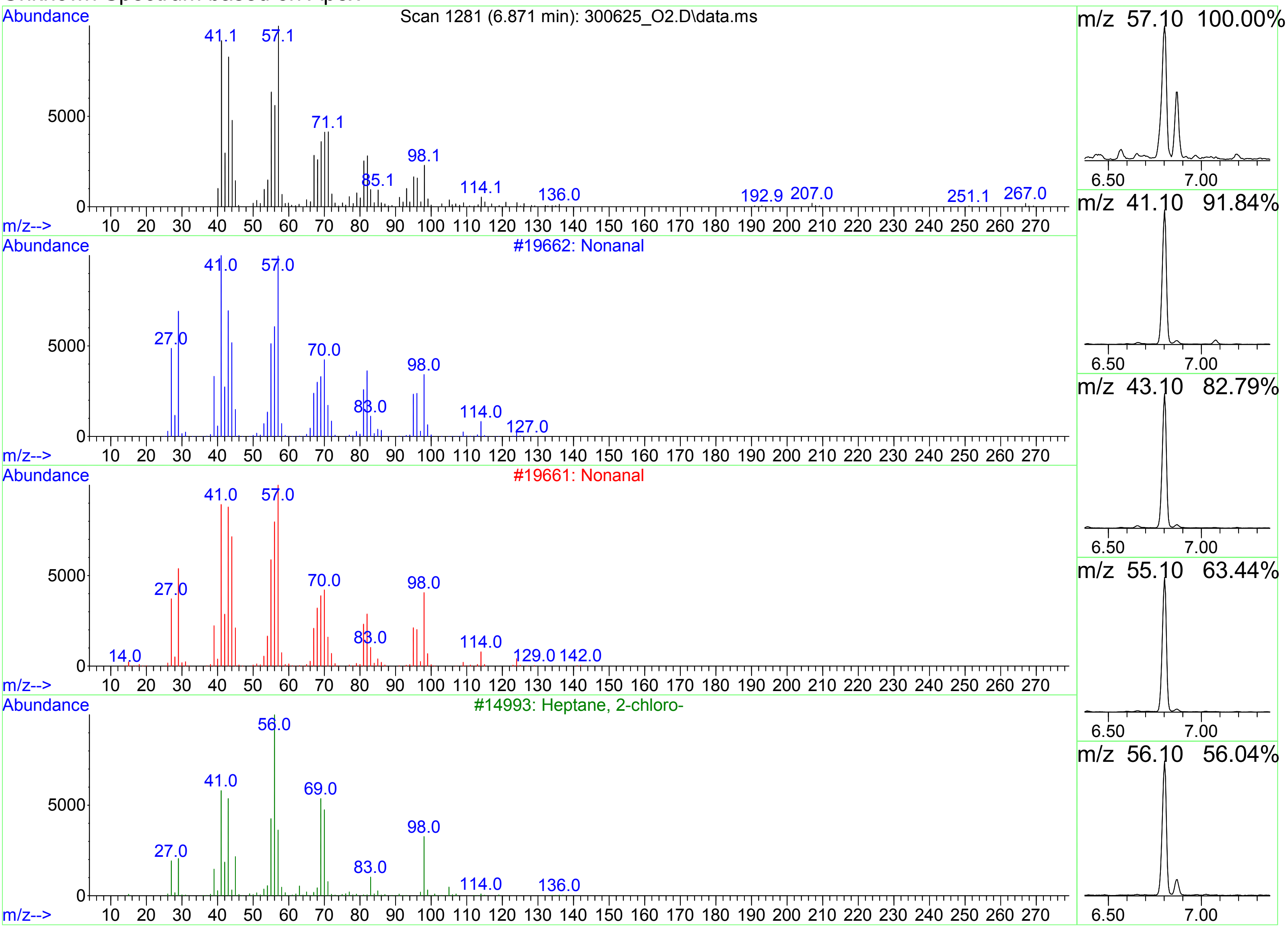

Data File: C:\msdchem\1\data\2025\Docentes\Sussulini\Romani\300625_O2.D
Sample :

Peak Number: 33 at 6.871 min Area: 4062822 Area % 0.71

The 3 best hits from each library.	Ref#	CAS#	Qual
C:\Database\NIST08.L			
1 Nonanal	19662	000124-19-6	64
2 Nonanal	19661	000124-19-6	59
3 Heptane, 2-chloro-	14993	001001-89-4	27

Unknown Spectrum based on Apex

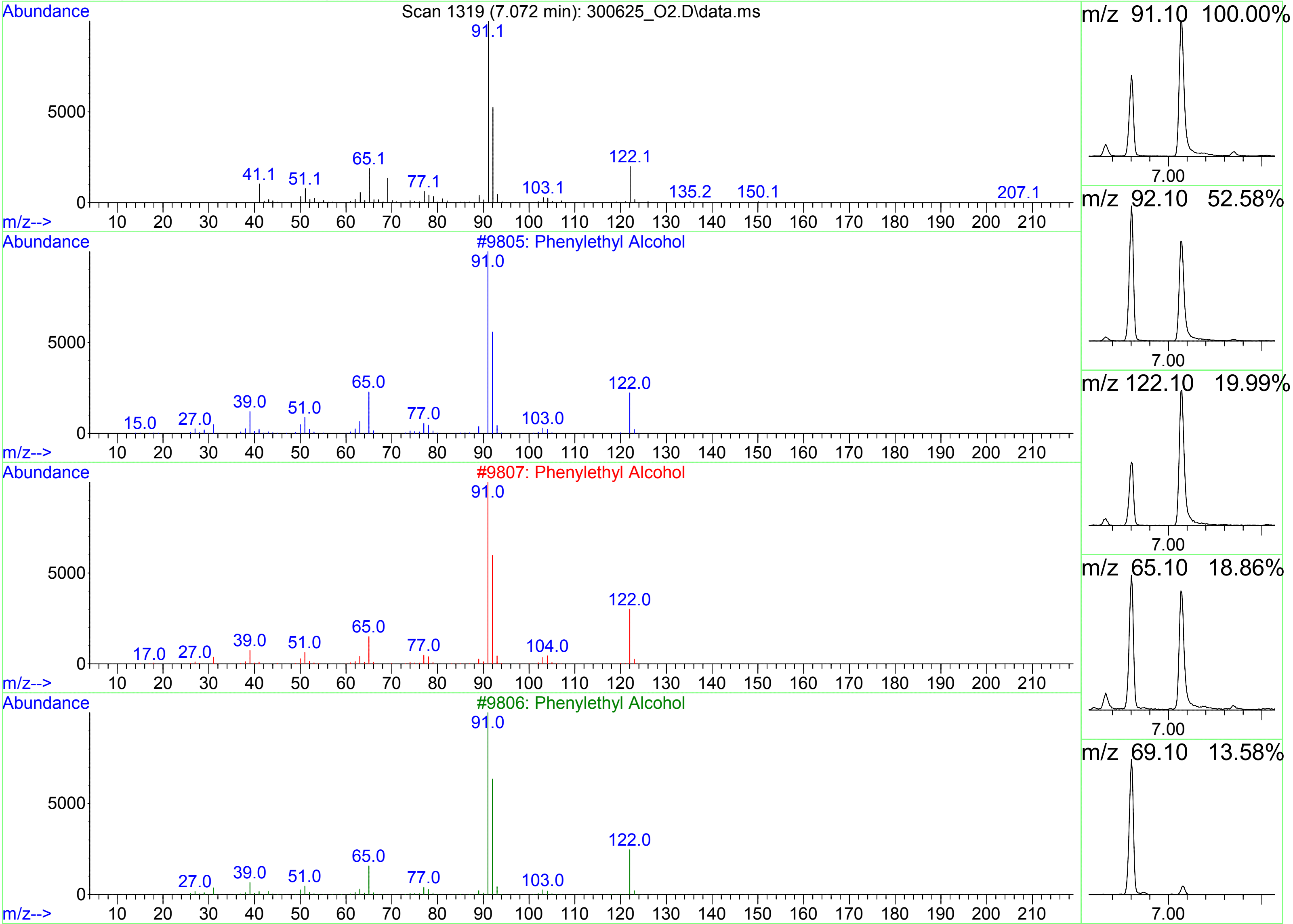

Data File: C:\msdchem\1\data\2025\Docentes\Sussulini\Romani\300625_O2.D
Sample :

Peak Number: 34 at 7.072 min Area: 12241635 Area % 2.14

The 3 best hits from each library.	Ref#	CAS#	Qual
C:\Database\NIST08.L			
1 Phenylethyl Alcohol	9805	000060-12-8	95
2 Phenylethyl Alcohol	9807	000060-12-8	93
3 Phenylethyl Alcohol	9806	000060-12-8	87

Unknown Spectrum based on Apex

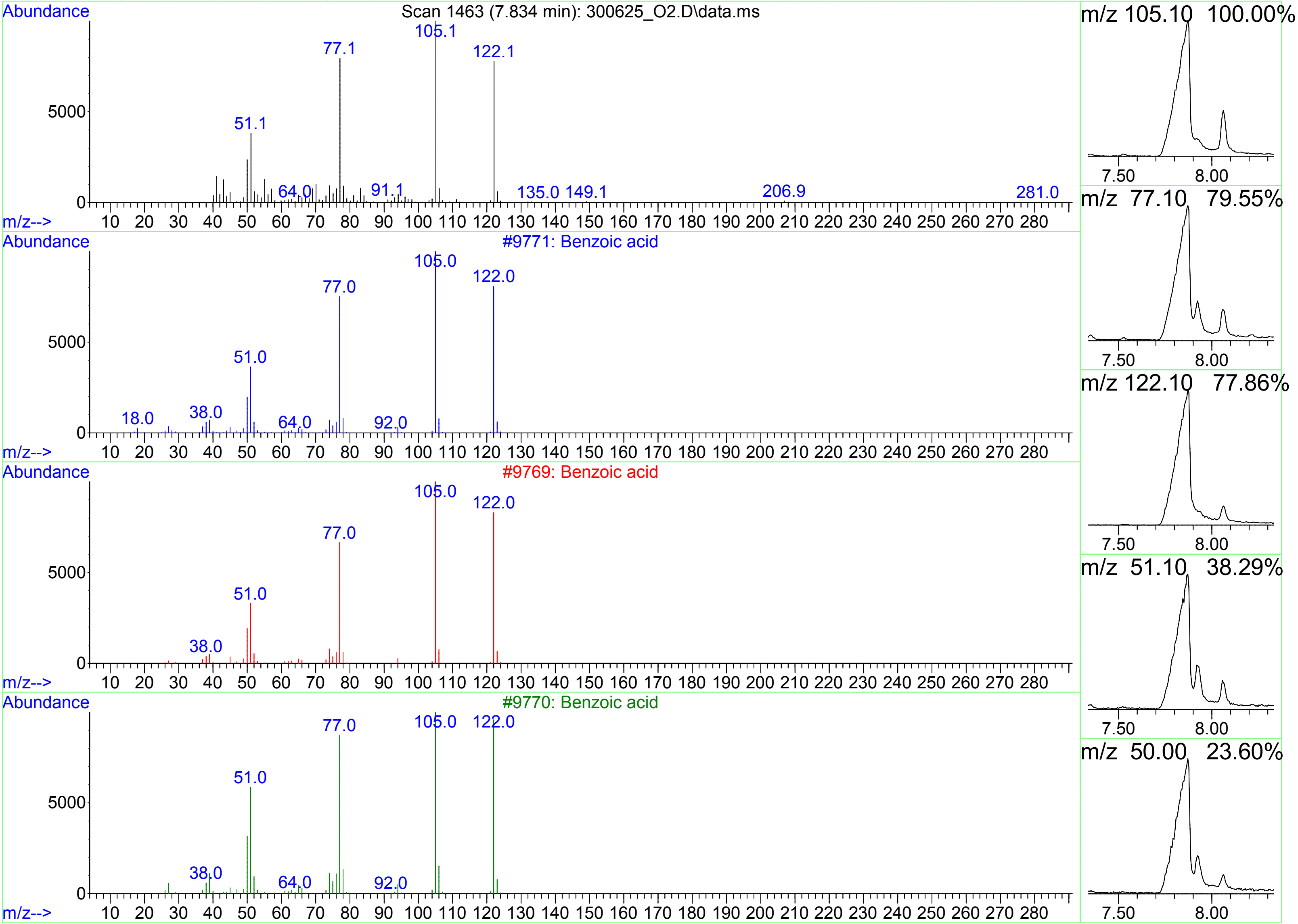

Data File: C:\msdchem\1\data\2025\Docentes\Sussulini\Romani\300625_O2.D
Sample :

Peak Number: 35 at 7.834 min Area: 12737652 Area % 2.23

The 3 best hits from each library.	Ref#	CAS#	Qual
C:\Database\NIST08.L			
1 Benzoic acid	9771	000065-85-0	97
2 Benzoic acid	9769	000065-85-0	93
3 Benzoic acid	9770	000065-85-0	93

Unknown Spectrum based on Apex

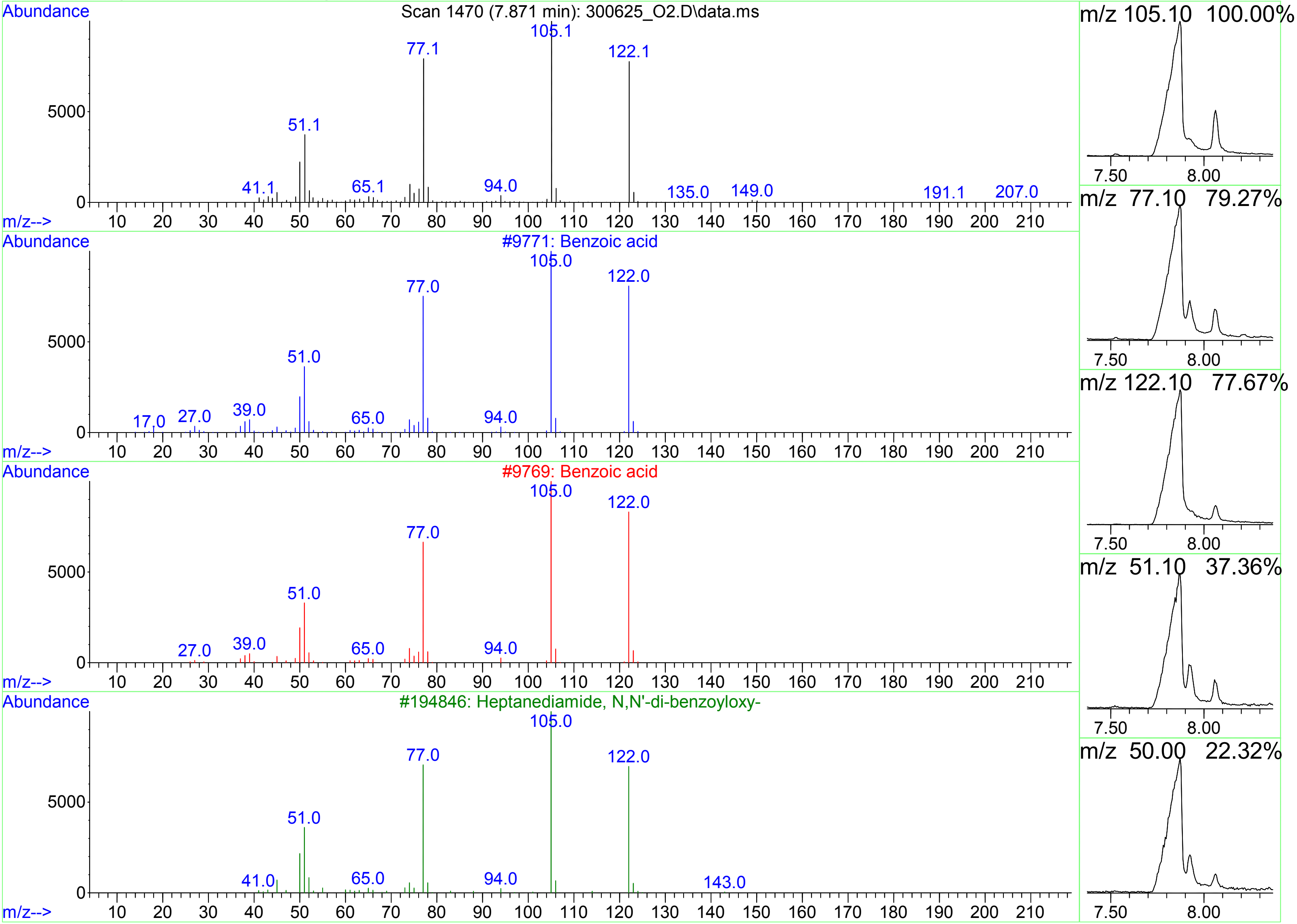

Data File: C:\msdchem\1\data\2025\Docentes\Sussulini\Romani\300625_O2.D
Sample :

Peak Number: 36 at 7.871 min Area: 7509831 Area % 1.31

The 3 best hits from each library. Ref# CAS# Qual

C:\Database\NIST08.L

1	Benzoic acid	9771	000065-85-0	97
2	Benzoic acid	9769	000065-85-0	96
3	Heptanediamide, N,N'-di-benzoyloxy-	194846	1000253-26-4	91

Library Search Report - ChemStation Integrator

Unknown Spectrum based on Apex

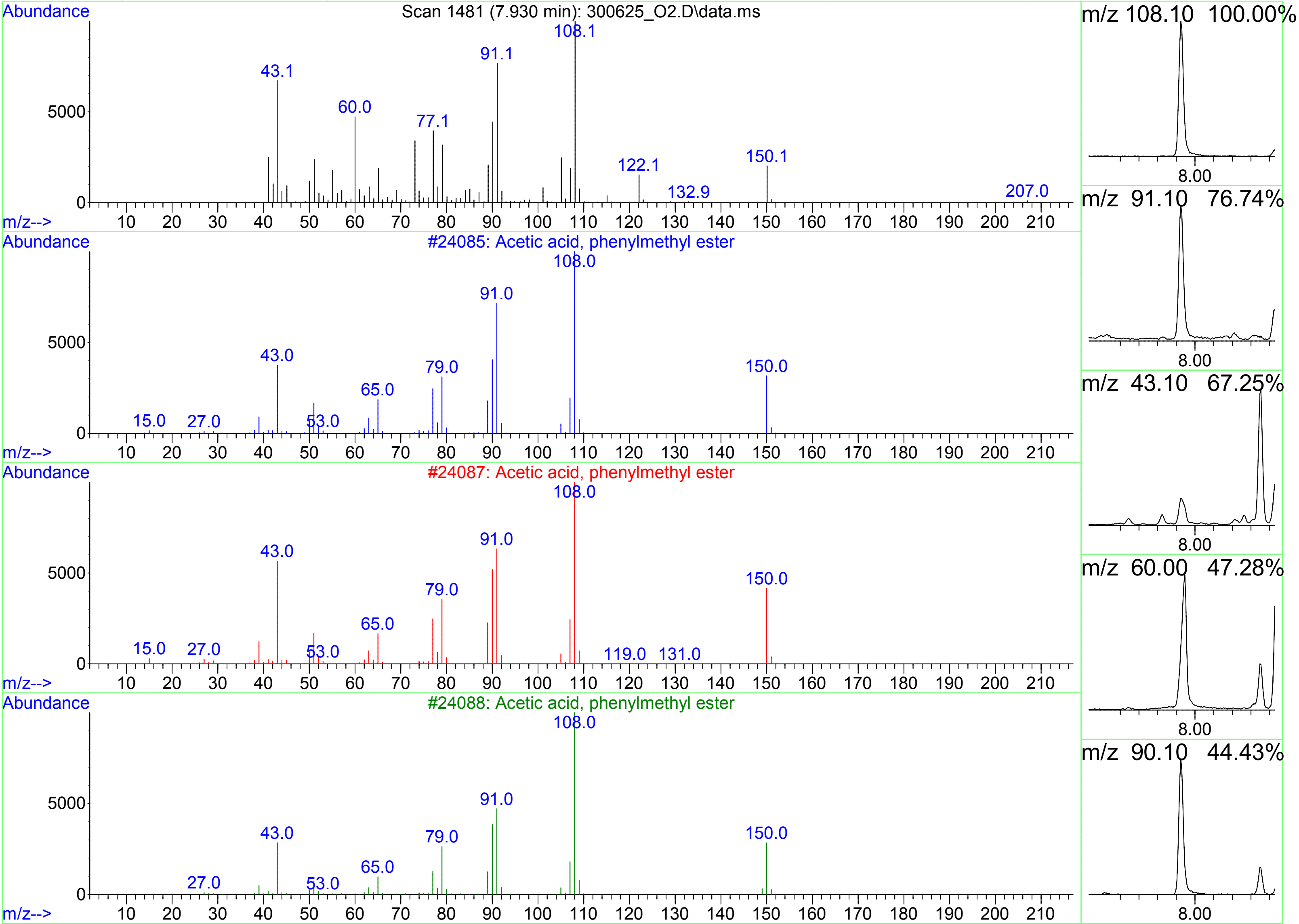

Data File: C:\msdchem\1\data\2025\Docentes\Sussulini\Romani\300625_O2.D
Sample :

Peak Number: 37 at 7.930 min Area: 9295794 Area % 1.63

The 3 best hits from each library.	Ref\#	CAS\#	Qual
C:\Database\NIST08.L			
1 Acetic acid, phenylmethyl ester	24085	000140-11-4	93
2 Acetic acid, phenylmethyl ester	24087	000140-11-4	92
3 Acetic acid, phenylmethyl ester	24088	000140-11-4	55

Library Search Report - ChemStation Integrator

Unknown Spectrum based on Apex

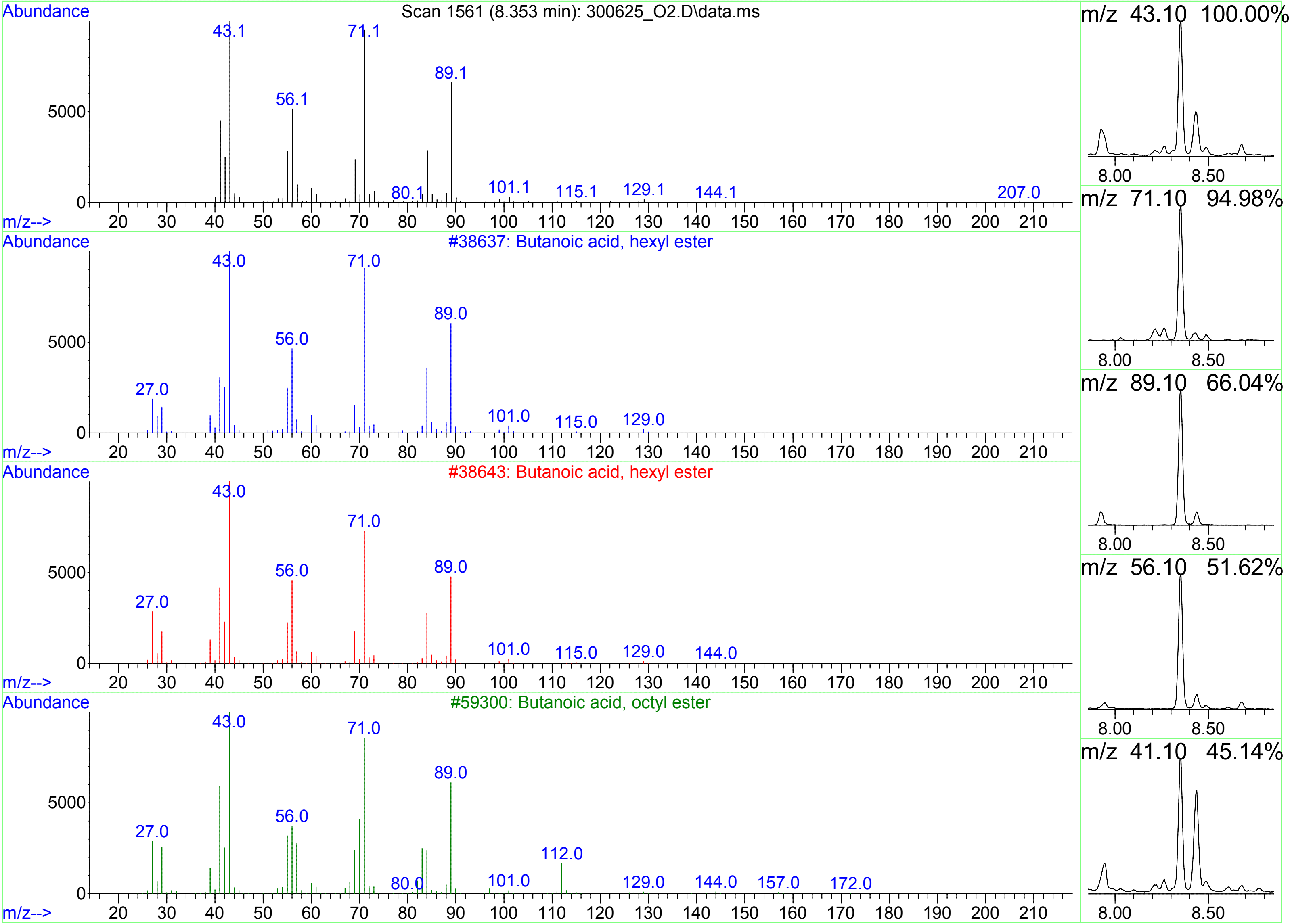

Data File: C:\msdchem\1\data\2025\Docentes\Sussulini\Romani\300625_O2.D
Sample :

Peak Number: 38 at 8.353 min Area: 15056871 Area % 2.63

The 3 best hits from each library.	Ref#	CAS#	Qual
C:\Database\NIST08.L			
1 Butanoic acid, hexyl ester	38637	002639-63-6	91
2 Butanoic acid, hexyl ester	38643	002639-63-6	91
3 Butanoic acid, octyl ester	59300	000110-39-4	90

Unknown Spectrum based on Apex

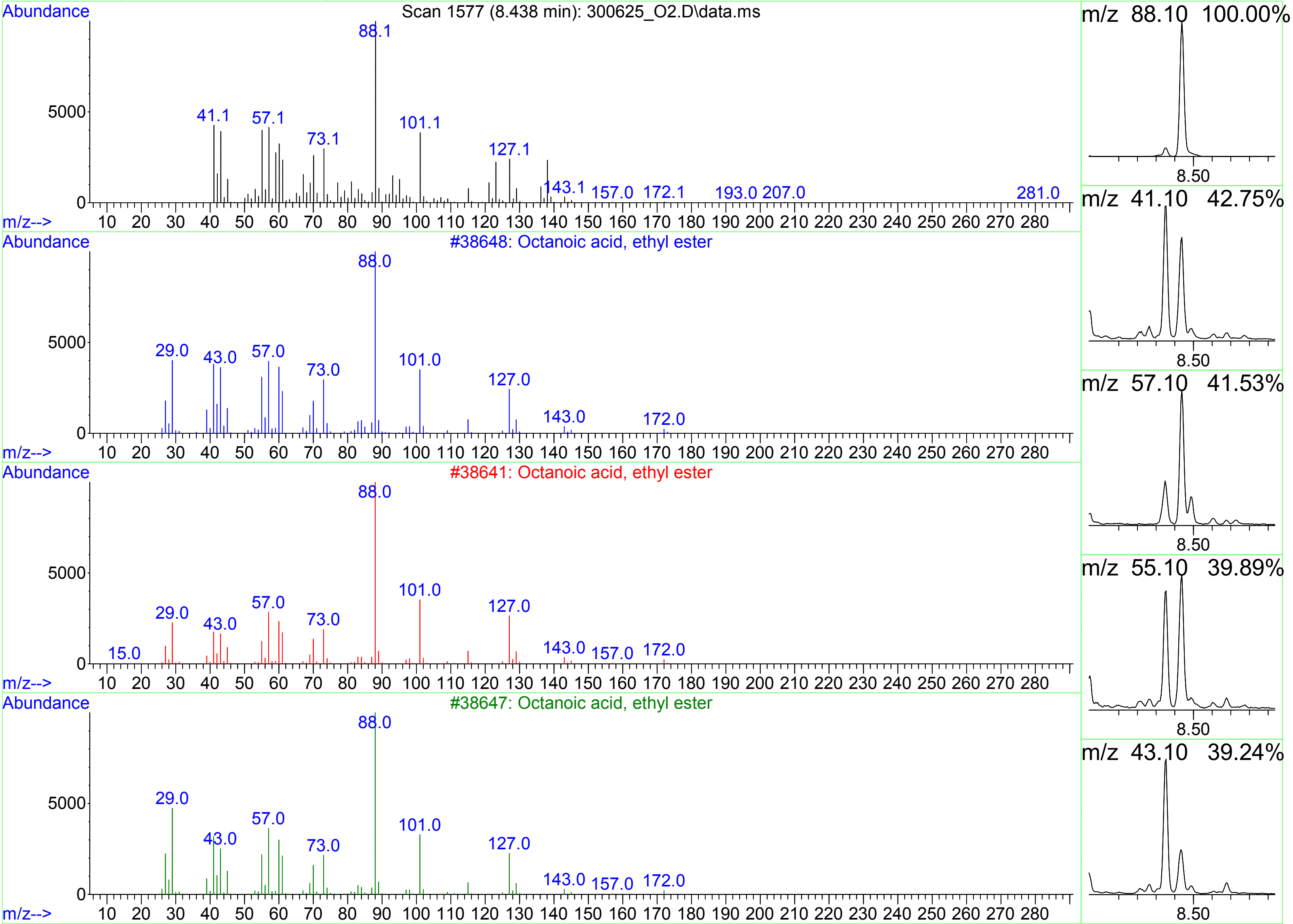

Data File: C:\msdchem\1\data\2025\Docentes\Sussulini\Romani\300625_O2.D
Sample :

Peak Number: 39 at 8.438 min Area: 20985684 Area % 3.67

The 3 best hits from each library.	Ref#	CAS#	Qual
C:\Database\NIST08.L			
1 Octanoic acid, ethyl ester	38648	000106-32-1	68
2 Octanoic acid, ethyl ester	38641	000106-32-1	64
3 Octanoic acid, ethyl ester	38647	000106-32-1	64

Library Search Report - ChemStation Integrator

Unknown Spectrum based on Apex

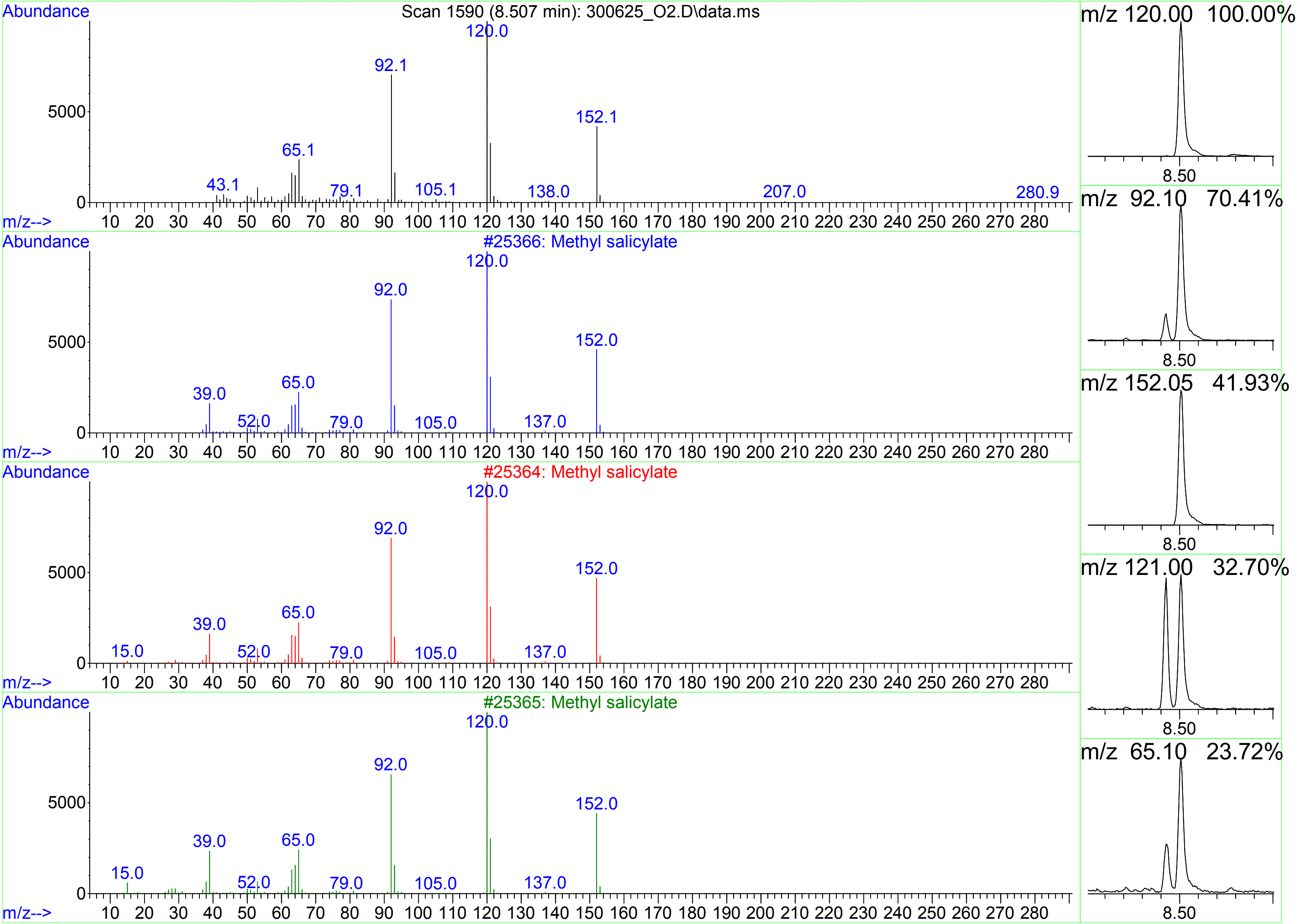

Data File: C:\msdchem\1\data\2025\Docentes\Sussulini\Romani\300625_O2.D
Sample :

Peak Number: 40 at 8.507 min Area: 7456850 Area % 1.30

The 3 best hits from each library.	Ref#	CAS#	Qual
C:\Database\NIST08.L			
1 Methyl salicylate	25366	000119-36-8	97
2 Methyl salicylate	25364	000119-36-8	96
3 Methyl salicylate	25365	000119-36-8	96

Unknown Spectrum based on Apex

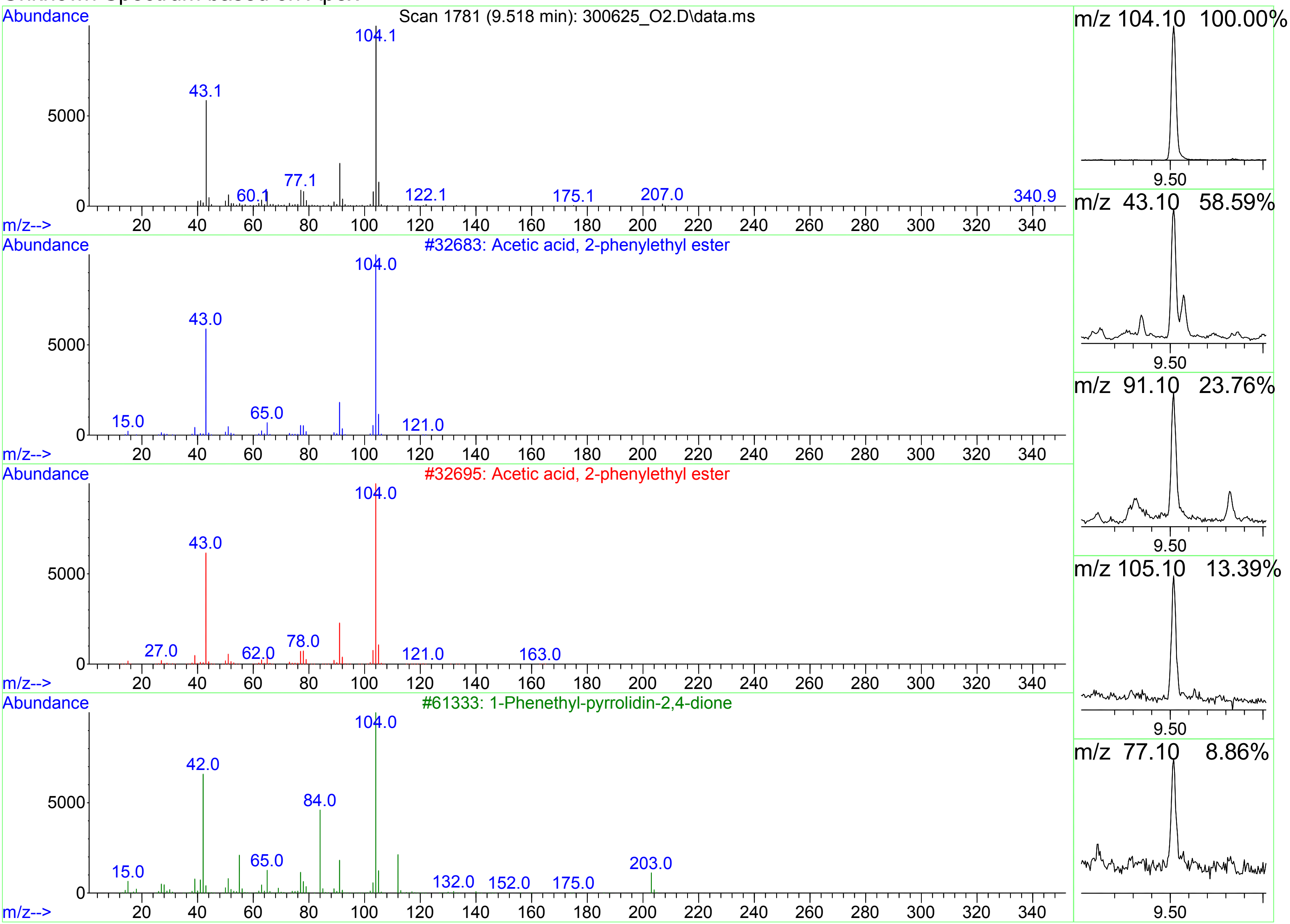

Data File: C:\msdchem\1\data\2025\Docentes\Sussulini\Romani\300625_O2.D
Sample :

Peak Number: 41 at 9.518 min Area: 2335430 Area % 0.41

The 3 best hits from each library. Ref\# CAS\# Qual

C:\Database\NIST08.L

1	Acetic acid, 2-phenylethyl ester	32683	000103-45-7	86
2	Acetic acid, 2-phenylethyl ester	32695	000103-45-7	86
3	1-Phenethyl-pyrrolidin-2,4-dione	61333	1000287-25-2	83

Library Search Report - ChemStation Integrator

Unknown Spectrum based on Apex

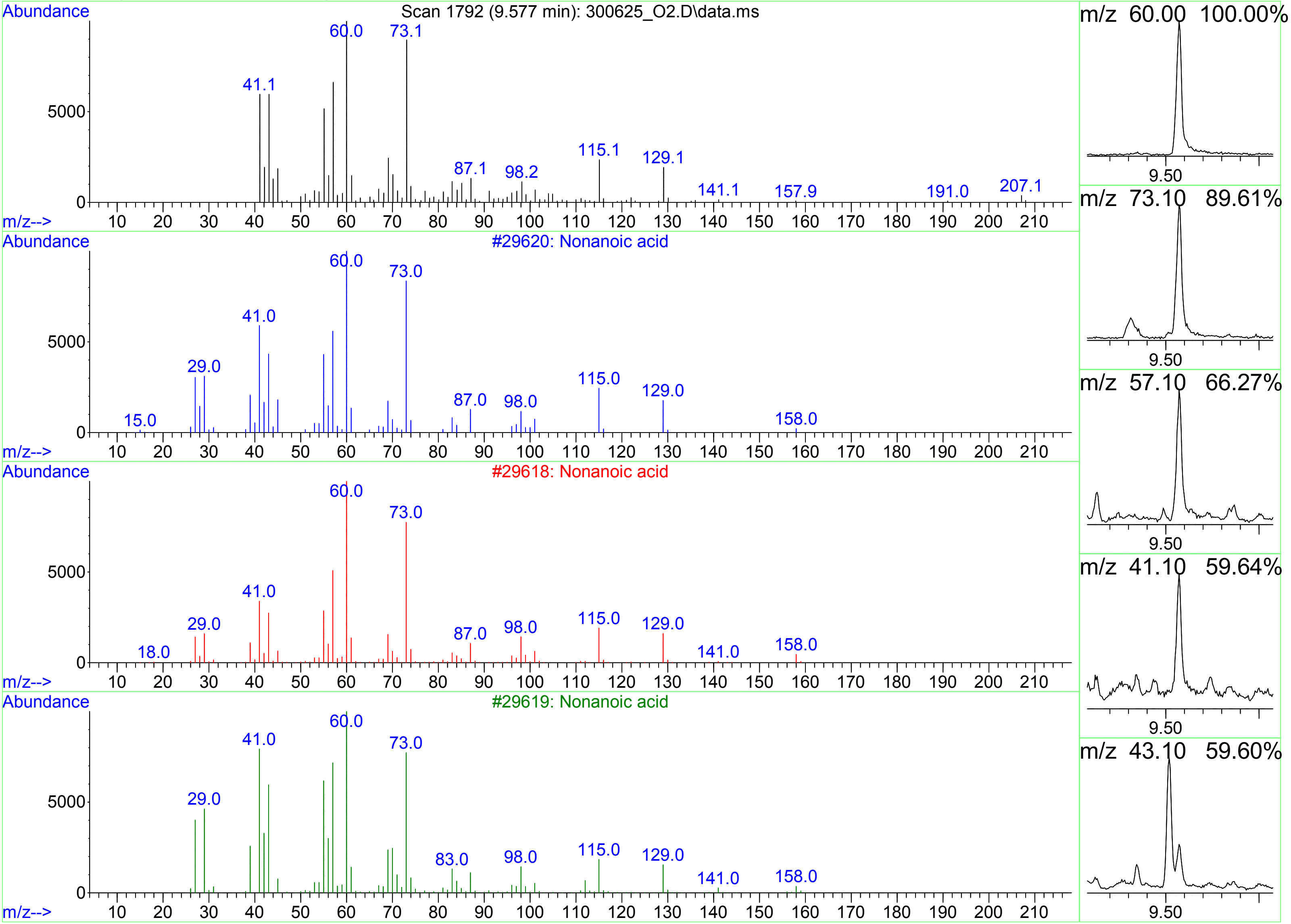

Data File: C:\msdchem\1\data\2025\Docentes\Sussulini\Romani\300625_O2.D
Sample :

Peak Number: 42 at 9.577 min Area: 2619895 Area % 0.46

The 3 best hits from each library.	Ref\#	CAS\#	Qual
C:\Database\NIST08.L			
1 Nonanoic acid	29620	000112-05-0	72
2 Nonanoic acid	29618	000112-05-0	72
3 Nonanoic acid	29619	000112-05-0	64

Library Search Report - ChemStation Integrator

Unknown Spectrum based on Apex

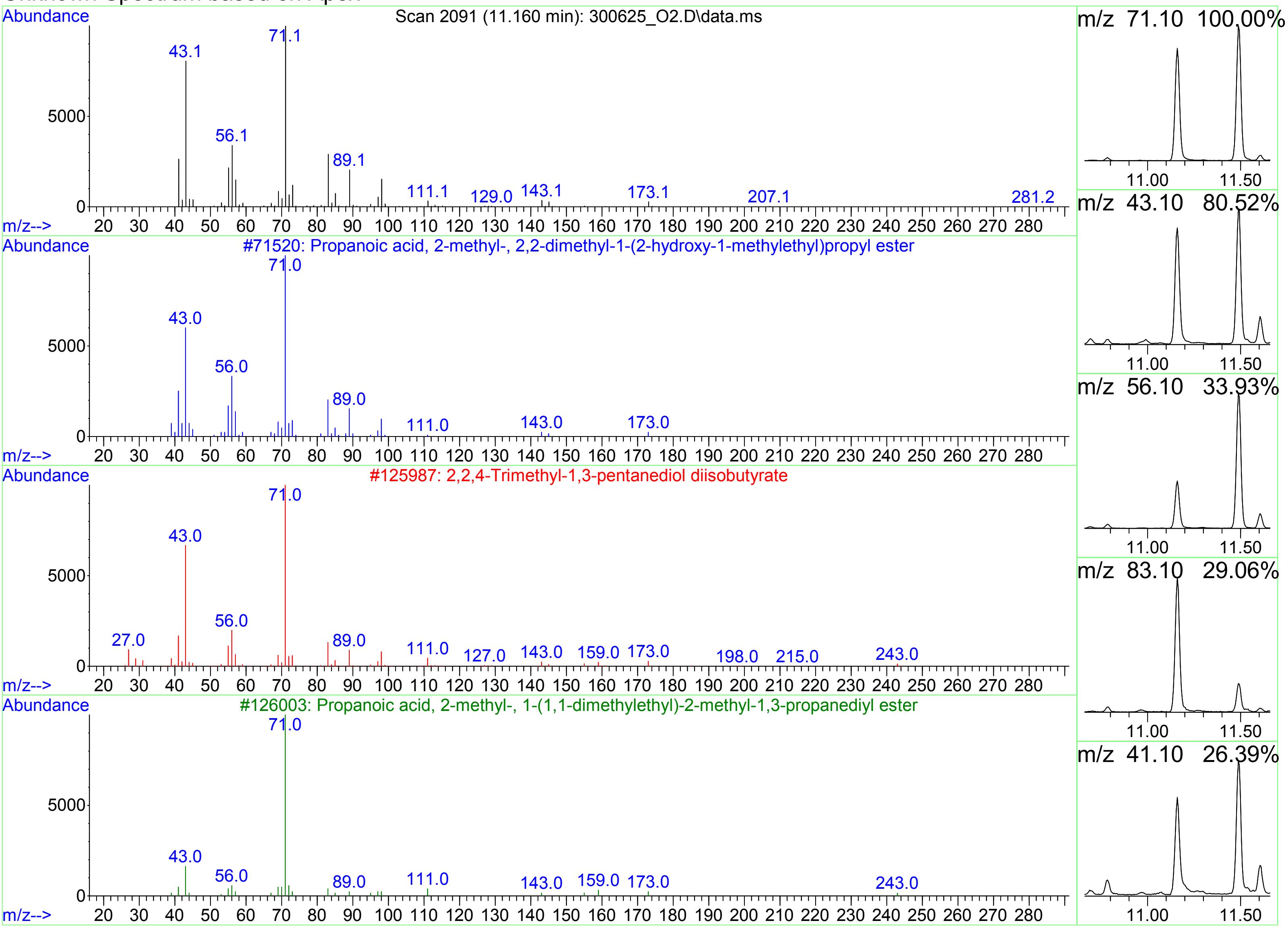

Data File: C:\msdchem\1\data\2025\Docentes\Sussulini\Romani\300625_O2.D
Sample :

Peak Number: 43 at 11.160 min Area: 13939815 Area % 2.44

The 3 best hits from each library.	Ref#	CAS#	Qual
C:\Database\NIST08.L			
1 Propanoic acid, 2-methyl-, 2,2-d...	71520	074367-33-2	90
2 2,2,4-Trimethyl-1,3-pentanediol ...	125987	006846-50-0	64
3 Propanoic acid, 2-methyl-, 1-(1,...	126003	074381-40-1	47

Library Search Report - ChemStation Integrator

Unknown Spectrum based on Apex

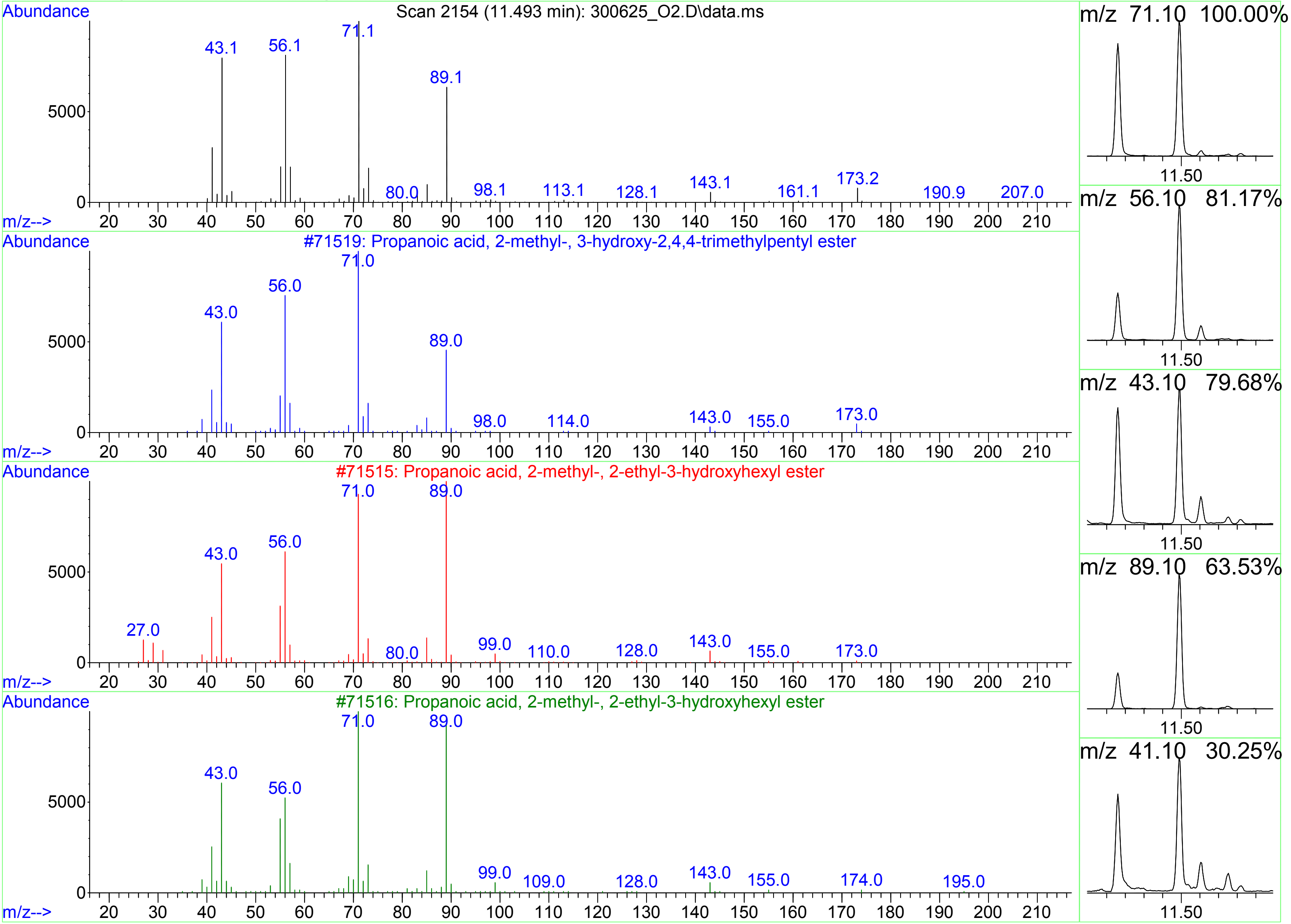

Data File: C:\msdchem\1\data\2025\Docentes\Sussulini\Romani\300625_O2.D
Sample :

Peak Number: 44 at 11.493 min Area: 22393839 Area % 3.92

The 3 best hits from each library.	Ref\#	CAS\#	Qual
C:\Database\NIST08.L			
1 Propanoic acid, 2-methyl-, 3-hyd...	71519	074367-34-3	90
2 Propanoic acid, 2-methyl-, 2-eth...	71515	074367-31-0	83
3 Propanoic acid, 2-methyl-, 2-eth...	71516	074367-31-0	72

Library Search Report - ChemStation Integrator

Unknown Spectrum based on Apex

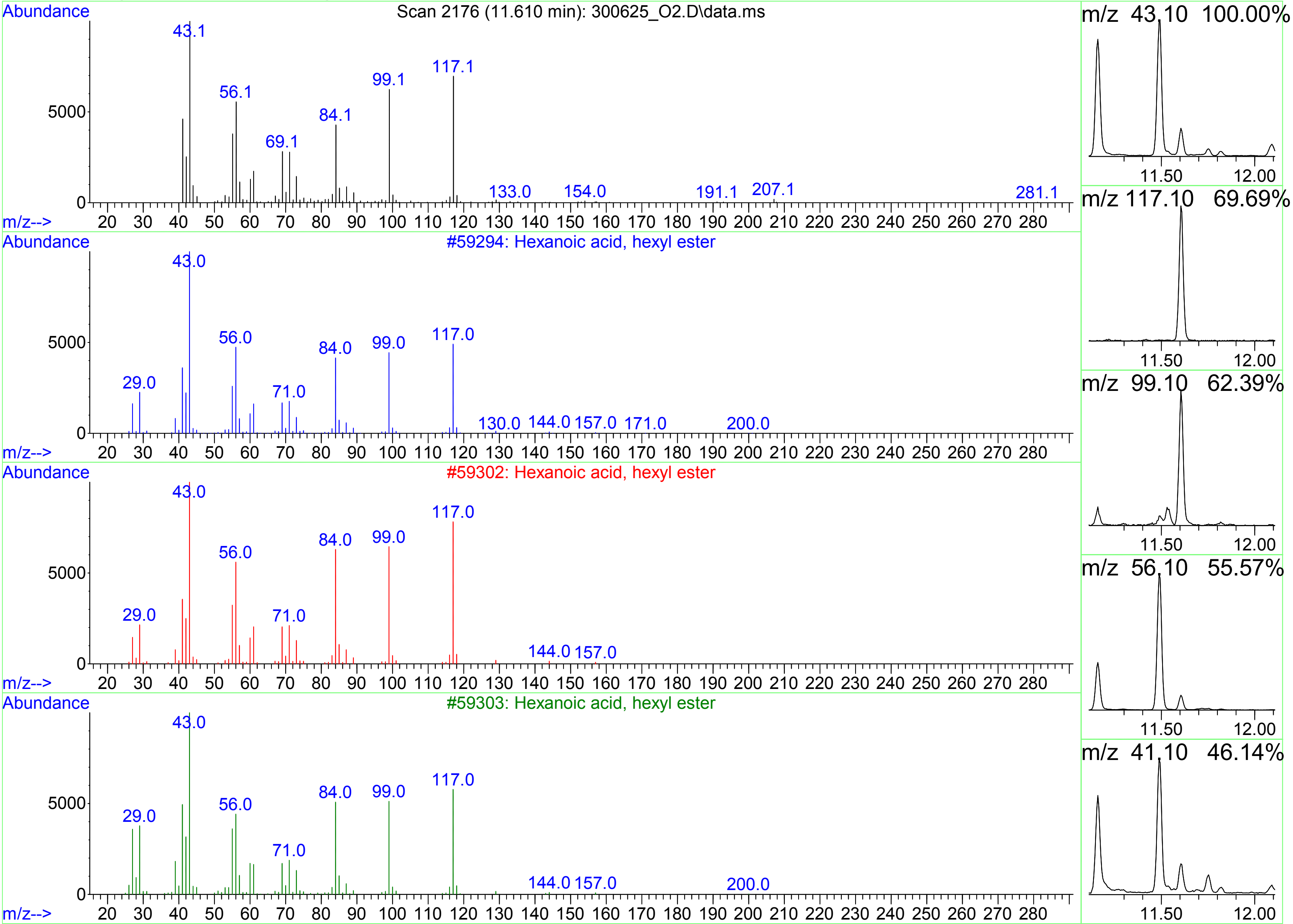

Data File: C:\msdchem\1\data\2025\Docentes\Sussulini\Romani\300625_O2.D
Sample :

Peak Number: 45 at 11.610 min Area: 4176162 Area % 0.73

The 3 best hits from each library.	Ref#	CAS#	Qual
C:\Database\NIST08.L			
1 Hexanoic acid, hexyl ester	59294	006378-65-0	91
2 Hexanoic acid, hexyl ester	59302	006378-65-0	87
3 Hexanoic acid, hexyl ester	59303	006378-65-0	80

Unknown Spectrum based on Apex

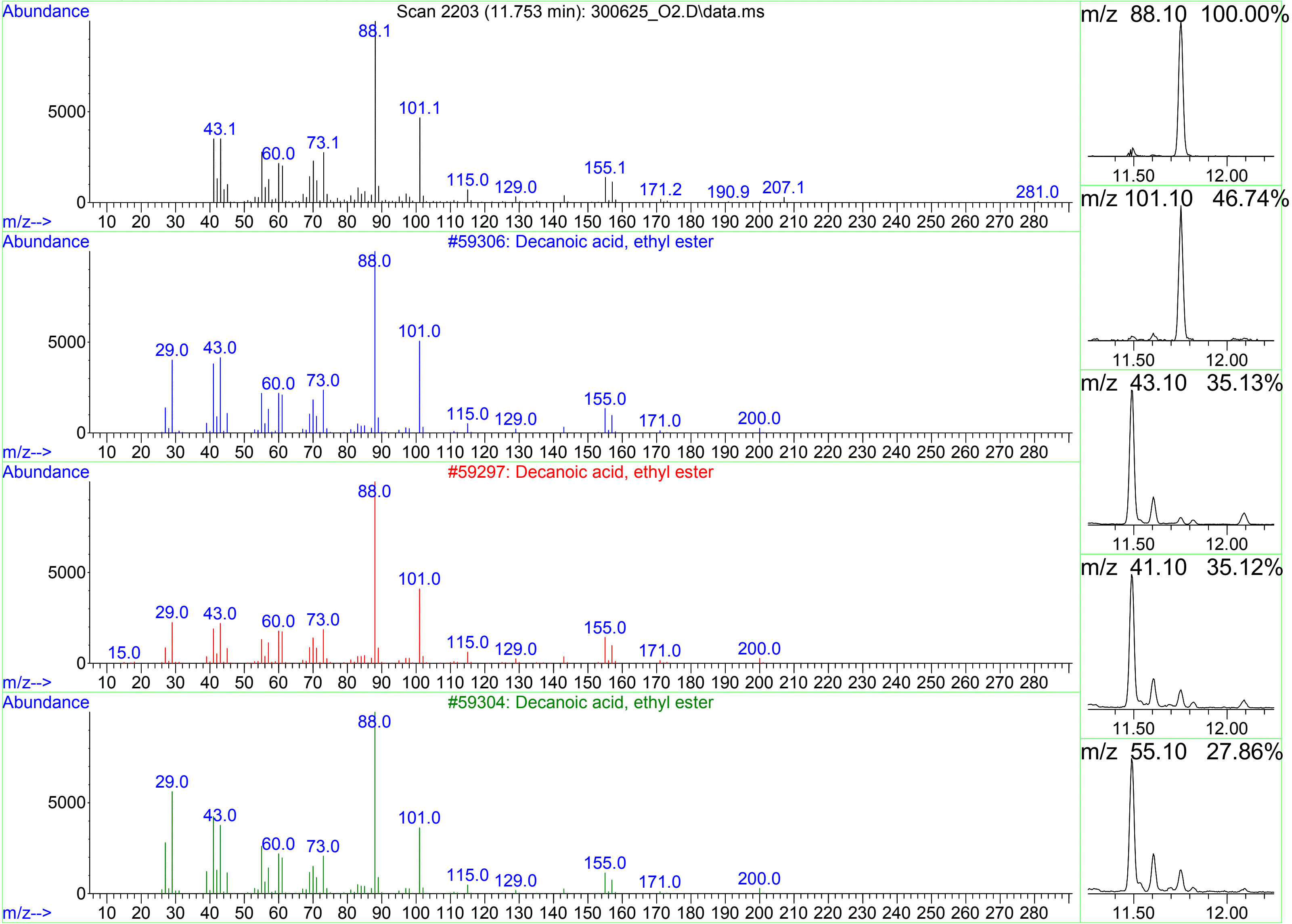

Data File: C:\msdchem\1\data\2025\Docentes\Sussulini\Romani\300625_O2.D
Sample :

Peak Number: 46 at 11.753 min Area: 3044741 Area % 0.53

The 3 best hits from each library.	Ref#	CAS#	Qual
C:\Database\NIST08.L			
1 Decanoic acid, ethyl ester	59306	000110-38-3	94
2 Decanoic acid, ethyl ester	59297	000110-38-3	87
3 Decanoic acid, ethyl ester	59304	000110-38-3	87

Unknown Spectrum based on Apex

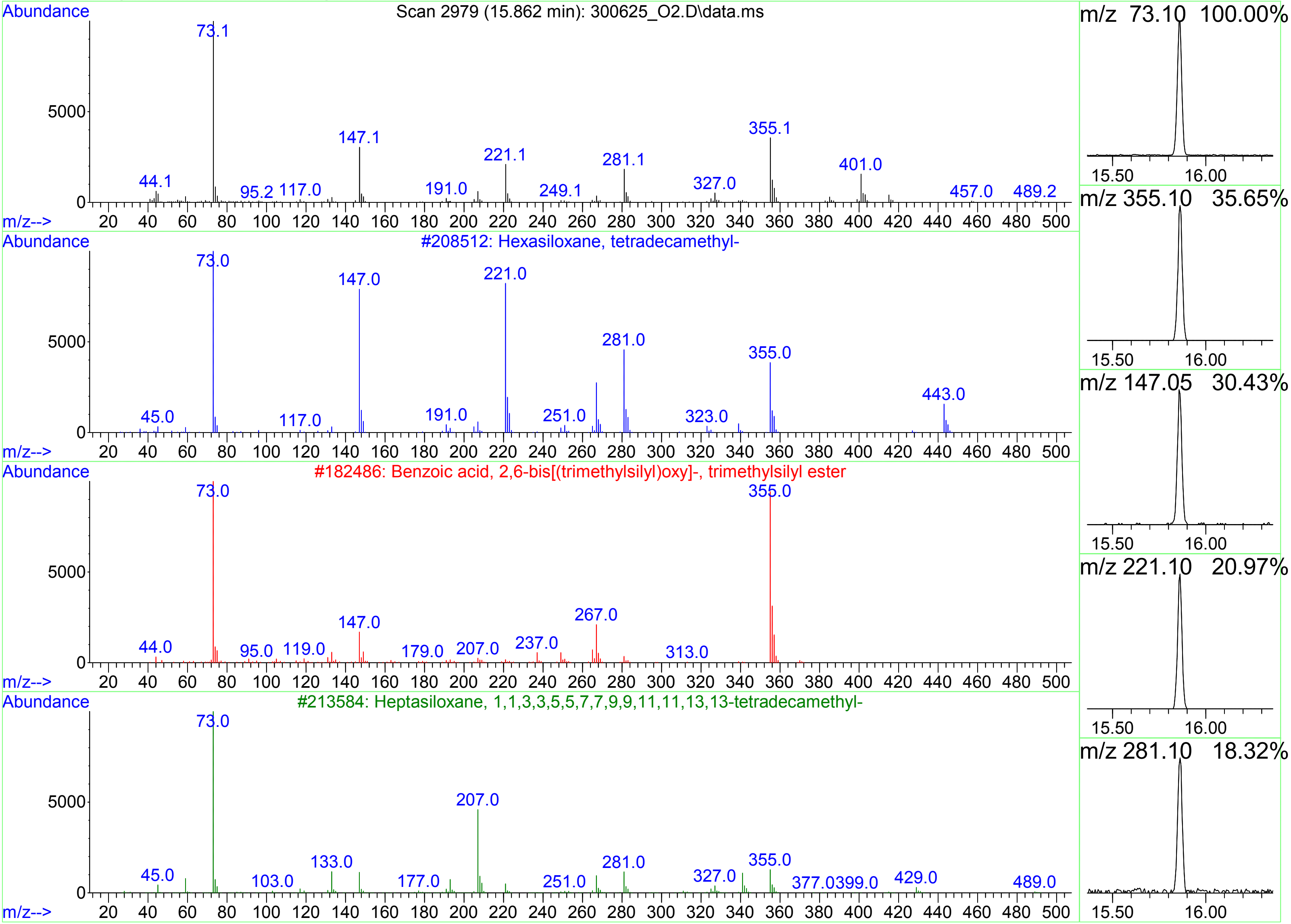

Data File: C:\msdchem\1\data\2025\Docentes\Sussulini\Romani\300625_O2.D
Sample :

Peak Number: 47 at 15.862 min Area: 2663208 Area % 0.47

The 3 best hits from each library.	Ref#	CAS#	Qual
C:\Database\NIST08.L			
1 Hexasiloxane, tetradecamethyl-	208512	000107-52-8	37
2 Benzoic acid, 2,6-bis[(trimethyl...	182486	003782-85-2	32
3 Heptasiloxane, 1,1,3,3,5,5,7,7,9...	213584	019095-23-9	17

Library Search Report - ChemStation Integrator

Unknown Spectrum based on Apex

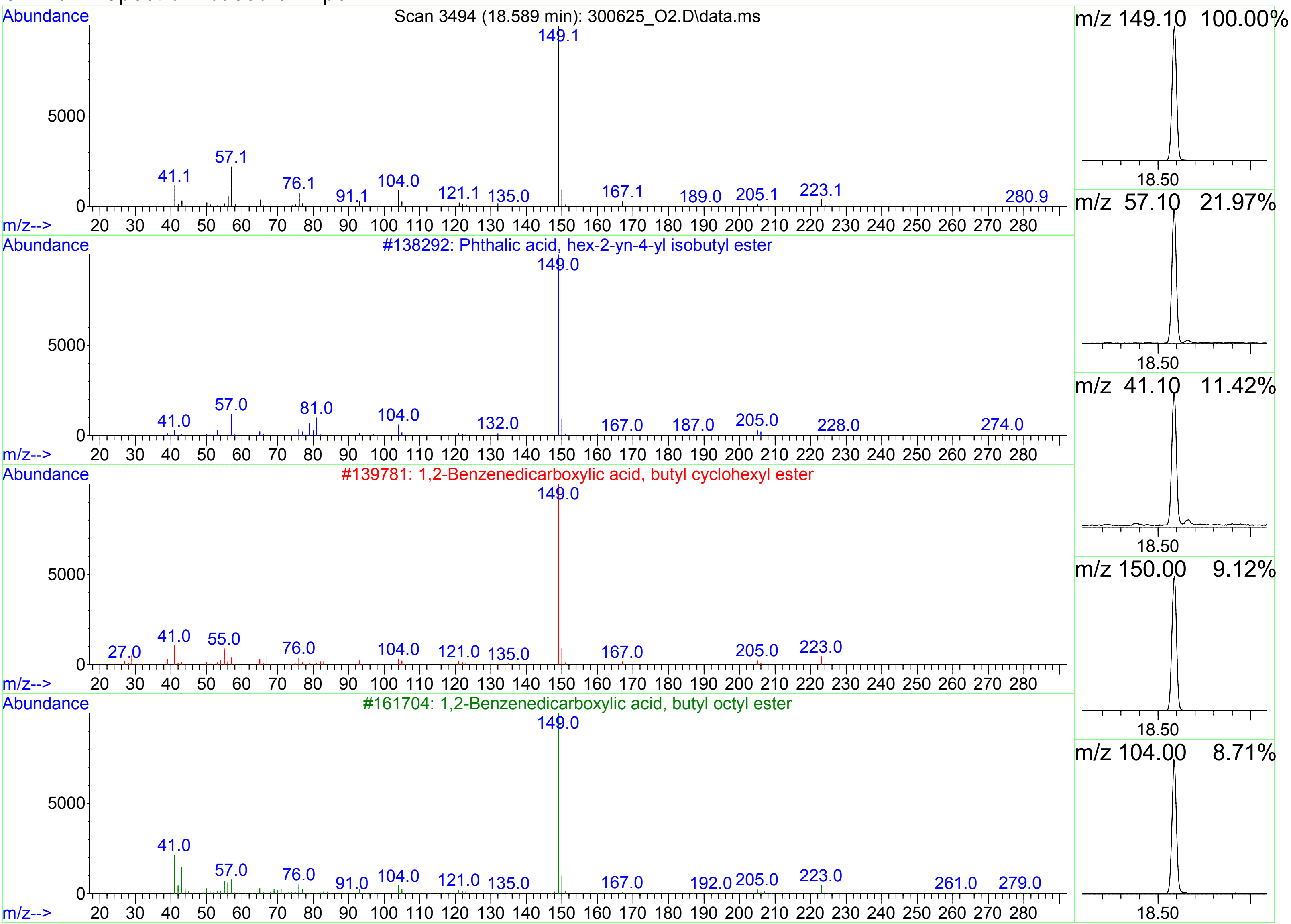

Data File: C:\msdchem\1\data\2025\Docentes\Sussulini\Romani\300625_O2.D
Sample :

Peak Number: 48 at 18.589 min Area: 17063137 Area % 2.99

The 3 best hits from each library. Ref# CAS# Qual

C:\Database\NIST08.L

1	Phthalic acid, hex-2-yn-4-yl iso...	138292	1000315-19-9	78
2	1,2-Benzenedicarboxylic acid, bu...	139781	000084-64-0	78
3	1,2-Benzenedicarboxylic acid, bu...	161704	000084-78-6	78

Unknown Spectrum based on Apex

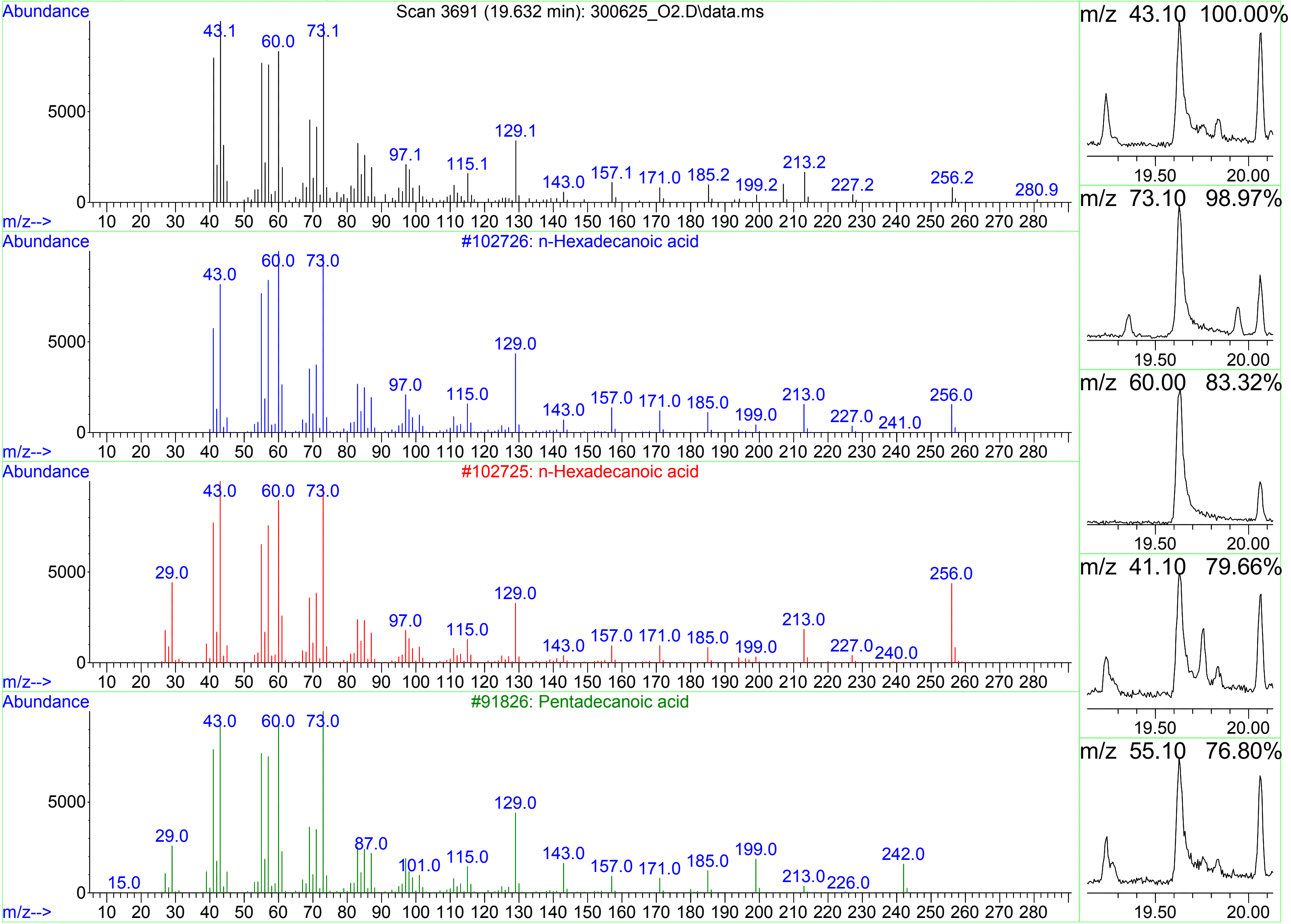

Data File: C:\msdchem\1\data\2025\Docentes\Sussulini\Romani\300625_O2.D
Sample :

Peak Number: 49 at 19.632 min Area: 2674079 Area % 0.47

The 3 best hits from each library.	Ref#	CAS#	Qual
C:\Database\NIST08.L			
1 n-Hexadecanoic acid	102726	000057-10-3	97
2 n-Hexadecanoic acid	102725	000057-10-3	94
3 Pentadecanoic acid	91826	001002-84-2	87

Library Search Report - ChemStation Integrator

Unknown Spectrum based on Apex

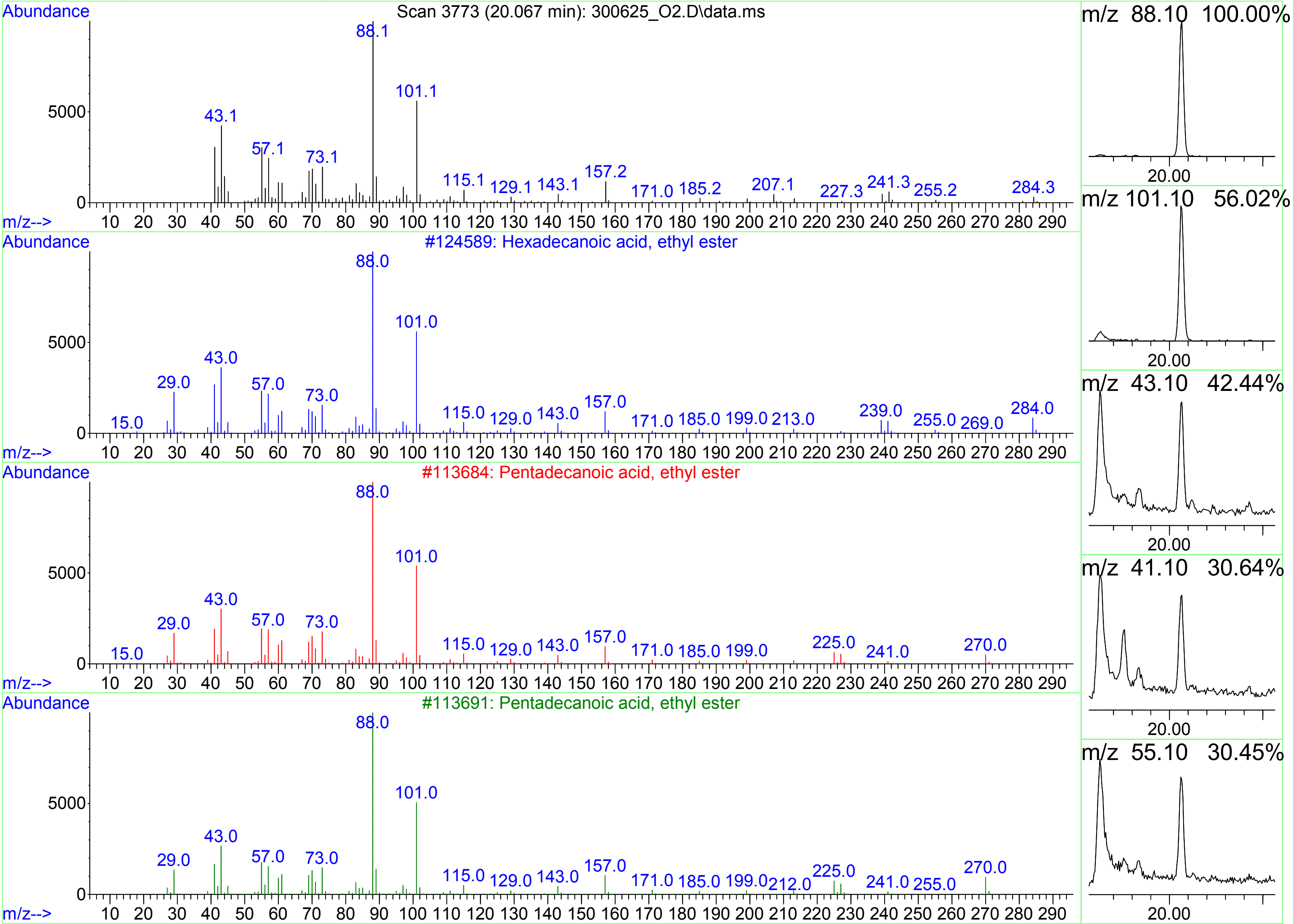

Data File: C:\msdchem\1\data\2025\Docentes\Sussulini\Romani\300625_O2.D
Sample :

Peak Number: 50 at 20.067 min Area: 2261699 Area % 0.40

The 3 best hits from each library.	Ref#	CAS#	Qual
C:\Database\NIST08.L			
1 Hexadecanoic acid, ethyl ester	124589	000628-97-7	98
2 Pentadecanoic acid, ethyl ester	113684	041114-00-5	91
3 Pentadecanoic acid, ethyl ester	113691	041114-00-5	90
